# Socioeconomic differences in the prevalence, treatment and control of major cardiometabolic risk factors by sex: a cross-sectional study of the UK Biobank

**DOI:** 10.1136/bmjph-2025-002961

**Published:** 2026-06-15

**Authors:** Rebecca K Kelly, Katie Harris, Paul Muntner, Mark Woodward

**Affiliations:** 1 The George Institute for Global Health, University of New South Wales, Sydney, New South Wales, Australia; 2 College of Health and Medicine, University of Tasmania, Hobart, Tasmania, Australia; 3 Department of Epidemiology, University of Alabama at Birmingham, Birmingham, Alabama, USA; 4 The George Institute for Global Health, Imperial College London, London, UK; 5 Nuffield Department of Women’s and Reproductive Health, University of Oxford, Oxford, UK

**Keywords:** Cardiovascular Diseases, Epidemiology, Demography

## Abstract

**Introduction:**

Social deprivation is related to cardiovascular risk, but the prevalence of cardiometabolic risk factors by deprivation and sex is less explored. We addressed this in a large UK cohort.

**Methods:**

500 769 UK Biobank participants (54.4% women) with ≥1 baseline risk factor measured were included. We examined differences in risk factors, including treatment and control, by socioeconomic status (Townsend score fifths) and sex.

**Results:**

Total cholesterol, low-density lipoprotein cholesterol (LDL-C) and high-density lipoprotein cholesterol were lower with greater deprivation and systolic blood pressure was lower, while C-reactive protein was higher. Body mass index, waist circumference and triglycerides were also higher with greater deprivation, with larger differences in women than men. Corresponding social differences in glycated haemoglobin and glucose were higher in men than in women while estimated glomerular filtration rate was higher in men only. The prevalence of hypertension and chronic kidney disease was higher with greater deprivation (% difference between least and most deprived (95% CI 5.1 (4.7 to 5.6) and 0.9 (0.8 to 1.1), respectively), but varied by sex for smoking (12.1 (11.6 to 12.5) vs 15.4 (14.9 to 15.9) in women vs men, respectively), obesity (13.7 (13.1 to 14.2) vs 8.2 (7.7 to 8.8)) and diabetes (2.6 (2.3 to 2.8) vs 3.8 (3.4 to 4.1)). Treatment and control of hypertension and dyslipidaemia were higher with greater deprivation.

**Conclusions:**

Apart from total cholesterol and LDL-C, greater social deprivation relates to worse cardiometabolic risk factors. Social differences vary by sex for several risk factors, including higher smoking among men and obesity among women. Public health interventions considering both deprivation and sex are warranted.

**WHAT IS ALREADY KNOWN ON THIS TOPIC**

- Prior observational studies have reported that lower socioeconomic status (SES) is associated with higher prevalence of established cardiometabolic risk factors, such as hypertension and diabetes.
- However, there is limited data on socioeconomic differences in emerging risk factors, such as estimated glomerular filtration rate and C-reactive protein, and the prevalence of cardiometabolic risk factors according to both social deprivation and sex have not been fully explored.

**WHAT THIS STUDY ADDS**

- The social difference varies by sex for several risk factors, including higher rates of smoking among men and obesity among women.
- This study novelly considered both traditional and emerging risk factors and found, for example, higher concentrations of C-reactive protein among those with greater social deprivation.
- Similar trends in cardiometabolic risk factors were observed for area-level, household-level and individual-level indicators of SES, which have not been explored together in prior studies.

**HOW THIS STUDY MIGHT AFFECT RESEARCH, PRACTICE OR POLICY**

- These results highlight the importance of examining cardiovascular risk in the context of both sex and SES.
- From a public health perspective, both sex and SES should be considered to identify high risk groups and develop targeted policy recommendations.

## Introduction

 There is established evidence that higher social deprivation is associated with higher cardiovascular disease (CVD) incidence and mortality in higher-income countries.^1^,^4^ Despite efforts to address CVD inequalities, recent evidence suggests that social gradients in CVD outcomes persist, across different age groups and in both sexes.^5^ Socioeconomic disparities in CVD outcomes have been attributed to differences in cardiometabolic risk factors, such as smoking and obesity, as well as to barriers to acceptance and access to treatment.^6 7^ The recently established Commission on Inequalities and Disparities in Cardiovascular Health by The Lancet Regional Health-Europe underscores the importance of further research on the root causes of CVD disparities to inform evidence-based policy recommendations.^8^

Evidence from prior systematic reviews and meta-analyses of epidemiological studies shows that lower socioeconomic status (SES) is associated with a higher prevalence of established cardiometabolic risk factors, including smoking, hypertension, obesity and diabetes.^1^,^36 9^ However, evidence is less clear for novel and emerging cardiometabolic risk factors, such as estimated glomerular filtration rate (eGFR) and C-reactive protein (CRP). Moreover, observational studies have demonstrated that socioeconomic differences in CVD outcomes are generally stronger in magnitude in women than men,^2^ yet sex differences in cardiometabolic risk factors across the social gradient remain under-researched. An intersectionality-informed perspective suggests that overlapping social positions, including SES and sex, may jointly shape patterns of cardiometabolic risk.^10^ Lastly, most existing studies on this topic only consider a single indicator of SES, such as Townsend Deprivation Index or education, and it is not known how the choice of SES indicator might influence these associations.^7^

We address these gaps in a large UK cohort, investigating socioeconomic differences in the prevalence, treatment and control of cardiometabolic risk factors and whether these vary by sex.

## Methods

### Subjects and study design

The UK Biobank (UKB) is a population-based prospective cohort study of 503 317 UK adults established between 2006 and 2010.^11^ Approximately 9.2 million eligible adults, aged between 40 and 69 years of age and living within 25 miles of 1 of 22 assessment centres in England, Wales and Scotland, were invited to participate. Participants self-reported detailed lifestyle and socio-demographic information were gathered via a touchscreen questionnaire and interview. Anthropometric and biological data, including blood samples, were also taken using standardised procedures. Further details, including the full study protocol and data access for researchers can be found via the UKB website.^11^ This research was conducted using the UKB Resource under Application Number 74018. We acknowledge that sex and gender are related but distinct constructs; however, as UKB collects self-reported sex and does not comprehensively capture gender identity or gender-related constructs, we use the term ‘sex’ throughout to reflect the available data.^12^

### Assessment of socioeconomic status

We considered three indicators of SES measured at baseline: Townsend Deprivation Score (an area-level measure) in our primary analysis, and household income (household-level) and educational attainment (individual-level) in our secondary analysis. Townsend scores were assigned to participants based on their postcode at recruitment using data from the preceding national census (accounting for unemployment, car ownership, household overcrowding and owner occupation).^13^ Participants were categorised into nationally defined Townsend Deprivation Score fifths from least to most deprived using census-based cut points.^14^ These cut-points reflect the national distribution of deprivation and therefore do not result in equal group sizes within the UKB cohort. Household income was self-reported at baseline. Educational attainment was self-reported at baseline and was categorised using the International Standard Classification of Education.^15^ The categorisation and levels for all SES indicators are summarised in table 1.

**Table 1:** Socioeconomic status indicators and category definitions

SES indicator	Level	Description
Townsend Deprivation Score (area-level)	Fifth 1	<−2.938 (least deprived)
	Fifth 2	2.938 to <−1.531
	Fifth 3	−1.531 to <0.170
	Fifth 4	0.170 to <2.448
	Fifth 5	≥2.448 (most deprived)
Household income (household-level), £/year	Group 1	> 100 000 (highest household income)
	Group 2	52 000 to 100 000
	Group 3	31 000 to 51 999
	Group 4	18 000 to 30 999
	Group 5	<18 000 (lowest household income)
Educational attainment	Group 1	College or University degree (highest educational attainment)
	Group 2	Education to ≥18 years (A levels/AS levels; NVQ/HND/HNC or equivalent; other professional qualifications)
	Group 3	Education to ≥16 years (O levels/GCSEs/CSEs or equivalent)
	Group 4	No qualifications (lowest educational attainment)

CSEs, Certificate of Secondary Education; GCSEs, General Certificate of Secondary Education; HNC, Higher National Certificate; HND, Higher National Diploma; O levels, General Certificate of Education Ordinary Levels; A levels, General Certificate of Education Advanced Level; AS levels, General Certificate of Advanced Subsidiary Levels; NVQ, National Vocational Qualification; SES, socioeconomic status.

### Measurement of risk factors

Risk factors were selected a priori based on the prior evidence supporting a relationship between the risk factor and cardiometabolic disease endpoints, as well as their inclusion in cardiovascular risk algorithms.^16^ Risk factors in this study were measured at baseline and included current smoking, systolic blood pressure (SBP), diastolic blood pressure (DBP), body mass index (BMI), waist circumference, obesity, lipids, glycated haemoglobin (HbA1c), glucose, eGFR,^17^ chronic kidney disease (CKD) and CRP, as well as treatment and control of hypertension, dyslipidaemia and diabetes based on UK guidelines.^18 19^ Further information on risk factor definitions is presented in the (online supplemental methods).

### Exclusion criteria

Participants who subsequently withdrew consent (n=1133), did not have a valid baseline measurement of at least one cardiometabolic risk factor (n=795) or were missing the Townsend Deprivation Score (n=602) were excluded from the main analyses (online supplemental figure 1). For secondary analyses of other SES indicators, participants who did not have information on household income (n=55 331) or educational attainment (n=9844) were further excluded. Information on participants with missing data for each cardiometabolic risk factor is provided in online supplemental table 1.

### Statistical analysis

Baseline characteristics of participants by SES were presented as mean (SD) for approximately normally-distributed continuous variables, median (interquartile interval) for approximately non-normally distributed continuous variables or number (percentage) for categorical variables. Spearman correlations between socioeconomic indicators were also calculated.

We modelled each cardiometabolic risk factor as the dependent variable and each SES indicator as the independent variable, and calculated age-adjusted summary estimates with 95% CIs. Age-adjusted least square means were calculated using linear regression and binomial regression (with a log link) for continuous and categorical variables, respectively. For approximately non-normally distributed variables (triglycerides, HbA1c, glucose and CRP), we regressed log-transformed variables and exponentiated the regression coefficients to obtain the geometric means. Normality of these variables was assessed using visual inspection of their distributions, and the distributions of the log-transformed variables were subsequently visually inspected to confirm approximate normality. Formal tests of normality were not used because, in very large samples, they may detect trivial departures from normality. We calculated percentages of treatment and control (control among treated and among overall population) for hypertension, dyslipidaemia and diabetes. Differences between the lowest and highest categories of each socioeconomic indicator were presented as a summary measure of inequality magnitude. We also used binomial regression (with log link) to estimate the age-adjusted prevalence ratios (PRs) for cardiometabolic risk factors between the lowest and highest group for each SES indicator, by sex.

To investigate whether socioeconomic differences in risk factors differed by sex (women, men), we included a two-way interaction term between the SES indicator of interest and sex in the regression models for each outcome. A sensitivity analysis was performed using country-specific fifths of the overall Index of Multiple Deprivation (IMD) score, which is an area-level indicator of SES that considers factors not accounted for in the Townsend score (accounting for between 8 and 9 factors, such as crime and housing, depending on the country).^20^ We also re-weighted the main analyses according to the social structure of the general population using national Townsend fifths, as a sensitivity analysis. Lastly, we repeated the analyses of household income adjusting for self-reported number of people in the household, to account for the differences in financial resources required between households and repeated the main analyses excluding participants taking lipid-lowering medication at baseline.

Analyses were performed using Stata V.18.0 and R V.4.3.3.

## Results

### Baseline characteristics and socioeconomic status indicators

This study included 500 769 UKB participants (54.4% women; mean age 56.5 years). The distribution of participants sampled favoured those of higher SES; for example, 37.2% were from the least deprived fifth compared with 14.2% in the most deprived fifth, according to the Townsend score (table 2). The proportion of women and men within each Townsend score fifth was similar, whereas a higher proportion of women than men were in the lowest income group (24.8% vs 20.7%) (online supplemental table 2). Spearman correlations were low between Townsend deprivation fifths and both household income (r=0.220) and educational attainment (r=0.094), but higher for IMD (r=0.655) (online supplemental figure 2). The distribution of each cardiometabolic risk factor is shown in online supplemental figure 3.

**Table 2:** Participant characteristics by Townsend Deprivation Score fifths

	1 (least deprived)	2	3	4	5 (most deprived)
N	186 076 (37.2%)	101 892 (20.3%)	74 496 (14.9%)	67 267 (13.4%)	71 038 (14.2%)
Women (n (%))	101 108 (54.3)	56 117 (55.1)	41 207 (55.3)	36 865 (54.8)	37 195 (52.4)
Age (mean (SD)), years	57.2 (7.9)	56.9 (8.0)	56.2 (8.2)	55.8 (8.3)	55.2 (8.4)
Townsend Deprivation Score (median (IQI))	−4.0 (−4.6 to −3.5)	−2.3 (−2.6 to −2.0)	−0.8 (−1.2 to 0.3)	1.2 (0.7 to 1.8)	4.2 (3.2 to 5.5)
Household income (n (%)), £/year					
≥100 000	10 429 (6.6)	4223 (4.9)	3193 (5.0)	2857 (5.0)	2173 (3.8)
52 000 to <100 000	40 705 (25.7)	18 478 (21.3)	12 136 (19.0)	8918 (15.6)	5800 (10.0)
31 000 to <52 000	45 644 (28.8)	24 109 (27.8)	16 983 (26.6)	13 640 (23.9)	10 119 (17.5)
18 000 to <31 000	39 280 (24.8)	22 891 (26.4)	16 734 (26.2)	14 741 (25.8)	14 281 (24.7)
<18 000	22 611 (14.3)	16 978 (19.6)	14 767 (23.1)	16 979 (29.7)	25 547 (44.1)
Educational attainment (n (%))					
University/college degree	64 082 (35.0)	31 819 (31.8)	24 469 (33.4)	21 267 (32.3)	19 069 (27.8)
Education to ≥18 years	64 662 (35.3)	34 519 (34.5)	23 584 (32.2)	19 985 (30.4)	19 472 (28.4)
Education to ≥16 years	31 449 (17.2)	17 721 (17.7)	12 347 (16.9)	10 969 (16.7)	10 607 (15.5)
No qualifications	23 122 (12.6)	16 022 (16.0)	12 771 (17.5)	13 596 (20.7)	19 489 (28.4)

Values are means for continuous variables adjusted for age, unless otherwise specified. Values between brackets indicate 95% CIs, unless otherwise specified.

Townsend score fifths: 1 (Townsend score <−2.938, least deprived); 2 (≥−2.938 to <−1.531); 3 (≥−1.531 to <0.170); 4 (≥0.170 to <2.448); and 5 (≥2.448, most deprived).

IQI, interquartile interval.

### Deprivation and cardiometabolic risk factors

**Figure 1:**
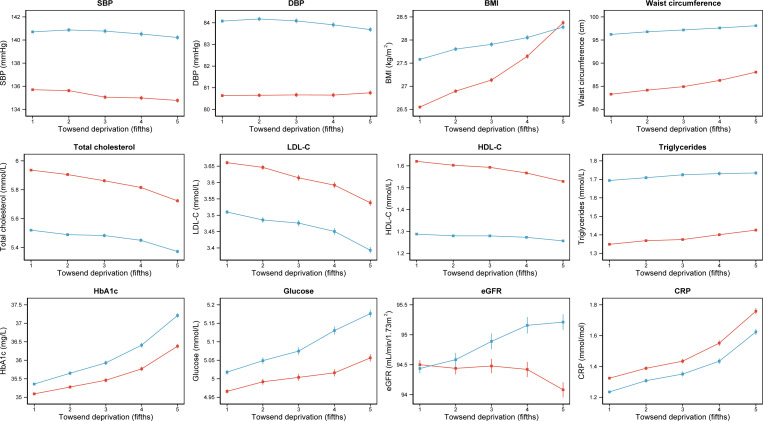
Mean cardiometabolic risk factors, by sex and Townsend deprivation fifths. Values are means (95% CIs) adjusted for age. Red lines are for women, and blue lines are for men. Townsend deprivation fifths: 1 (Townsend deprivation score <−2.938, least deprived); 2 (≥−2.938 to <−1.531); 3 (≥−1.531 to <0.170); 4 (≥0.170 to <2.448); and 5 (≥2.448, most deprived). BMI, body mass index; CRP, C-reactive protein; DBP, diastolic blood pressure; eGFR, estimated glomerular filtration rate; HbA1c, glycated haemoglobin; HDL-C, high-density lipoprotein cholesterol; LDL-C, low-density lipoprotein cholesterol; SBP, systolic blood pressure. Mean values by sex and Townsend deprivation fifths are provided in online supplemental table 4.

Participants’ mean number of risk factors (95% CI) was higher with greater social deprivation, with 26.2% (25.8% to 26.5%) versus 14.5% (14.4% to 14.7%) among the most versus least deprived participants having ≥3 risk factors (online supplemental table 3). CRP was higher with greater deprivation (absolute difference (95% CI) between most and least deprived: 0.42 mg/L (0.40 to 0.43)), whereas total cholesterol, low-density lipoprotein cholesterol (LDL-C), high-density lipoprotein cholesterol (HDL-C) were lower (−0.191 mmol/L (−0.201 to −0.181), −0.123 mmol/L (−0.131 to −0.115) and −0.070 mmol/L (−0.074 to −0.066), respectively). BMI, waist circumference and triglycerides were also higher with greater deprivation, but larger differences were observed among women (+1.8 kg/m^2^ (1.8 to 1.9), +4.8 cm (4.6 to 4.9) and +0.077 mmol/L (0.068 to 0.086), respectively) than men (+0.698 kg/m^2^ (0.638 to 0.758), +1.9 cm (1.7 to 2.0) and +0.040 mmol/L (0.029 to 0.052)) (figure 1, online supplemental table 4). Moreover, average BMI was lower in women than men who were less deprived. HbA1c and glucose were higher in the most deprived groups, but these differences were larger in men (+1.9 mmol/mol (1.8 to 1.9) and +0.159 mmol/L (0.146 to 0.171), respectively) than women (+1.3 mmol/mol (1.2 to 1.4) and +0.090 mmol/L (0.079 to 0.102)). Moreover, eGFR was higher in men with greater deprivation (+0.774 mL/min/1.73 m^2^ (0.620 to 0.929)) but not women (−0.418 mL/min/1.73 m^2^ (−0.564 to −0.271)).

Overall, the prevalence of all risk factors was higher with greater social deprivation. Prevalence of risk factors (95% CI) was higher among men than women across all deprivation groups, except for obesity (33.5% (33.0% to 34.0%) vs 31.1% (30.6% to 31.6%) for the most deprived women vs men, respectively) and dyslipidaemia (88.3% (87.9% to 88.6%) vs 87.1% (86.8% to 87.5%)). Social deprivation was associated with a higher likelihood of current smoking, diabetes, obesity, CKD and hypertension in both sexes, as well as dyslipidaemia in women (PR (95% CI) 1.10 (1.06 to 1.15)) but not men (0.95 (0.91 to 0.99)) (online supplemental figure 4). Moreover, the associations for current smoking, diabetes and obesity were stronger in women (3.68 (3.54 to 3.82), 2.81 (2.61 to 3.04) and 2.05 (2.00 to 2.11), respectively) than men (3.51 (3.39 to 3.64), 2.32 (2.18 to 2.46) and 1.51 (1.47 to 1.55)).

**Figure 2:**
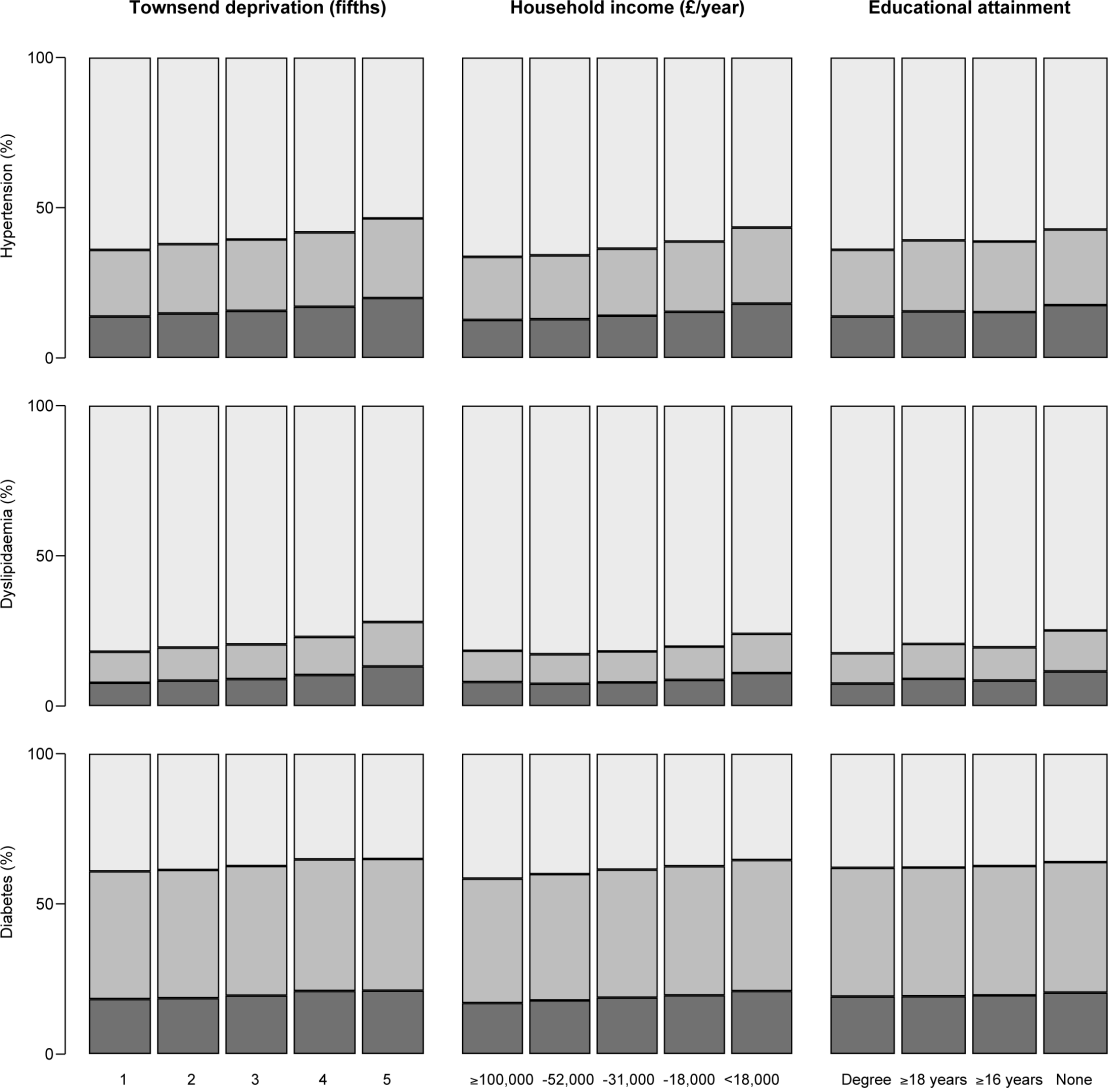
Treatment and control of hypertension, dyslipidaemia and diabetes, by sex and socioeconomic status. Values are percentages adjusted for age. Light grey areas represent untreated cases; medium grey, treated but uncontrolled cases; and dark grey areas, treated and controlled cases. Townsend deprivation fifths: 1 (Townsend deprivation score <−2.938, least deprived); 2 (≥−2.938 to <−1.531); 3 (≥−1.531 to <0.170); 4 (≥0.170 to <2.448); and 5 (≥2.448, most deprived). Household income groups: >£100 000, £52 000 to <£100 000, £31 000 to <£52 000, £18 000 to <£31 000 and <£18 000 per year. Educational attainment groups: degree (university/college degree); ≥18 years (A/AS levels, NVQ/HND/HNC or equivalent, other professional qualifications); ≥16 years (O levels/GCSEs or equivalent, CSEs or equivalent); and none (no qualifications). Percentage values by sex and socioeconomic status are provided in online supplemental tables 4, 6 and 8. A levels, General Certificate of Education Advanced Level; AS levels, General Certificate of Advanced Subsidiary Levels; CSE, Certificate of Secondary Education; GCSE, General Certificate of Secondary Education; HNC, Higher National Certificate; HND, Higher National Diploma; NVQ, National Vocational Qualification; O levels, General Certificate of Education Ordinary Levels.

Treatment of hypertension and dyslipidaemia, as well as being controlled among treated, were higher with greater deprivation, while no differences in diabetes treatment and control were observed (figure 2). Absolute differences in risk factor treatment and control by social deprivation did not vary by sex, although women generally had lower treatment and control rates for dyslipidaemia (online supplemental table 4).

### Household income and cardiometabolic risk factors

**Figure 3:**
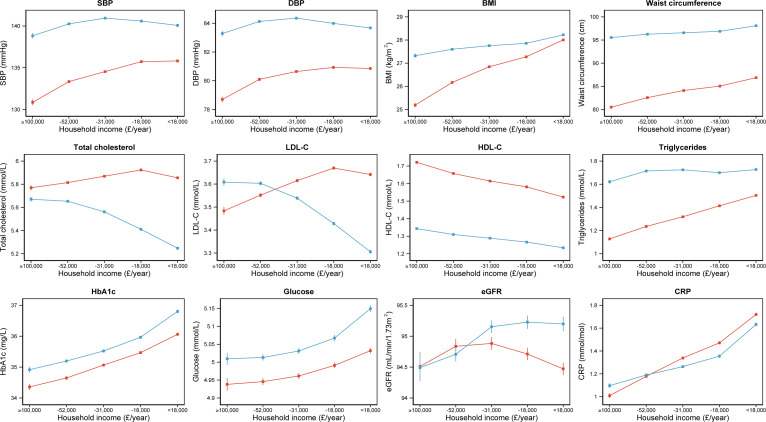
Mean cardiometabolic risk factors, by sex and household income. Values are means (95% CIs) adjusted for age. Red lines are for women, and blue lines are for men. Household income groups: ≥£100 000; £52 000 to <£100 000, £31 000 to <£52 000, £18 000 to <£31 000 and <£18 000 per year. BMI, body mass index; CRP, C-reactive protein; DBP, diastolic blood pressure; eGFR, estimated glomerular filtration rate; HbA1c, glycated haemoglobin; HDL-C, high-density lipoprotein cholesterol; LDL-C, low-density lipoprotein cholesterol; SBP, systolic blood pressure. Mean values by sex and household income are provided in online supplemental table 6.

Cardiometabolic risk factors by household income and sex are shown in figure 3 (values shown in online supplemental tables 5 and 6). SBP and DBP were higher among those with lower household incomes, but with larger differences observed among women (absolute difference (95% CI) between lowest and highest household income: +5.0 mm Hg (4.6 to 5.3) and +2.2 mm Hg (1.9 to 2.4), respectively) than men (+1.3 mm Hg (0.878 to 1.61) and +0.389 mm Hg (0.178 to 0.600)). Moreover, differences in total cholesterol and LDL-C were higher among women with lower household incomes (+0.086 mmol/L (0.062 to 0.110) and +0.158 mmol/L (0.140 to 0.177), respectively), but lower among men (−0.423 mmol/L (−0.446 to −0.400) and −0.303 mmol/L (−0.321 to −0.285)), with LDL-C concentrations in women exceeding those for men when household income was ≤£55 000. The magnitude of the PRs for risk factors comparing between lowest and highest income were larger in magnitude to those comparing between the most and least deprived Townsend fifths, including in subgroup analyses by sex (online supplemental figure 5).

### Educational attainment and cardiometabolic risk factors

**Figure 4:**
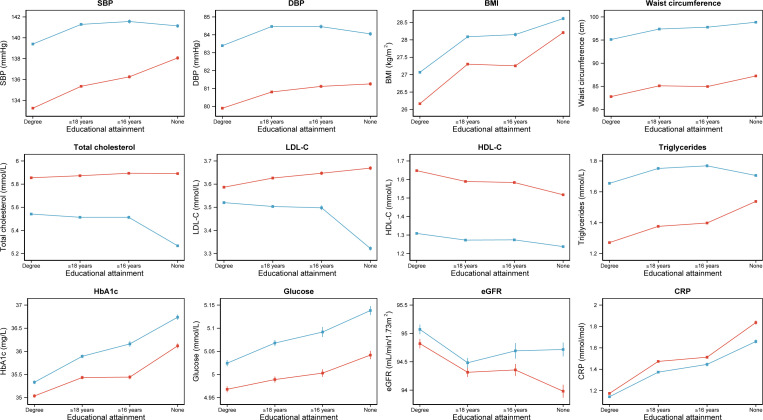
Mean cardiometabolic risk factors, by sex and educational attainment. Values are means (95% CIs) adjusted for age. Red lines are for women, and blue lines are for men. Educational attainment groups: degree (university/college degree); ≥18 years (A/AS levels, NVQ/HND/HNC or equivalent, other professional qualifications); ≥16 years (O levels/GCSEs or equivalent, CSEs or equivalent); and none (no qualifications). A levels, General Certificate of Education Advanced Level; AS levels, General Certificate of Advanced Subsidiary Levels; BMI, body mass index; CRP, C-reactive protein; CSE, Certificate of Secondary Education; DBP, diastolic blood pressure; eGFR, estimated glomerular filtration rate; GCSE, General Certificate of Secondary Education; HbA1c, glycated haemoglobin; HDL-C, high-density lipoprotein cholesterol; HNC, Higher National Certificate; HND, Higher National Diploma; LDL-C, low-density lipoprotein cholesterol; NVQ, National Vocational Qualification; O levels, General Certificate of Education Ordinary Levels; SBP, systolic blood pressure. Mean values by sex and educational attainment are provided in online supplemental table 8.

Figure 4 shows cardiometabolic risk factors by educational attainment and sex (values shown in online supplemental tables 7 and 8). SBP and DBP were higher among those with lower educational attainment, with larger differences observed in women (difference (95% CI) between lowest and highest educational attainment: +4.8 mm Hg (4.6 to 5.0) and +1.4 mm Hg (1.2 to 1.5), respectively) than men (+1.7 mm Hg (1.5 to 2.0) and +0.659 mm Hg (0.530 to 0.788)). Total cholesterol and LDL-C were lower in men with lower educational attainment (−0.275 mmol/L (−0.289 to −0.260) and −0.198 mmol/L (−0.209 to −0.187), respectively), but not women (+0.036 mmol/L (0.023 to 0.049) and +0.083 mmol/L (0.072 to 0.093)), while eGFR was lower with lower educational attainment in both women (−0.839 mL/min/1.73 m^2^ (−0.981 to −0.697)) and men (−0.354 mL/min/1.73 m^2^ (−0.504 to −0.205)). Lower educational attainment was associated with a higher likelihood of dyslipidaemia in both women (PR (95% CI) 1.41 (1.34 to 1.48)) and men (1.15 (1.11 to 1.20)), and higher PRs for obesity were observed in men (2.11 (2.05 to 2.17)) vs women (2.01 (1.96 to 2.07)) (online supplemental figure 6).

### Sensitivity analyses

In sensitivity analyses examining cardiometabolic risk factors by IMD, findings remained similar to the main analyses by Townsend deprivation fifths (online supplemental tables 9 and 10, and online supplemental figures 7 and 8). After the analyses were weighted by Townsend score fifths, results were close to those for the middle Townsend score fifth in the main analyses (online supplemental table 11). The findings for cardiometabolic risk factors by household income and sex did not change substantially after further adjustment for number of people living in the household (online supplemental tables 12 and 13, and online supplemental figures 9 and 10). After excluding participants taking lipid lowering medication, results were similar to the main analyses, although the absolute differences in total cholesterol and LDL-C between most and least deprived were smaller (−0.087 (−0.097 to −0.76) and −0.042 (−0.050 to -0.034), respectively) (online supplemental tables 14 and 15, and online supplemental figures 11 and 12).

## Discussion

This large cross-sectional study of the UK population demonstrates socioeconomic inequalities in cardiometabolic risk factors. Apart from total cholesterol and LDL-C, lower SES relates to worse cardiometabolic risk factors. Socioeconomic differences in certain risk factors varied by sex, with larger disparities in smoking, obesity and diabetes observed among women; however, the prevalence of smoking and diabetes remained highest among the most deprived men. Socioeconomic differences in treatment and control were minimal and, if anything, were higher for hypertension and dyslipidaemia among participants with lower SES. Overall, similar trends were observed for area-level, household-level and individual-level indicators of SES. While prior studies have considered socioeconomic differences in cardiometabolic risk factors,^21 22^ this is the first study to examine the influence of different SES indicators and to explore a comprehensive range of established and novel risk factors, as well as the role of sex.

Cardiometabolic risk factors, including current smoking, hypertension, obesity and diabetes, were generally worse among those with higher social deprivation, measured by area-level indices (Townsend score and IMD), which is consistent with prior systematic reviews and meta-analyses.^1^,^3^ Prevalence of CVD risk factors in relation to SES have been studied in recent cross-sectional population surveys in USA,^22 23^ Canada^24^ and UK.^21^ The direction of socioeconomic differences in risk factors in the UKB were overall similar to those reported for adults aged 19–64 years in Health Survey for England (HSE 2021,^25^ including: current smoking (difference between least and most deprived IMD score: +12% vs +13%, respectively); hypertension (+8% vs +17%); obesity (+14% vs +14%); and diabetes (+3% vs +10%). While the prevalence of hypertension was higher with increasing deprivation in UKB, the difference was smaller than that in HSE 2021 and differences in SBP and DBP were moderate (<1 mm Hg), whereas wider differences in BP across SES groups have been observed in previous studies.^21 22^ The magnitude of socioeconomic differences in diabetes, HbA1c and glucose were also smaller in UKB than prior studies in high-income countries.^26^ UKB participants are typically healthier and less socioeconomically deprived than the general UK population,^27^ which may partly account for the differences in the absolute values of certain risk factors (eg, lower BMI and HbA1c) found in UKB when compared with HSE, which is a nationally-representative sample.^25^ As evidence, in the present study, absolute values of adiposity and glycaemic markers became closer to those reported in the 2021 HSE^25^ after weighting for Townsend deprivation, and relative differences across socioeconomic groups were closer.

While the prevalence of dyslipidaemia did not vary greatly by social deprivation, total cholesterol, LDL-C and HDL-C were lower with higher deprivation in UKB. Similarly, raised total cholesterol was less prevalent among more deprived adults in the HSE 2021.^25^ While the existing literature on this topic is mixed, a positive association of social deprivation with total cholesterol and LDL-C has been observed more often in studies in high-income countries, such as our own.^9 28^ Notably, lipid-lowering medication use was higher among the participants in the most (23%) versus least (16%) socially deprived fifth, as assigned based on postcode. These treatment differences might be explained by the inclusion of postcode in the QRISK cardiovascular risk algorithm,^16^ which guides the initiation of lipid-lowering therapy in most of the UK. This pattern (lower total cholesterol and LDL-C with greater social deprivation) persisted after excluding participants taking lipid-lowering medication, although the magnitudes of differences were smaller. Moreover, we novelly examined CRP, finding higher concentrations among those with greater social deprivation, which is consistent with a limited number of prior observational studies.^29^

While lower SES is associated with higher risk of CVD in both sexes, this association is more consistent and stronger among women than men.^2 3^ This may be explained by differences in cardiometabolic risk factors across the social gradient. Socioeconomic disparities in adiposity were wider among women, with a higher obesity PR observed in women than men, which is comparable with adults in HSE 2021.^25^ These findings are consistent with prior epidemiological evidence demonstrating that the inverse relationship between obesity and SES is stronger among women than men in high-income countries.^30 31^ Similarly, we observed greater relative differences across SES groups for current smoking and diabetes. Yet, the prevalence of current smoking and diabetes remained higher in men across all SES groups, suggesting reducing smoking and diabetes rates among lower SES groups should be prioritised in both sexes. Lastly, socioeconomic differences in CKD did not vary by sex, but we observed contrasting socioeconomic differences in eGFR for men (higher with lower SES) and women (lower with lower SES). However, these differences in eGFR were small (<0.5 mL/min/1.73 m^2^), and are unlikely to be clinically meaningful.^17 32^ The observed sex differences in socioeconomic patterning of cardiometabolic risk factors may therefore reflect gendered differences in health behaviours, caregiving roles, occupational exposures, health-seeking patterns and structural disadvantage across the social gradient, rather than biological differences alone.^33 34^

Four measures of SES have been consistently related with CVD in observational studies in high-income countries, including neighbourhood deprivation, income, employment status and education.^1^,^4^ Until now, no prior studies have considered multiple indicators of SES in relation to individual cardiometabolic risk factors in the UK. Overall, we observed similar trends in cardiometabolic risk by deprivation, household income and educational attainment; however, the magnitude of socioeconomic differences varied for different SES indicators. This was most notable for BP and adiposity, where socioeconomic differences in the prevalence of hypertension and obesity were larger for household income and educational attainment than for social deprivation, especially among women. Differing from deprivation, total cholesterol and LDL-C were higher among women with lower household income or educational attainment. A recent meta-analysis of observational studies found that the influence of income and education on CVD incidence is greater in women than men.^3^

There are several potential explanations for the observed differences in cardiometabolic risk factors across the socioeconomic gradient, as well as by sex. First, access to risk factor management and care, as well as acceptance and compliance with treatments, tends to be higher among individuals with higher income and educational levels.^6 35 36^ However, risk factor treatment and control did not vary greatly according to any of the SES indicators examined in the present study. Second, disparities may be related to other psychosocial factors, such as higher rates of psychological distress and lack of social or material resources to manage stressful events, as well as adverse health behaviours, including smoking, physical inactivity, poor diet and alcohol use.^37 38^ We observed the widest socioeconomic disparities for current smoking, which was the only behavioural risk factor studied.

The strengths of the current study include the large cohort size and a comprehensive analysis of SES, considering aspects of neighbourhood deprivation, household income and educational attainment. Moreover, a wide range of established and emerging risk factors were examined, including eGFR and CRP, which have not been considered in similar previous studies.^21^,^24^ This study also has some limitations. First, household income, education and risk factor treatment and control were self-reported and may be prone to bias. Second, the effect of household income can be expected to differ by household size and composition. Although results remained similar in sensitivity analyses adjusting for the number of people living in the participants’ household, this does not allow for other factors. Third, as models were adjusted for age only, other factors such as ethnicity, lifestyle characteristics or medication use may have influenced the observed differences; however, our primary aim was to quantify socioeconomic patterns rather than estimate fully adjusted effects. Fourth, UKB participants are of mostly white European ancestry and are typically healthier and more socioeconomically advantaged,^27^ which may limit the generalisability of our findings. Nevertheless, socioeconomic differences were broadly comparable with HSE 2021,^25^ with sensitivity analyses weighted by Townsend score showing moderate differences for most risk factors. Lastly, the relationship between SES and CVD may be bidirectional, with strong evidence that SES influences CVD outcomes, as well as evidence that CVD outcomes influence SES.^3^ Given the cross-sectional design of the present study, it was not possible to assess the direction of SES-risk factor associations.

## Conclusion

Despite UKB participants being generally healthier and less deprived than the general UK population, large socioeconomic disparities in cardiometabolic risk factors were still observed. Cardiometabolic risk factors, except for total cholesterol and LDL-C, were generally worse with higher social deprivation, lower household income and lower educational attainment. The socioeconomic differences varied by sex for several risk factors, with especially high rates of smoking among men and obesity among women. Our results highlight the importance of examining cardiovascular risk in the context of both SES and sex. From a public health perspective, both sex and SES should be considered to identify higher risk groups and develop targeted policy recommendations.

## Supplementary material

10.1136/bmjph-2025-002961 online supplemental file 1

## Data Availability

Data are available upon reasonable request.
