# Supplementary material for: Socioeconomic differences in the prevalence, treatment and control of major cardiometabolic risk factors by sex: a cross-sectional study of the UK Biobank
Source: BMJ Public Health. 2026 Jun 15;4(2):e002961. doi: 10.1136/bmjph-2025-002961 (PMC13289021; doi:10.1136/bmjph-2025-002961)
Supplement: online supplemental file 1 [file bmjph-4-2-s001.pdf]

SUPPLEMENTARY MATERIALS

Socioeconomic differences in the prevalence, treatment and control of major cardiometabolic risk factors by sex: a cross-sectional study of the UK Biobank

Rebecca K Kelly, Katie Harris, Paul Muntner, Mark Woodward

SUPPLEMENTAL METHODS..4

SUPPLEMENTAL FIGURES..6

Supplemental Figure 1. Flow chart of participants included in the current study......6

Supplemental Figure 2. Spearman correlations between indicators of socioeconomic status. 7

Supplemental Figure 3. Kernel density distributions for cardiometabolic risk factors at baseline 8

Supplemental Figure 4. Prevalence ratios for cardiometabolic risk factors in the most versus least Townsend deprivation fifth, by sex. 10

Supplemental Figure 5. Prevalence ratios for cardiometabolic risk factors in the lowest versus highest household income group, by sex. 11

Supplemental Figure 6. Prevalence ratios for cardiometabolic risk factors in the lowest versus highest educational attainment group, by sex. 12

Supplemental Figure 7. Mean cardiometabolic risk factors, by Index of Multiple Deprivation fifths and sex. 13

Supplemental Figure 8. Prevalence ratios for cardiometabolic risk factors in the most versus least deprived Index of Multiple Deprivation fifth, by sex. 15

Supplemental Figure 9. Mean cardiometabolic risk factors, by household income and sex, with further adjustment for number of people living in household. 16

Supplemental Figure 10. Prevalence ratios for cardiometabolic risk factors in the lowest versus highest household income group, by sex, with further adjustment for number of people living in household..18

Supplemental Figure 11. Mean cardiometabolic risk factors, by sex and Townsend deprivation fifths, after excluding participants taking lipid-lowering medication. 19

Supplemental Figure 12. Prevalence ratios for cardiometabolic risk factors in the most versus least Townsend deprivation fifth, by sex, after excluding participants taking lipid-lowering medication. 21

SUPPLEMENTAL TABLES..22

Supplemental Table 1. Number of participants who contributed to the baseline and follow-up analyses of each cardiometabolic risk factor, by Townsend Deprivation Score fifths. 22

Supplemental Table 2. Participant characteristics by sex. 23

Supplemental Table 3. Cardiometabolic risk factors, treatment, and control, by Townsend Deprivation Score fifths. 24

Supplemental Table 4. Cardiometabolic risk factors, treatment, and control, by Townsend Deprivation Score fifths and sex. 26

Supplemental Table 5. Cardiometabolic risk factors, treatment, and control, by household income. 29

Supplemental Table 6. Cardiometabolic risk factors, treatment, and control, by household income and sex. 31

Supplemental Table 7. Cardiometabolic risk factors, treatment, and control, by educational attainment. 34
Supplemental Table 8. Cardiometabolic risk factors, treatment, and control, by educational attainment and sex. 36
Supplemental Table 9. Cardiometabolic risk factors, treatment, and control, by Index of Multiple Deprivation fifths. 39
Supplemental Table 10. Cardiometabolic risk factors, treatment, and control, by Index of Multiple Deprivation fifths and sex. 41
Supplemental Table 11. Cardiometabolic risk factors, treatment, and control, weighted by National Townsend Deprivation Score fifths. 44
Supplemental Table 12. Cardiometabolic risk factors, treatment, and control, by household income, with further adjustment for number of people living in household. 45
Supplemental Table 13. Cardiometabolic risk factors, treatment, and control, by household income and sex, with further adjustment for number of people living in household. 47
Supplemental Table 14. Cardiometabolic risk factors, treatment, and control, by Townsend Deprivation Score fifths, after excluding participants taking lipid-lowering medication. 50
Supplemental Table 15. Cardiometabolic risk factors, treatment, and control, by Townsend Deprivation Score fifths and sex, after excluding participants taking lipid-lowering medication. 52
REFERENCES 55

SUPPLEMENTAL METHODS

Blood pressure

Systolic blood pressure (SBP) and diastolic blood pressure (DBP) were calculated from the mean of two automated readings taken seated using the Omron HEM-7015IT electronic BP monitor.

Anthropometry

Body mass index (BMI) was calculated as weight/height^2 (kg/m^2), using weight measured using the Tanita BC-418 body composition analyser and standing height measured using a Seca 202 stadiometer. Waist circumference in centimetres (cm) was measured with a Wessex non-stretchable sprung tape.

Biomarkers

Non-fasting venous blood samples were used to measure biomarker concentrations: including, serum lipids (total cholesterol, LDL-C, HDL-C, triglycerides, glycated haemoglobin (HbA1c), glucose, creatinine, and C-reactive protein (CRP). Estimated glomerular filtration rate (eGFR) was calculated from serum creatinine using the 2021 Chronic Kidney Disease Epidemiology Collaboration (CKD-EPI) creatinine equation.[1]

Current smoking

Smoking status was self-reported.

Hypertension

Hypertension was defined from self-report (doctor-diagnosed hypertension or antihypertensive medication use) or BP measurement (SBP ≥140 mmHg or DBP ≥90 mmHg).[2]

Obesity

Obesity was defined as a BMI ≥30 kg/m^2.

Dyslipidaemia

Dyslipidaemia was defined from self-report (lipid-lowering medication use) or serum lipids (total cholesterol ≥5 mmol/L or non-HDL-C ≥4 mmol/L or HDL-C ≤1·2 in women or HDL-C ≤1 mmol/L in men).[3]

Diabetes

Diabetes was defined from self-report (doctor diagnosed diabetes or glucose-lowering medication use) or HbA1c (≥48 mmol/mol [6·5%]).[4]

Chronic kidney disease

CKD was defined by self-report (doctor-diagnosed renal/kidney failure) or eGFR (<60 mL/min/1·73m^2 or self-reported CKD.[1]

Risk factor control

Risk factor control, among those treated, was defined as: SBP <130 mmHg and DBP <80 mmHg for hypertension; cholesterol <5 mmol/L and non-HDL-C <4 mmol/L and HDL-C >1·2 mmol/ or >1 mmol/L in women and men, respectively, for dyslipidaemia; and HbA1c <48 mmol/mol for diabetes.[2-4]

SUPPLEMENTAL FIGURES

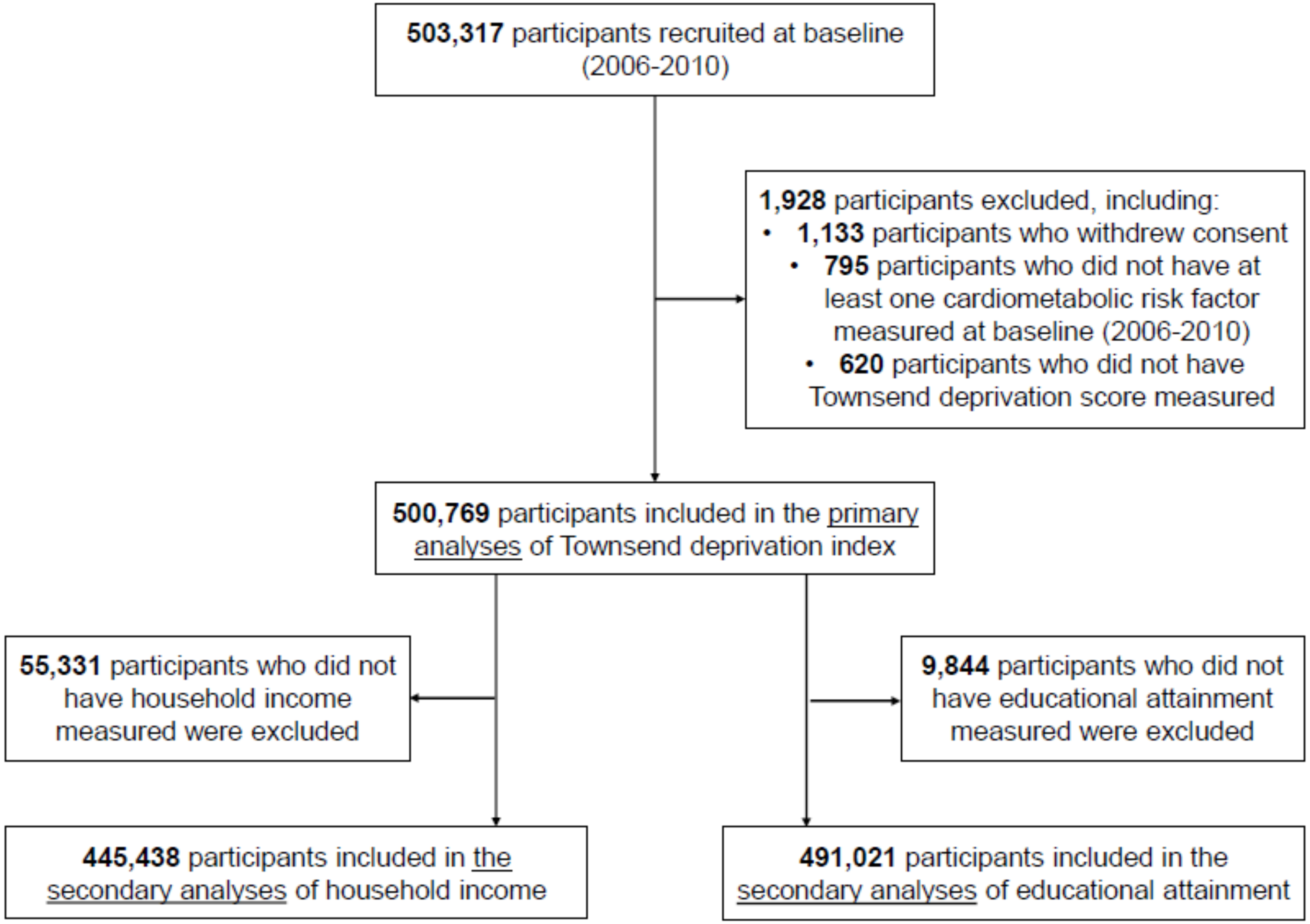

Supplemental Figure 1. Flow chart of participants included in the current study.

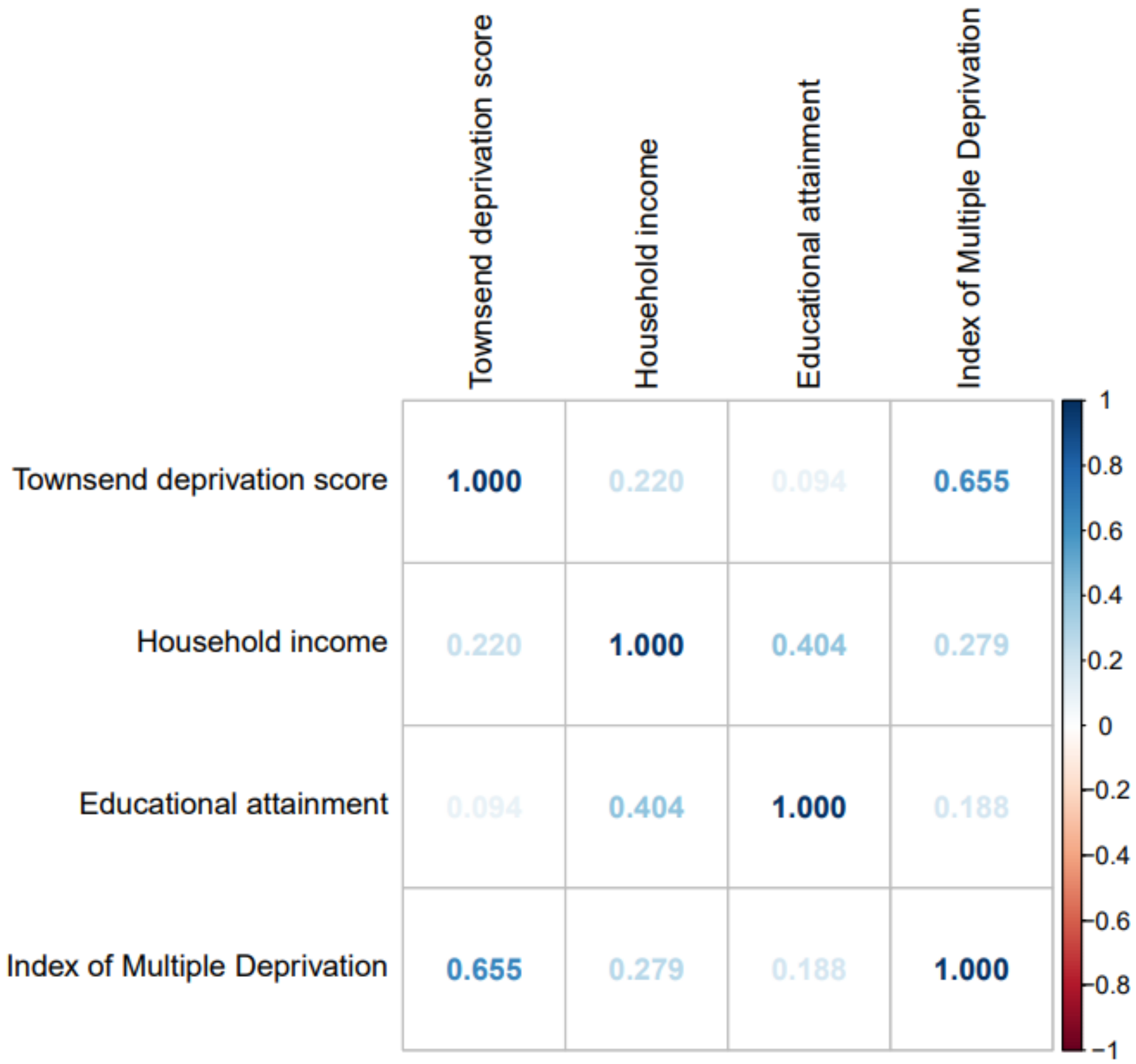

Supplemental Figure 2. Spearman correlations between indicators of socioeconomic status.

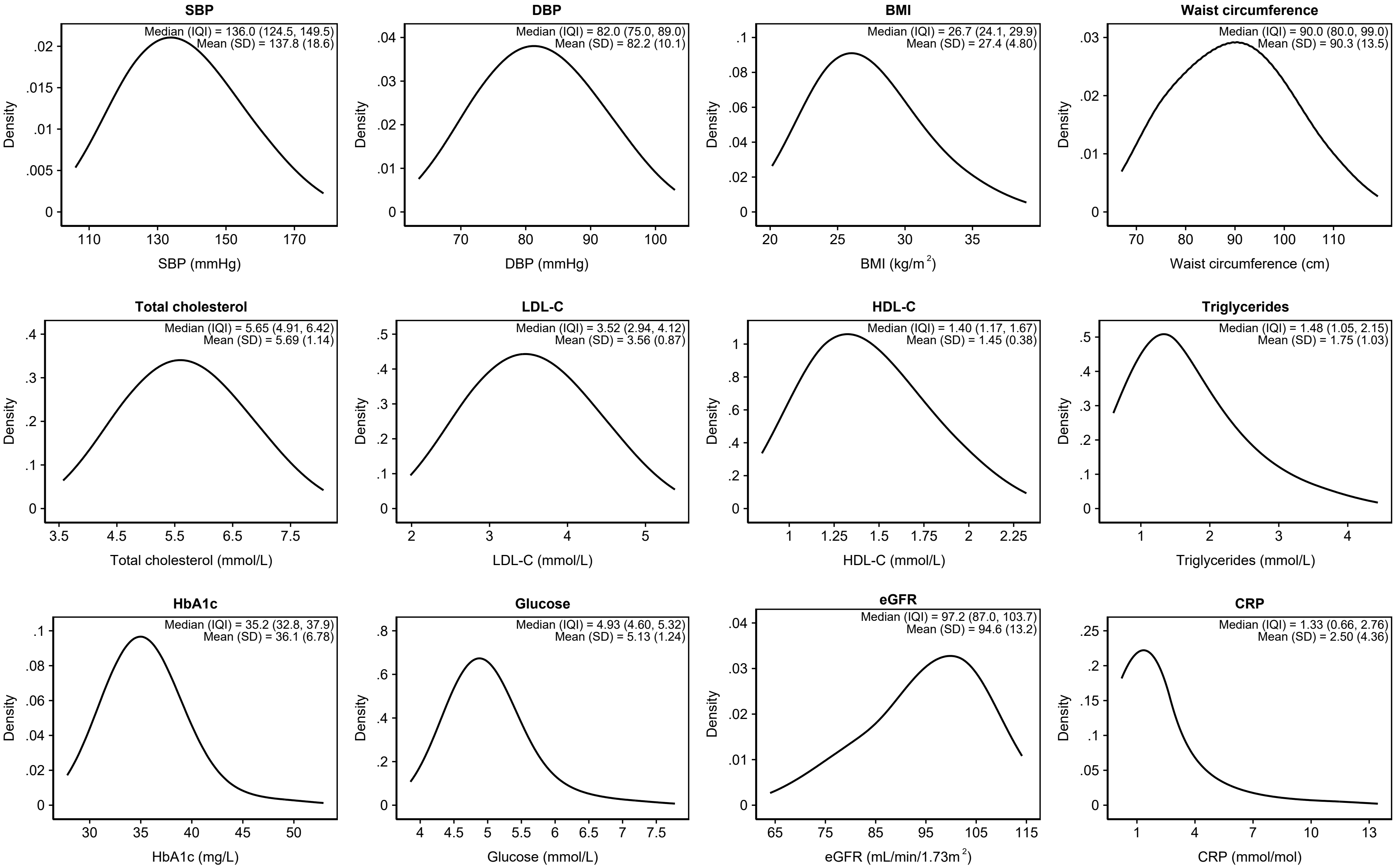

Supplemental Figure 3. Kernel density distributions for cardiometabolic risk factors at baseline

Plots exclude values from the top and bottom 2.5% of each distribution for visualisation only; all observations were retained in analyses. Mean (SD) and median (IQR) values are also shown for each risk factor. BMI, body mass index; CRP, C reactive protein; DBP, diastolic blood pressure; eGFR, estimated glomerular filtration rate; HbA1c, glycated haemoglobin; HDL-C, high-density lipoprotein cholesterol; LDL-C, low-density lipoprotein cholesterol; SBP, systolic blood pressure.

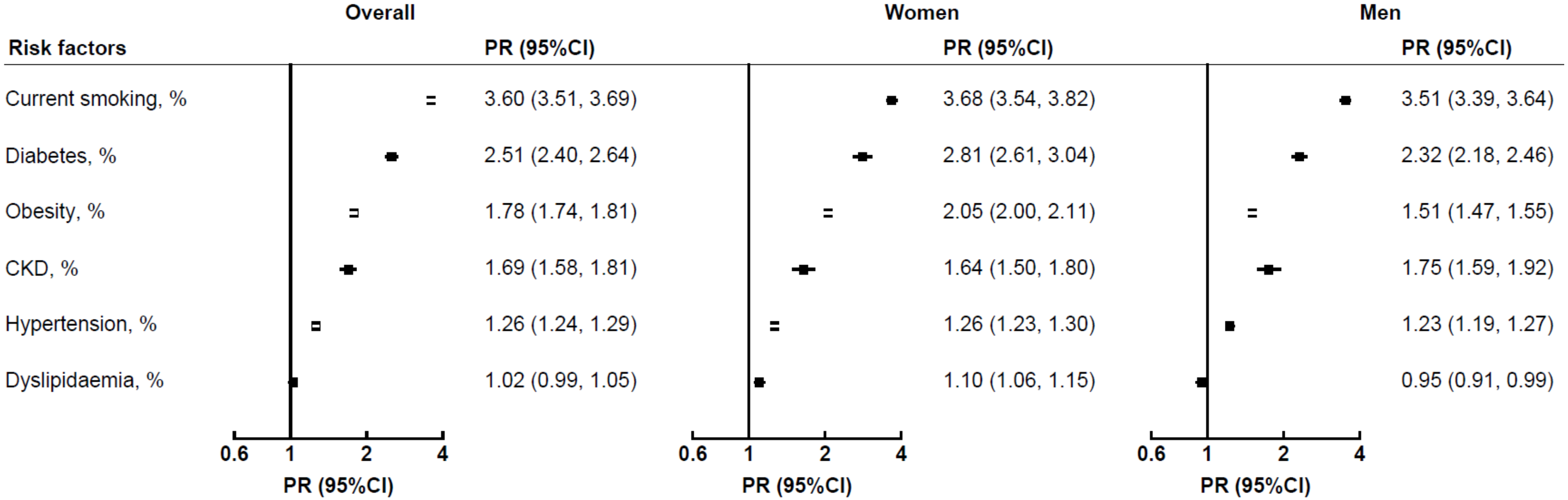

Supplemental Figure 4. Prevalence ratios for cardiometabolic risk factors in the most versus least Townsend deprivation fifth, by sex.

Values are prevalence ratios (95% confidence intervals) adjusted for age. CI = confidence interval; CKD = chronic kidney disease; PR = prevalence ratio.

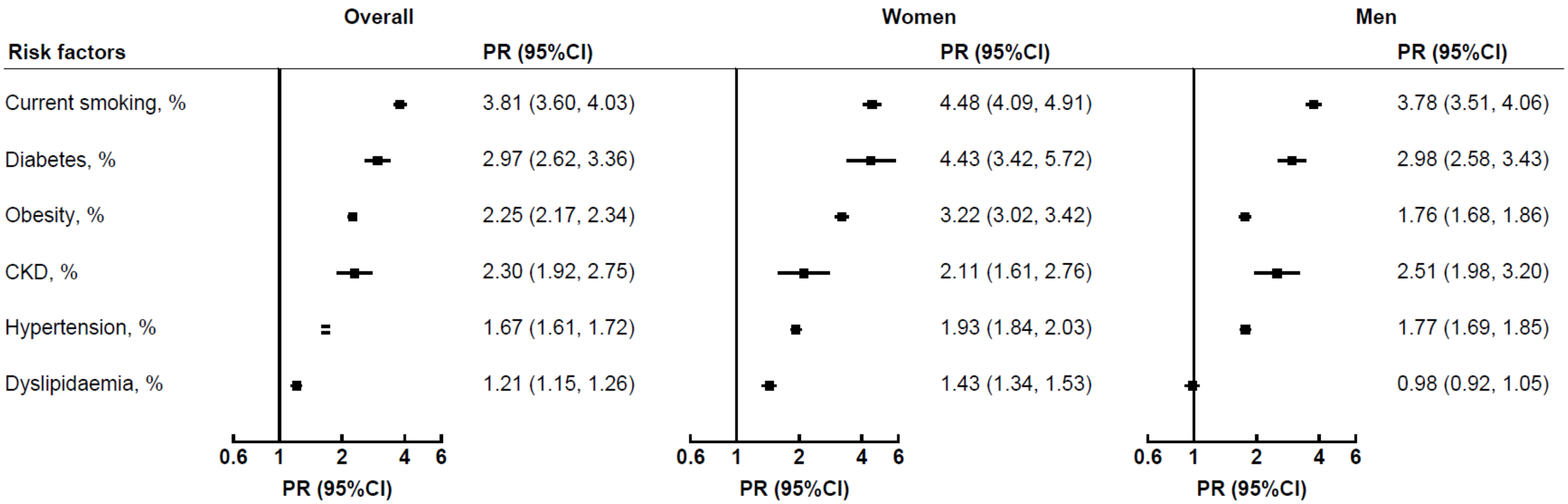

Supplemental Figure 5. Prevalence ratios for cardiometabolic risk factors in the lowest versus highest household income group, by sex.

Values are prevalence ratios (95% confidence intervals) adjusted for age. CI = confidence interval; CKD = chronic kidney disease; PR = prevalence ratio.

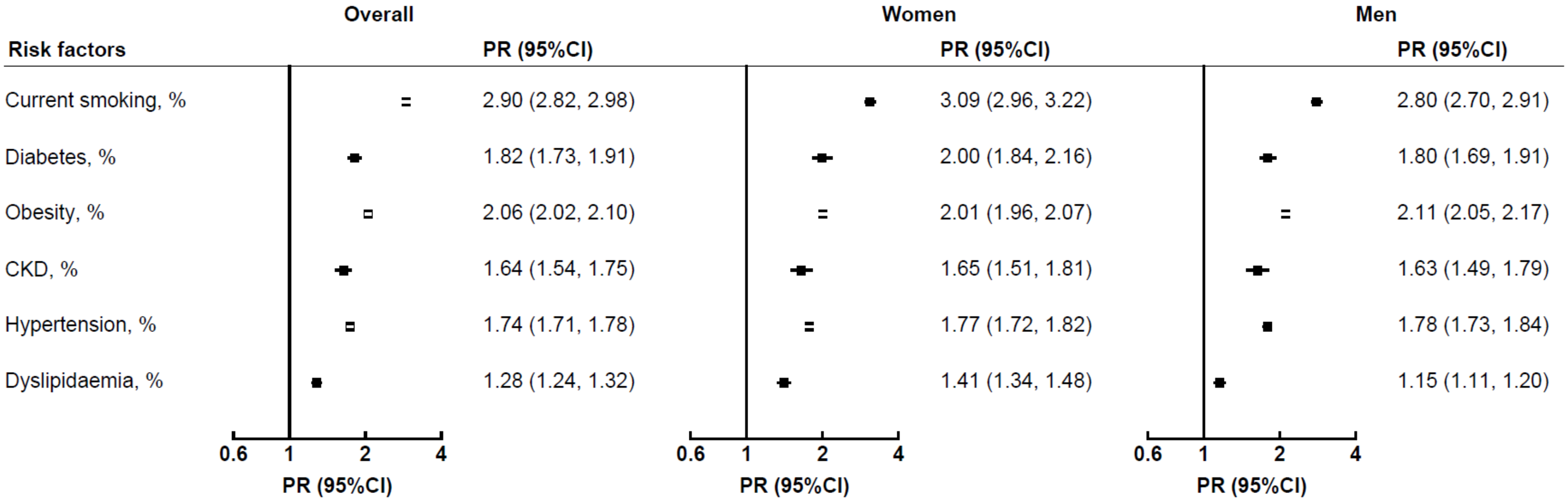

Supplemental Figure 6. Prevalence ratios for cardiometabolic risk factors in the lowest versus highest educational attainment group, by sex.

Values are prevalence ratios (95% confidence intervals) adjusted for age. CI = confidence interval; CKD = chronic kidney disease; PR = prevalence ratio.

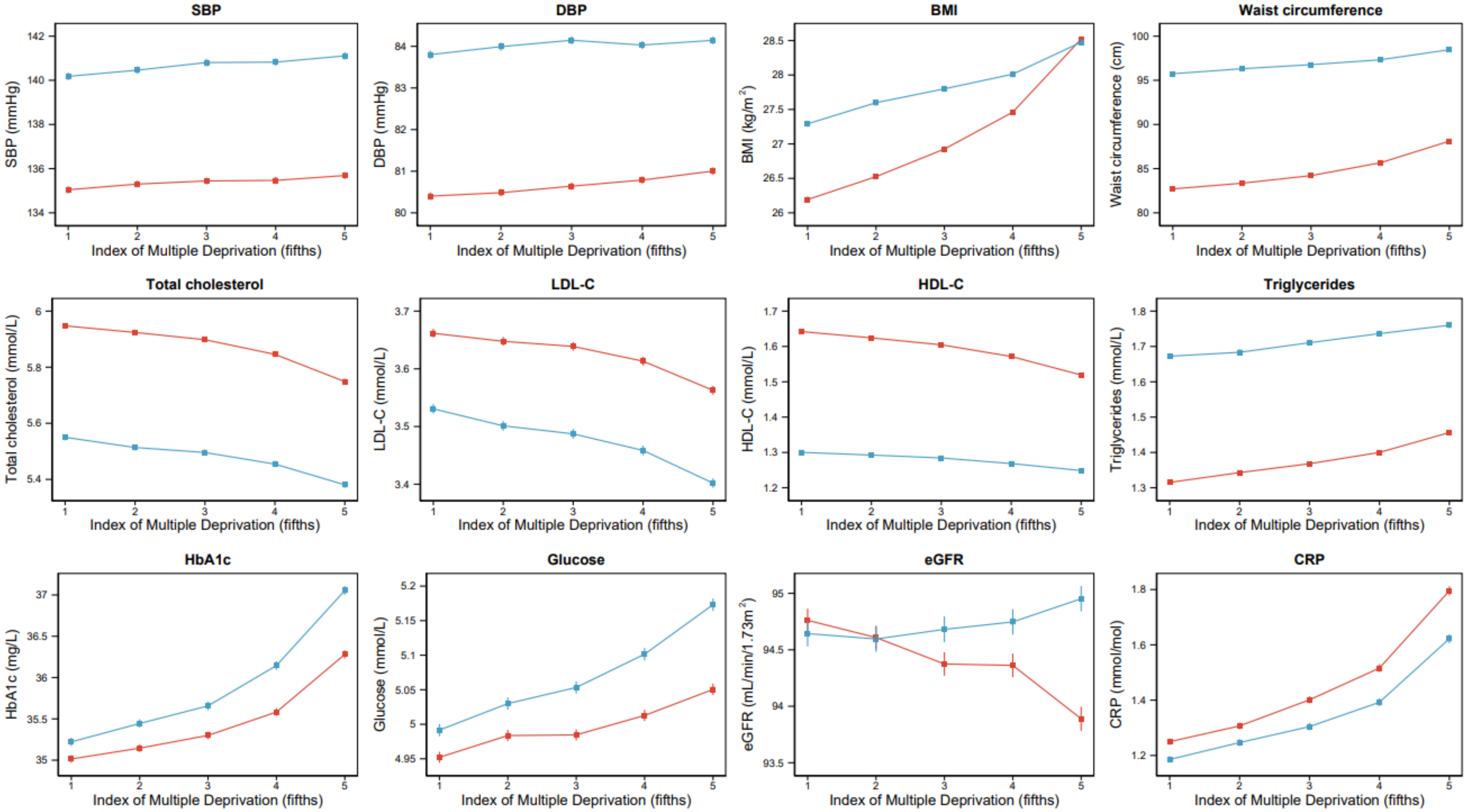

Supplemental Figure 7. Mean cardiometabolic risk factors, <u>by Index of Multiple Deprivation fifths and sex</u>.

Values are means (95% confidence intervals) adjusted for age. Red lines are for women, and blue lines are for men. Index of Multiple Deprivation fifths: ranging from 1 (least deprived) to 5 (most deprived). Mean values by sex and Index of Multiple

Deprivation fifths are provided in Table S5. BMI = body mass index; CRP = C-reactive protein; DBP = diastolic blood pressure; eGFR = estimated glomerular filtration rate; HbA1c = glycated haemoglobin; HDL-C = high-density lipoprotein cholesterol; LDL-C = low-density lipoprotein cholesterol; SBP = systolic blood pressure. Mean values by sex and educational attainment are provided in online supplemental table 10.

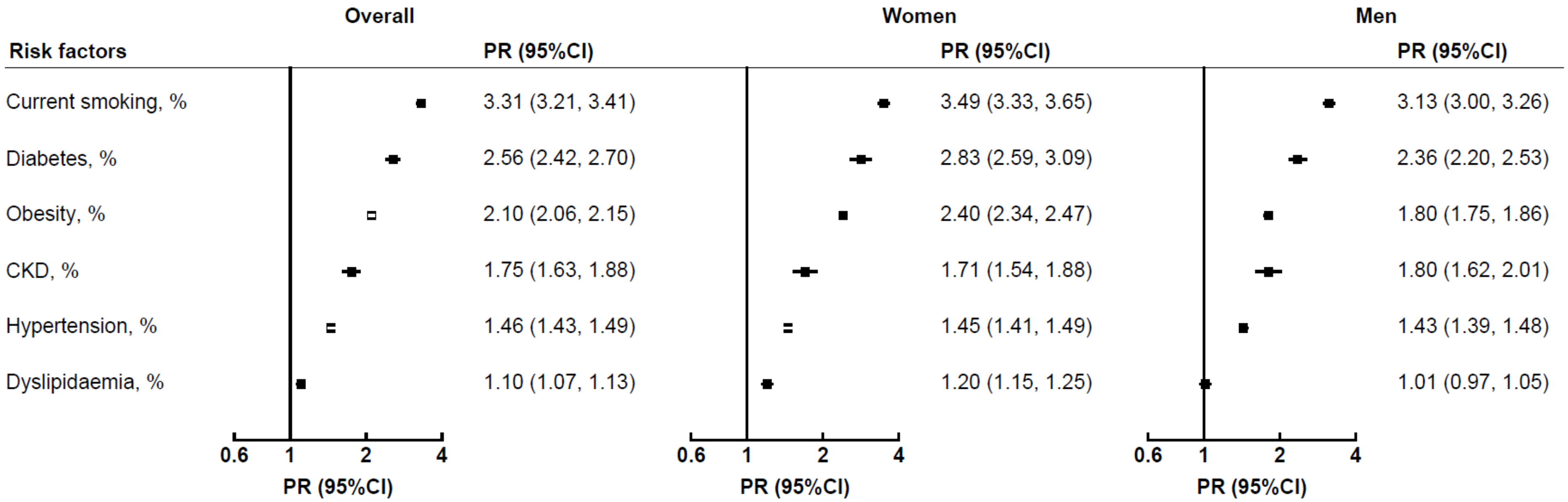

Supplemental Figure 8. Prevalence ratios for cardiometabolic risk factors in the most versus least deprived Index of Multiple Deprivation fifth, by sex.

Values are prevalence ratios (95% confidence intervals) adjusted for age. CI = confidence interval; CKD = chronic kidney disease; PR = prevalence ratio.

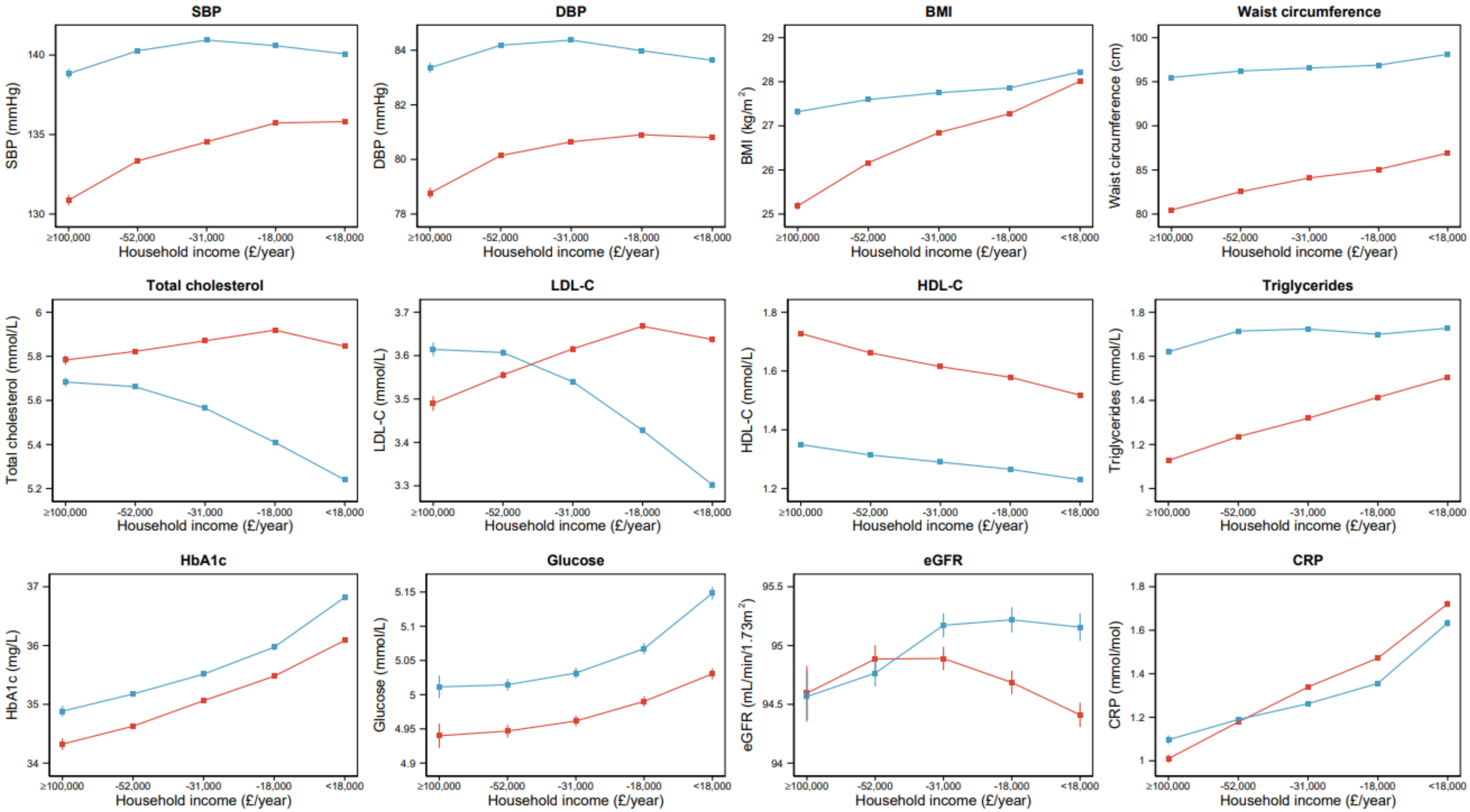

Supplemental Figure 9. Mean cardiometabolic risk factors, <u>by household income and sex, with further adjustment for number of people living in household</u>.

Values are means (95% confidence intervals) adjusted for age and number of people living in household. Red lines are for women, and blue lines are for men. Index of Multiple Deprivation fifths: ranging from 1 (least deprived) to 5 (most deprived). BMI = body

mass index; CRP = C-reactive protein; DBP = diastolic blood pressure; eGFR = estimated glomerular filtration rate; HbA1c = glycated haemoglobin; HDL-C = high-density lipoprotein cholesterol; LDL-C = low-density lipoprotein cholesterol; SBP = systolic blood pressure. Mean values by sex and household income with further adjustment for number of people living in household are provided in online supplemental table 13.

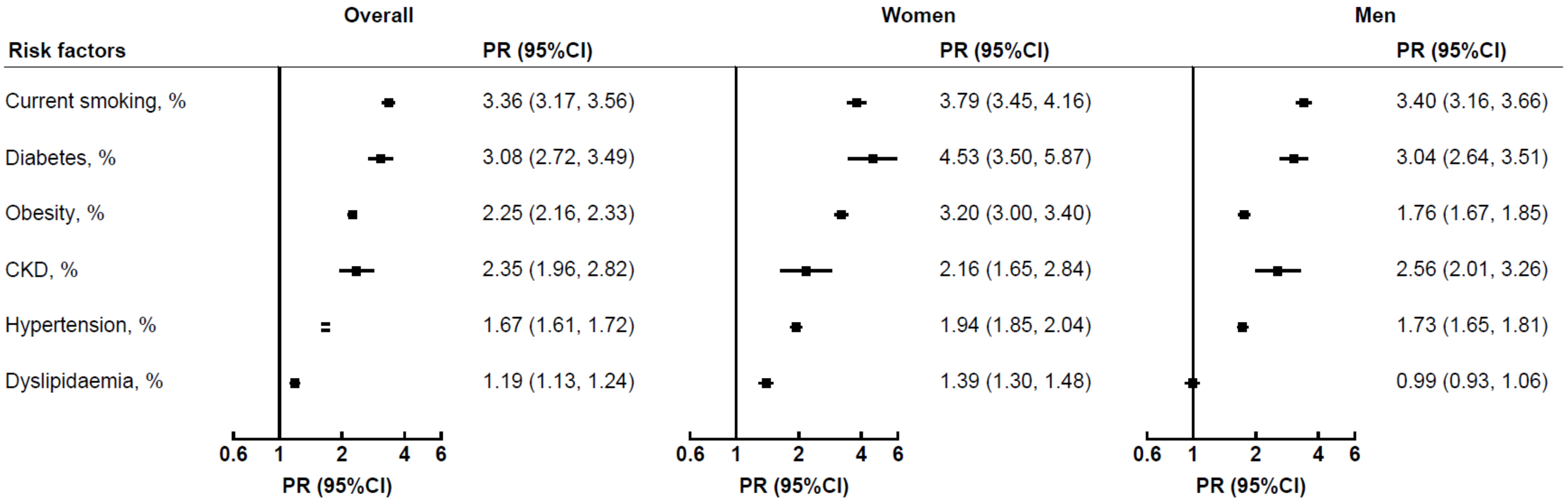

Supplemental Figure 10. Prevalence ratios for cardiometabolic risk factors in the lowest versus highest household income group, by sex, with further adjustment for number of people living in household.

Values are prevalence ratios (95% confidence intervals) adjusted for age. CI = confidence interval; CKD = chronic kidney disease; PR = prevalence ratio.

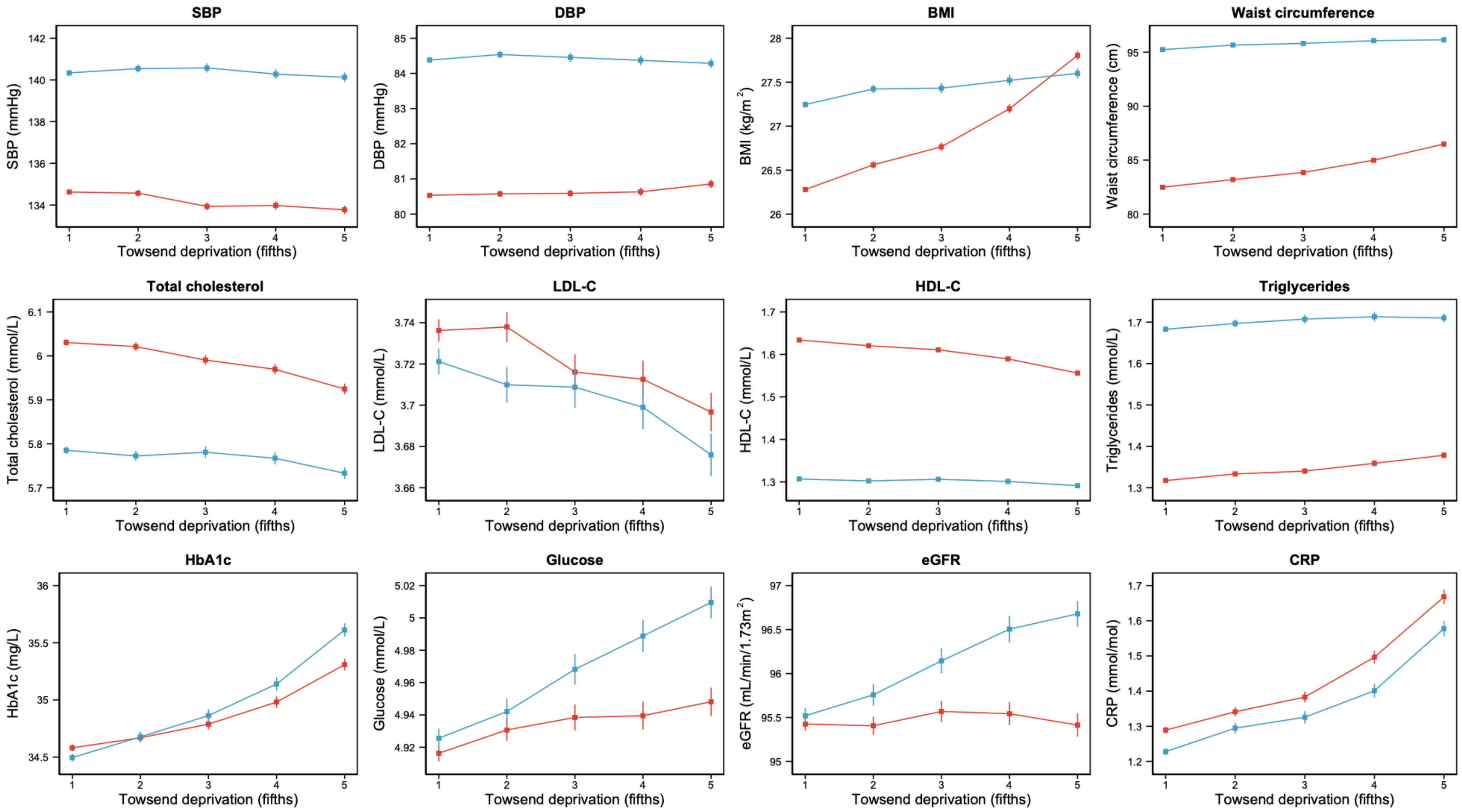

Supplemental Figure 11. Mean cardiometabolic risk factors, by sex and Townsend deprivation fifths, after excluding participants taking lipid-lowering medication.

Values are means (95% confidence intervals) adjusted for age. Red lines are for women, and blue lines are for men. Townsend deprivation fifths: 1 (Townsend deprivation score <-2.938, least deprived); 2 (≥-2.938-<-1.531); 3 (≥-1.531-<0.170); 4 (≥0.170-<2.448); and 5 (≥2.448, most deprived). BMI = body mass index; CRP = C-reactive protein; DBP = diastolic blood pressure; eGFR = estimated glomerular filtration rate; HbA1c = glycated haemoglobin; HDL-C = high-density lipoprotein cholesterol; LDL-C = low-density lipoprotein cholesterol; SBP = systolic blood pressure. Mean values by sex and Townsend deprivation fifths are provided in online supplemental table 15.

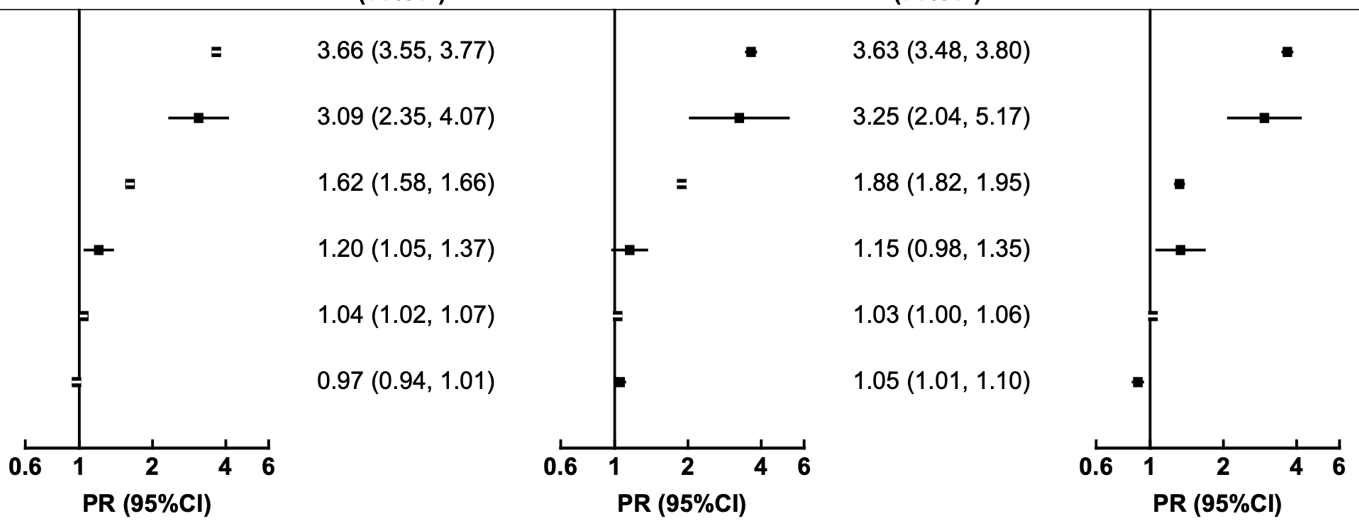

Risk factors	Overall PR (95%CI)	Women PR (95%CI)	Men PR (95%CI)
Current smoking, %	3.66 (3.55, 3.77)	3.63 (3.48, 3.80)	3.66 (3.51, 3.83)
Diabetes, %	3.09 (2.35, 4.07)	3.25 (2.04, 5.17)	2.95 (2.10, 4.14)
Obesity, %	1.62 (1.58, 1.66)	1.88 (1.82, 1.95)	1.32 (1.27, 1.37)
CKD, %	1.20 (1.05, 1.37)	1.15 (0.98, 1.35)	1.33 (1.06, 1.67)
Hypertension, %	1.04 (1.02, 1.07)	1.03 (1.00, 1.06)	1.03 (0.99, 1.06)
Dyslipidaemia, %	0.97 (0.94, 1.01)	1.05 (1.01, 1.10)	0.89 (0.85, 0.93)

Supplemental Figure 12. Prevalence ratios for cardiometabolic risk factors in the most versus least Townsend deprivation fifth, by sex, after excluding participants taking lipid-lowering medication.

Values are prevalence ratios (95% confidence intervals) adjusted for age. CI = confidence interval; CKD = chronic kidney disease; PR = prevalence ratio.

SUPPLEMENTAL TABLES

Supplemental Table 1. Number of participants who contributed to the baseline and follow-up analyses of each cardiometabolic risk factor, by Townsend Deprivation Score fifths.

	Townsend score fifths					
**Risk factors**	**1 (Least deprived)**	**2**	**3**	**4**	**5 (Most deprived)**	**Total**
N	186,076	101,892	74,496	67,267	71,038	500,769
SBP	16,411 (8.8%)	8,704 (8.5%)	6,432 (8.6%)	5,939 (8.8%)	7,163 (10.1%)	44,649 (8.9%)
DBP	16,406 (8.8%)	8,702 (8.5%)	6,430 (8.6%)	5,937 (8.8%)	7,162 (10.1%)	44,637 (8.9%)
BMI	479 (0.3%)	342 (0.3%)	319 (0.4%)	341 (0.5%)	565 (0.8%)	2,046 (0.4%)
Waist circumference	320 (0.2%)	248 (0.2%)	214 (0.3%)	233 (0.3%)	345 (0.5%)	1,360 (0.3%)
Total cholesterol	11,259 (6.1%)	6,428 (6.3%)	4,736 (6.4%)	4,377 (6.5%)	5,255 (7.4%)	32,055 (6.4%)
LDL-C	11,578 (6.2%)	6,586 (6.5%)	4,846 (6.5%)	4,490 (6.7%)	5,437 (7.7%)	32,937 (6.6%)
HDL-C	26,135 (14.0%)	14,503 (14.2%)	10,562 (14.2%)	9,691 (14.4%)	10,808 (15.2%)	71,699 (14.3%)
Triglycerides	11,382 (6.1%)	6,491 (6.4%)	4,785 (6.4%)	4,440 (6.6%)	5,331 (7.5%)	32,429 (6.5%)
HbA1c	12,039 (6.5%)	6,817 (6.7%)	5,229 (7.0%)	5,029 (7.5%)	6,019 (8.5%)	35,133 (7.0%)
Glucose	26,255 (14.1%)	14,566 (14.3%)	10,604 (14.2%)	9,733 (14.5%)	10,843 (15.3%)	72,001 (14.4%)
eGFR	11,346 (6.1%)	6,467 (6.3%)	4,770 (6.4%)	4,412 (6.6%)	5,291 (7.4%)	32,286 (6.4%)
CRP	11,617 (6.2%)	6,603 (6.5%)	4,908 (6.6%)	4,533 (6.7%)	5,415 (7.6%)	33,076 (6.6%)

BMI = body mass index; CRP = C-reactive protein; DBP = diastolic blood pressure; eGFR = estimated glomerular filtration rate; HbA1c = glycated haemoglobin; HDL-C = high-density lipoprotein cholesterol; LDL-C = low-density lipoprotein cholesterol; SBP = systolic blood pressure; SES = socioeconomic status.

Townsend score fifths: 1 (Townsend score <-2.938, least deprived); 2 (≥-2.938-<-1.531); 3 (≥-1.531-<0.170); 4 (≥0.170-<2.448); and 5 (≥2.448, most deprived).

Supplemental Table 2. Participant characteristics by sex.

	Women	Men
N	272,492	228,277
Age (mean [SD]), years	56.4 (8.0)	56.8 (8.2)
Townsend deprivation index score (median [IQR])	-2.1 (-3.6, 0.5)	-2.1 (-3.7, 0.6)
Towsend deprivation score fifths (n [%])		
1 (least deprived)	101,108 (37.1%)	84,968 (37.2%)
2	56,117 (20.6%)	45,775 (20.1%)
3	41,207 (15.1%)	33,289 (14.6%)
4	36,865 (13.5%)	30,402 (13.3%)
5 (most deprived)	37,195 (13.6%)	33,843 (14.8%)
Household income (n [%]), £/year		
≥100,000	10,699 (4.8%)	12,176 (6.0%)
52,000-<100,000	41,525 (18.6%)	44,512 (22.1%)
31,000-<52,000	56,609 (25.4%)	53,886 (26.8%)
18,000-<31,000	58,697 (26.3%)	49,230 (24.4%)
<18,000	55,244 (24.8%)	41,638 (20.7%)
Educational attainment (n [%])[b]		
University/College degree	84,289 (31.5%)	76,417 (34.2%)
Education to ≥18 years	84,817 (31.7%)	77,405 (34.6%)
Education to ≥16 years	52,387 (19.6%)	30,706 (13.7%)
No qualifications	45,831 (17.1%)	39,169 (17.5%)

Values are means for continuous variables adjusted for age, unless otherwise specified. Values between brackets indicate 95% confidence intervals, unless otherwise specified.

Townsend deprivation fifths: 1 (Townsend deprivation score <-2.938, least deprived); 2 (≥-2.938-<-1.531); 3 (≥-1.531-<0.170); 4 (≥0.170-<2.448); and 5 (≥2.448, most deprived).

Supplemental Table 3. Cardiometabolic risk factors, treatment, and control, by Townsend Deprivation Score fifths.

	1 (Least deprived)	2	3	4	5 (Most deprived)	Δ between most and least deprived	P-Trend[a]
N	186,076	101,892	74,496	67,267	71,038	-	-
**Risk factors**							
SBP, mmHg	138.0 (137.9, 138.1)	138.0 (137.9, 138.1)	137.6 (137.5, 137.8)	137.5 (137.4, 137.6)	137.4 (137.3, 137.5)	-0.596 (-0.757, -0.436)	0.068
DBP, mmHg	82.2 (82.2, 82.3)	82.2 (82.2, 82.3)	82.2 (82.1, 82.3)	82.1 (82.1, 82.2)	82.2 (82.1, 82.2)	-0.037 (-0.130, 0.055)	0.483
BMI, kg/m2	27.0 (27.0, 27.0)	27.3 (27.3, 27.3)	27.5 (27.4, 27.5)	27.8 (27.8, 27.9)	28.3 (28.3, 28.4)	1.31 (1.27, 1.35)	<0.001
Waist circumference, cm	89.2 (89.1, 89.2)	89.8 (89.7, 89.9)	90.4 (90.3, 90.5)	91.4 (91.3, 91.5)	92.9 (92.8, 93.0)	3.69 (3.57, 3.80)	<0.001
Total cholesterol, mmol/L	5.75 (5.74, 5.75)	5.72 (5.71, 5.72)	5.69 (5.68, 5.70)	5.65 (5.64, 5.66)	5.56 (5.55, 5.56)	-0.191 (-0.201, -0.181)	<0.001
LDL-C, mmol/L	3.59 (3.59, 3.60)	3.57 (3.57, 3.58)	3.55 (3.55, 3.56)	3.53 (3.52, 3.53)	3.47 (3.46, 3.48)	-0.123 (-0.131, -0.115)	<0.001
HDL-C, mmol/L	1.47 (1.47, 1.47)	1.46 (1.45, 1.46)	1.45 (1.45, 1.45)	1.43 (1.43, 1.44)	1.40 (1.39, 1.40)	-0.070 (-0.074, -0.066)	<0.001
Triglycerides, mmol/L	1.50 (1.49, 1.50)	1.51 (1.51, 1.52)	1.52 (1.52, 1.53)	1.54 (1.54, 1.55)	1.57 (1.56, 1.57)	0.070 (0.062, 0.077)	0.055
HbA1c, mmol/mol	35.2 (35.2, 35.2)	35.4 (35.4, 35.5)	35.7 (35.6, 35.7)	36.1 (36.0, 36.1)	36.8 (36.7, 36.8)	1.56 (1.52, 1.61)	<0.001
Glucose, mmol/L	4.99 (4.99, 4.99)	5.02 (5.01, 5.02)	5.04 (5.03, 5.04)	5.07 (5.06, 5.08)	5.11 (5.11, 5.12)	0.124 (0.116, 0.133)	<0.001
eGFR, mL/min/1.73m2	94.5 (94.4, 94.5)	94.5 (94.4, 94.6)	94.7 (94.6, 94.8)	94.8 (94.7, 94.8)	94.6 (94.5, 94.7)	0.151 (0.044, 0.257)	0.009
CRP, mg/L	1.28 (1.28, 1.29)	1.35 (1.34, 1.36)	1.40 (1.39, 1.41)	1.50 (1.48, 1.51)	1.69 (1.68, 1.71)	0.415 (0.399, 0.430)	<0.001
CKD, %	1.42 (1.36, 1.47)	1.58 (1.50, 1.66)	1.63 (1.53, 1.72)	1.89 (1.78, 1.99)	2.36 (2.24, 2.48)	0.944 (0.810, 1.08)	0.001
**Prevalence, treatment and control**							
Current smoking, %	6.68 (6.57, 6.80)	8.54 (8.37, 8.71)	10.48 (10.26, 10.70)	13.9 (13.6, 14.1)	20.4 (20.1, 20.7)	13.7 (13.4, 14.0)	<0.001
Hypertension, %	55.0 (54.8, 55.2)	56.2 (55.9, 56.5)	56.2 (55.8, 56.5)	57.4 (57.1, 57.8)	60.1 (59.8, 60.5)	5.12 (4.70, 5.55)	<0.001
Treated, %	36.2 (35.9, 36.5)	38.2 (37.8, 38.6)	39.6 (39.1, 40.1)	42.1 (41.6, 42.6)	46.8 (46.3, 47.3)	10.6 (10.0, 11.2)	<0.001
Controlled among treated, %	38.5 (38.0, 39.0)	38.5 (37.8, 39.1)	40.3 (39.5, 41.0)	40.8 (40.0, 41.6)	42.8 (42.1, 43.6)	0.470 (-1.54, 2.48)	<0.001
Obesity, %	21.2 (21.0, 21.4)	23.6 (23.3, 23.8)	25.3 (25.0, 25.6)	28.1 (27.8, 28.5)	32.4 (32.0, 32.7)	11.2 (10.8, 11.6)	<0.001
Dyslipidaemia, %	87.5 (87.3, 87.6)	87.7 (87.5, 88.0)	87.8 (87.5, 88.0)	87.6 (87.3, 87.8)	87.7 (87.5, 88.0)	0.244 (-0.054, 0.542)	0.229
Treated, %	18.0 (17.8, 18.2)	19.4 (19.1, 19.6)	20.6 (20.2, 20.9)	23.2 (22.8, 23.6)	28.4 (28.0, 28.8)	10.4 (9.94, 10.8)	<0.001
Controlled among treated, %	32.7 (32.3, 33.2)	33.1 (32.5, 33.8)	32.6 (31.9, 33.4)	32.6 (31.9, 33.4)	34.2 (33.5, 34.9)	1.45 (0.603, 2.30)	0.032
Diabetes, %	2.22 (2.15, 2.29)	2.64 (2.54, 2.74)	3.04 (2.91, 3.17)	3.85 (3.69, 4.00)	5.37 (5.19, 5.55)	3.15 (2.96, 3.34)	<0.001
Treated, %	60.2 (59.1, 61.3)	61.4 (60.0, 62.8)	62.6 (61.1, 64.2)	65.2 (63.8, 66.6)	66.0 (64.8, 67.3)	5.85 (4.18, 7.52)	0.752
Controlled among treated, %	29.8 (28.5, 31.1)	28.5 (27.0, 30.1)	30.0 (28.3, 31.7)	30.5 (28.8, 32.1)	29.7 (28.3, 31.0)	-0.132 (-1.99, 1.72)	0.406
CKD, %	1.42 (1.36, 1.47)	1.58 (1.50, 1.66)	1.63 (1.53, 1.72)	1.89 (1.78, 1.99)	2.36 (2.24, 2.48)	0.944 (0.810, 1.08)	0.001
**Number of risk factors[b]**							
Number of risk factors	1.55 (1.55, 1.56)	1.61 (1.61, 1.62)	1.65 (1.64, 1.66)	1.73 (1.72, 1.73)	1.86 (1.85, 1.86)	0.304 (0.296, 0.312)	<0.001
Number of risk factors, %							
0	11.8 (11.7, 12.0)	11.0 (10.8, 11.2)	10.6 (10.4, 10.8)	9.73 (9.51, 9.9)	8.31 (8.12, 8.50)	-3.50 (-3.74, -3.26)	<0.001
1	37.2 (37.0, 37.4)	35.5 (35.2, 35.8)	34.5 (34.1, 34.8)	32.4 (32.0, 32.7)	28.7 (28.4, 29.0)	-8.48 (-8.87, -8.08)	<0.001

2	36.6 (36.4, 36.9)	36.9 (36.6, 37.2)	36.6 (36.2, 36.9)	36.6 (36.3, 37.0)	37.2 (36.8, 37.5)	0.549 (0.133, 0.965)	0.107
≥3	14.5 (14.4, 14.7)	16.6 (16.4, 16.9)	18.3 (18.1, 18.6)	21.3 (21.0, 21.6)	26.2 (25.8, 26.5)	11.6 (11.3, 12.0)	<0.001

Values are means for continuous variables adjusted for age, unless otherwise specified. Values between brackets indicate 95% confidence intervals, unless otherwise specified.

Townsend score fifths: 1 (Townsend score <-2.938, least deprived); 2 (≥-2.938-<-1.531); 3 (≥-1.531-<0.170); 4 (≥0.170-<2.448); and 5 (≥2.448, most deprived).

BMI = body mass index; CKD = chronic kidney disease; CRP = C-reactive protein; DBP = diastolic blood pressure; eGFR = estimated glomerular filtration rate; HbA1c = glycated haemoglobin; HDL-C = high-density lipoprotein cholesterol; LDL-C = low-density lipoprotein cholesterol; SBP = systolic blood pressure.

[a]P values for sex-specific linear trends across Townsend score fifths.

[b]Risk factors included were current smoking, hypertension, obesity, dyslipidaemia and diabetes.

Supplemental Table 4. Cardiometabolic risk factors, treatment, and control, by Townsend Deprivation Score fifths and sex.

Risk factors	1 (Least deprived)	2	3	4	5 (Most deprived)	Δ between most and least deprived	P-Trend[a]	P-Het[b]
N								
Women:	101,108	56,117	41,207	36,865	37,195	-	-	-
Men:	84,968	45,775	33,289	30,402	33,843	-	-	-
**Risk factors**								
SBP, mmHg								
Women:	135.7 (135.6, 135.8)	135.6 (135.5, 135.8)	135.1 (134.9, 135.2)	135.0 (134.8, 135.2)	134.8 (134.6, 135.0)	-0.928 (-1.15, -0.710)	0.088	<0.001
Men:	140.7 (140.6, 140.8)	140.9 (140.7, 141.0)	140.8 (140.6, 141.0)	140.5 (140.3, 140.7)	140.2 (140.0, 140.4)	-0.488 (-0.719, -0.257)	<0.001	
DBP, mmHg								
Women:	80.6 (80.6, 80.7)	80.7 (80.6, 80.7)	80.7 (80.6, 80.8)	80.7 (80.6, 80.8)	80.8 (80.7, 80.9)	0.129 (0.004, 0.254)	0.684	<0.001
Men:	84.1 (84.0, 84.1)	84.2 (84.1, 84.3)	84.1 (84.0, 84.2)	83.9 (83.8, 84.0)	83.7 (83.6, 83.8)	-0.392 (-0.524, -0.259)	<0.001	
BMI, kg/m^2								
Women:	26.5 (26.5, 26.6)	26.9 (26.9, 26.9)	27.1 (27.1, 27.2)	27.6 (27.6, 27.7)	28.4 (28.3, 28.4)	1.82 (1.77, 1.88)	<0.001	<0.001
Men:	27.6 (27.5, 27.6)	27.8 (27.8, 27.8)	27.9 (27.9, 28.0)	28.1 (28.0, 28.1)	28.3 (28.2, 28.3)	0.698 (0.638, 0.758)	0.015	
Waist circumference, cm								
Women:	83.3 (83.2, 83.4)	84.2 (84.1, 84.3)	84.9 (84.8, 85.0)	86.3 (86.1, 86.4)	88.1 (87.9, 88.2)	4.78 (4.63, 4.92)	<0.001	<0.001
Men:	96.2 (96.1, 96.3)	96.8 (96.7, 96.9)	97.2 (97.1, 97.3)	97.6 (97.5, 97.7)	98.1 (98.0, 98.2)	1.88 (1.73, 2.03)	0.381	
Total cholesterol, mmol/L								
Women:	5.94 (5.93, 5.94)	5.91 (5.90, 5.91)	5.86 (5.85, 5.87)	5.82 (5.80, 5.83)	5.72 (5.71, 5.74)	-0.213 (-0.227, -0.199)	<0.001	<0.001
Men:	5.52 (5.51, 5.53)	5.49 (5.48, 5.50)	5.48 (5.47, 5.50)	5.45 (5.44, 5.46)	5.37 (5.36, 5.39)	-0.148 (-0.163, -0.133)	<0.001	
LDL-C, mmol/L								
Women:	3.66 (3.66, 3.67)	3.65 (3.64, 3.65)	3.61 (3.61, 3.62)	3.59 (3.58, 3.60)	3.54 (3.53, 3.55)	-0.123 (-0.133, -0.112)	<0.001	0.067
Men:	3.51 (3.50, 3.52)	3.49 (3.48, 3.49)	3.48 (3.47, 3.49)	3.45 (3.44, 3.46)	3.39 (3.38, 3.40)	-0.117 (-0.123, -0.133)	<0.001	
HDL-C, mmol/L								
Women:	1.62 (1.62, 1.62)	1.60 (1.60, 1.61)	1.59 (1.59, 1.60)	1.57 (1.56, 1.57)	1.53 (1.53, 1.53)	-0.092 (-0.096, -0.087)	<0.001	<0.001
Men:	1.29 (1.29, 1.29)	1.28 (1.28, 1.28)	1.28 (1.28, 1.28)	1.27 (1.27, 1.28)	1.26 (1.25, 1.26)	-0.031 (-0.036, -0.026)	<0.001	
Triglycerides, mmol/L								
Women:	1.35 (1.34, 1.35)	1.37 (1.36, 1.37)	1.37 (1.37, 1.38)	1.40 (1.39, 1.41)	1.43 (1.42, 1.43)	0.077 (0.068, 0.086)	0.008	<0.001
Men:	1.69 (1.69, 1.70)	1.71 (1.70, 1.72)	1.72 (1.72, 1.73)	1.73 (1.72, 1.74)	1.73 (1.72, 1.74)	0.040 (0.029, 0.052)	0.154	
HbA1c, mmol/mol								
Women:	35.1 (35.1, 35.1)	35.3 (35.2, 35.3)	35.5 (35.4, 35.5)	35.8 (35.7, 35.8)	36.4 (36.3, 36.4)	1.29 (1.22, 1.36)	<0.001	<0.001
Men:	35.4 (35.3, 35.4)	35.6 (35.6, 35.7)	35.9 (35.9, 36.0)	36.4 (36.3, 36.5)	37.2 (37.1, 37.3)	1.86 (1.78, 1.93)	<0.001	
Glucose, mmol/L								
Women:	4.97 (4.96, 4.97)	4.99 (4.98, 5.00)	5.00 (4.99, 5.01)	5.02 (5.01, 5.03)	5.06 (5.05, 5.07)	0.090 (0.079, 0.102)	0.006	<0.001
Men:	5.02 (5.01, 5.02)	5.05 (5.04, 5.06)	5.07 (5.06, 5.09)	5.13 (5.12, 5.14)	5.18 (5.17, 5.19)	0.159 (0.146, 0.171)	0.014	
eGFR, mL/min/1.73m^2								
Women:	94.5 (94.4, 94.6)	94.4 (94.3, 94.5)	94.5 (94.4, 94.6)	94.4 (94.3, 94.5)	94.1 (94.0, 94.2)	-0.418 (-0.564, -0.271)	0.008	<0.001

Men:	94.4 (94.4, 94.5)	94.6 (94.5, 94.7)	94.9 (94.8, 95.0)	95.2 (95.0, 95.3)	95.2 (95.1, 95.3)	0.774 (0.620, 0.929)	0.253	
CRP, mg/L								
Women:	1.32 (1.32, 1.33)	1.39 (1.38, 1.40)	1.43 (1.42, 1.45)	1.55 (1.53, 1.57)	1.76 (1.74, 1.78)	0.438 (0.416, 0.459)	<0.001	0.184
Men:	1.23 (1.23, 1.24)	1.31 (1.29, 1.32)	1.35 (1.34, 1.37)	1.43 (1.42, 1.45)	1.62 (1.61, 1.64)	0.395 (0.373, 0.416)	<0.001	
**Prevalence, treatment and control**								
Current smoking, %								
Women:	5.46 (5.32, 5.60)	7.21 (7.00, 7.43)	9.00 (8.72, 9.27)	11.9 (11.6, 12.2)	17.5 (17.1, 17.8)	12.0 (11.6, 12.5)	<0.001	0.064
Men:	8.16 (7.97, 8.35)	10.2 (9.91, 10.5)	12.3 (12.0, 12.7)	16.3 (15.9, 16.7)	23.6 (23.1, 24.0)	15.4 (14.9, 15.9)	<0.001	
Hypertension, %								
Women:	49.0 (48.7, 49.3)	50.1 (49.7, 50.5)	50.0 (49.6, 50.5)	51.5 (51.0, 52.0)	53.9 (53.4, 54.4)	4.91 (4.32, 5.51)	<0.001	0.574
Men:	62.2 (61.9, 62.6)	63.7 (63.2, 64.1)	63.8 (63.3, 64.3)	64.6 (64.0, 65.1)	66.9 (66.4, 67.4)	4.63 (4.04, 5.22)	<0.001	
Treated, %								
Women:	33.8 (33.4, 34.2)	36.6 (36.1, 37.2)	38.0 (37.3, 38.7)	40.8 (40.0, 41.5)	46.1 (45.3, 46.8)	12.3 (11.5, 13.2)	<0.001	<0.001
Men:	38.4 (37.9, 38.8)	39.7 (39.2, 40.3)	41.2 (40.5, 41.9)	43.3 (42.6, 44.1)	47.4 (46.7, 48.1)	9.02 (8.22, 9.82)	<0.001	
Controlled among treated, %								
Women:	39.6 (38.8, 40.3)	39.8 (38.8, 40.8)	41.6 (40.5, 42.8)	43.3 (42.1, 44.4)	44.3 (43.2, 45.4)	4.72 (3.36, 6.07)	<0.001	0.081
Men:	37.7 (37.0, 38.3)	37.3 (36.4, 38.2)	39.0 (38.0, 40.1)	38.5 (37.4, 39.6)	41.6 (40.6, 42.6)	3.94 (2.71, 5.17)	<0.001	
Obesity, %								
Women:	19.8 (19.6, 20.1)	22.1 (21.8, 22.5)	24.2 (23.8, 24.6)	28.1 (27.7, 28.6)	33.5 (33.0, 34.0)	13.7 (13.1, 14.2)	<0.001	<0.001
Men:	22.9 (22.6, 23.2)	25.3 (24.9, 25.7)	26.6 (26.1, 27.0)	28.1 (27.6, 28.6)	31.1 (30.6, 31.6)	8.24 (7.67, 8.82)	<0.001	
Dyslipidaemia, %								
Women:	88.0 (87.8, 88.3)	88.3 (88.1, 88.6)	88.2 (87.9, 88.6)	88.1 (87.7, 88.4)	88.3 (87.9, 88.6)	0.211 (-0.194, 0.616)	<0.001	0.873
Men:	86.8 (86.5, 87.0)	87.0 (86.7, 87.3)	87.2 (86.8, 87.6)	87.0 (86.6, 87.4)	87.1 (86.8, 87.5)	0.351 (-0.087, 0.789)	<0.001	
Treated, %								
Women:	12.3 (12.0, 12.5)	13.9 (13.6, 14.2)	15.0 (14.6, 15.4)	17.2 (16.8, 17.7)	22.1 (21.6, 22.6)	9.82 (9.26, 10.4)	<0.001	<0.001
Men:	24.6 (24.2, 24.9)	25.9 (25.5, 26.4)	27.3 (26.8, 27.9)	30.4 (29.8, 31.0)	35.2 (34.6, 35.8)	10.6 (9.91, 11.3)	<0.001	
Controlled among treated, %								
Women:	22.8 (22.1, 23.4)	24.6 (23.7, 25.5)	24.5 (23.5, 25.5)	25.1 (24.1, 26.2)	27.0 (26.0, 28.0)	4.22 (3.04, 5.39)	<0.001	<0.001
Men:	40.1 (39.4, 40.7)	39.9 (39.0, 40.8)	39.3 (38.2, 40.3)	38.7 (37.7, 39.8)	39.9 (39.0, 40.9)	-0.160 (-1.33, 1.01)	<0.001	
Diabetes, %								
Women:	1.48 (1.41, 1.56)	1.88 (1.77, 2.00)	2.19 (2.04, 2.34)	2.76 (2.59, 2.94)	4.06 (3.84, 4.27)	2.55 (2.32, 2.77)	<0.001	0.003
Men:	3.07 (2.95, 3.18)	3.55 (3.38, 3.72)	4.06 (3.84, 4.28)	5.12 (4.86, 5.38)	6.78 (6.49, 7.06)	3.75 (3.44, 4.07)	<0.001	
Treated, %								
Women:	57.0 (55.1, 58.9)	58.0 (55.8, 60.3)	59.1 (56.6, 61.5)	61.9 (59.6, 64.2)	64.1 (62.1, 66.0)	7.06 (4.33, 9.78)	<0.001	0.722
Men:	62.1 (60.7, 63.5)	63.5 (61.8, 65.3)	65.0 (63.0, 66.9)	67.4 (65.5, 69.2)	67.3 (65.7, 68.9)	5.19 (3.08, 7.29)	<0.001	
Controlled among treated, %								
Women:	27.6 (25.6, 29.7)	26.7 (24.3, 29.2)	28.3 (25.5, 31.0)	30.9 (28.3, 33.5)	28.2 (26.1, 30.4)	0.579 (-2.39, 3.54)	<0.001	0.390
Men:	31.0 (29.4, 32.6)	29.6 (27.7, 31.6)	31.1 (28.9, 33.3)	30.2 (28.1, 32.2)	30.6 (28.8, 32.3)	-0.428 (-2.79, 1.94)	<0.001	
CKD, %								

Women:	1.44 (1.36, 1.51)	1.58 (1.47, 1.68)	1.62 (1.49, 1.75)	1.84 (1.69, 1.99)	2.33 (2.16, 2.50)	0.870 (0.690, 1.05)	<0.001	0.781
Men:	1.39 (1.32, 1.47)	1.59 (1.47, 1.70)	1.64 (1.50, 1.78)	1.94 (1.78, 2.10)	2.39 (2.22, 2.57)	1.03 (0.830, 1.23)	<0.001	
**Number of risk factors[c]**								
Number of risk factors								
Women:	1.46 (1.46, 1.47)	1.52 (1.51, 1.52)	1.56 (1.55, 1.56)	1.64 (1.63, 1.65)	1.77 (1.76, 1.78)	0.305 (0.294, 0.316)	<0.001	0.049
Men:	1.66 (1.66, 1.67)	1.73 (1.72, 1.74)	1.77 (1.76, 1.78)	1.83 (1.82, 1.84)	1.96 (1.95, 1.96)	0.293 (0.281, 0.304)	<0.001	
Number of risk factors, %								
0								
Women:	13.5 (13.3, 13.7)	12.7 (12.4, 12.9)	12.1 (11.8, 12.4)	11.1 (10.8, 11.4)	9.83 (9.55, 10.1)	-3.69 (-4.05, -3.34)	<0.001	0.349
Men:	9.74 (9.54, 9.94)	8.95 (8.69, 9.21)	8.65 (8.35, 8.94)	8.03 (7.74, 8.33)	6.63 (6.38, 6.89)	-3.07 (-3.39, -2.75)	<0.001	
1								
Women:	40.2 (39.9, 40.5)	38.5 (38.1, 38.9)	37.5 (37.1, 38.0)	34.9 (34.4, 35.4)	30.7 (30.2, 31.2)	-9.48 (-10.0, -8.93)	<0.001	<0.001
Men:	33.6 (33.3, 33.9)	31.8 (31.3, 32.2)	30.7 (30.2, 31.2)	29.3 (28.8, 29.9)	26.6 (26.1, 27.0)	-7.06 (-7.62, -6.49)	<0.001	
2								
Women:	34.5 (34.2, 34.8)	34.9 (34.5, 35.3)	34.6 (34.1, 35.1)	35.3 (34.8, 35.8)	36.3 (35.8, 36.8)	1.75 (1.19, 2.32)	<0.001	<0.001
Men:	39.1 (38.8, 39.5)	39.3 (38.8, 39.7)	39.0 (38.5, 39.6)	38.2 (37.7, 38.7)	38.2 (37.6, 38.7)	-0.988 (-1.60, -0.377)	<0.001	
≥3								
Women:	12.1 (11.9, 12.3)	14.0 (13.7, 14.3)	15.7 (15.4, 16.1)	18.6 (18.2, 19.0)	23.4 (22.9, 23.8)	11.2 (10.8, 11.7)	<0.001	<0.001
Men:	17.4 (17.1, 17.7)	19.8 (19.5, 20.2)	21.6 (21.1, 22.0)	24.5 (24.0, 25.0)	29.2 (28.7, 29.7)	11.8 (11.3, 12.4)	<0.001	

Values are means for continuous variables adjusted for age, unless otherwise specified. Values between brackets indicate 95% confidence intervals, unless otherwise specified.

Townsend score fifths: 1 (Townsend score <-2.938, least deprived); 2 (≥-2.938-<-1.531); 3 (≥-1.531-<0.170); 4 (≥0.170-<2.448); and 5 (≥2.448, most deprived).

BMI = body mass index; CKD = chronic kidney disease; CRP = C-reactive protein; DBP = diastolic blood pressure; eGFR = estimated glomerular filtration rate; HbA1c = glycated haemoglobin; HDL-C = high-density lipoprotein cholesterol; LDL-C = low-density lipoprotein cholesterol; SBP = systolic blood pressure.

[a]*P* values for sex-specific linear trends across Townsend score fifths.

[b]*P* values for sex differences in linear trends across Townsend score fifths.

[c]Risk factors included were current smoking, hypertension, obesity, dyslipidaemia and diabetes.

Supplemental Table 5. Cardiometabolic risk factors, treatment, and control, by household income.

	≥£100,000	£52,000-<£100,000	£31,000-<£52,000	£18,000-<£31,000	<£18,000	Δ between lowest and highest income	*P*-Trend[a]
N	22,875	86,037	110,495	107,927	96,882		-
**Risk factors**							
SBP, mmHg	135.2 (135.0, 135.5)	137.0 (136.9, 137.1)	137.7 (137.6, 137.8)	137.9 (137.8, 138.0)	137.6 (137.4, 137.7)	2.35 (2.08, 2.62)	<0.001
DBP, mmHg	81.2 (81.1, 81.3)	82.2 (82.2, 82.3)	82.5 (82.4, 82.5)	82.3 (82.2, 82.4)	82.0 (81.9, 82.1)	0.813 (0.658, 0.967)	<0.001
BMI, kg/m2	26.3 (26.3, 26.4)	26.9 (26.9, 27.0)	27.3 (27.3, 27.3)	27.5 (27.5, 27.6)	28.1 (28.1, 28.1)	1.74 (1.67, 1.81)	<0.001
Waist circumference, cm	88.7 (88.6, 88.9)	89.9 (89.8, 89.9)	90.3 (90.2, 90.3)	90.3 (90.2, 90.4)	91.5 (91.4, 91.6)	2.75 (2.55, 2.94)	<0.001
Total cholesterol, mmol/L	5.71 (5.70, 5.73)	5.73 (5.72, 5.73)	5.72 (5.71, 5.72)	5.69 (5.68, 5.70)	5.60 (5.59, 5.61)	-0.111 (-0.128, -0.094)	<0.001
LDL-C, mmol/L	3.55 (3.54, 3.56)	3.58 (3.57, 3.58)	3.58 (3.57, 3.58)	3.56 (3.55, 3.57)	3.50 (3.49, 3.50)	-0.050 (-0.063, -0.037)	<0.001
HDL-C, mmol/L	1.51 (1.51, 1.52)	1.47 (1.47, 1.47)	1.45 (1.45, 1.45)	1.44 (1.44, 1.44)	1.40 (1.40, 1.41)	-0.109 (-0.115, -0.103)	<0.001
Triglycerides, mmol/L	1.38 (1.37, 1.39)	1.47 (1.47, 1.48)	1.51 (1.50, 1.51)	1.53 (1.53, 1.54)	1.59 (1.58, 1.59)	0.214 (0.203, 0.225)	<0.001
HbA1c, mmol/mol	34.7 (34.6, 34.7)	34.9 (34.9, 35.0)	35.3 (35.3, 35.3)	35.7 (35.7, 35.7)	36.4 (36.3, 36.4)	1.71 (1.63, 1.79)	<0.001
Glucose, mmol/L	4.98 (4.97, 4.99)	4.98 (4.98, 4.99)	5.00 (4.99, 5.00)	5.03 (5.02, 5.03)	5.08 (5.08, 5.09)	0.104 (0.090, 0.117)	<0.001
eGFR, mL/min/1.73m^2	94.5 (94.3, 94.7)	94.8 (94.7, 94.9)	95.0 (94.9, 95.1)	94.9 (94.9, 95.0)	94.8 (94.7, 94.9)	0.275 (0.098, 0.453)	<0.001
CRP, mg/L	1.05 (1.04, 1.07)	1.18 (1.18, 1.19)	1.30 (1.29, 1.31)	1.42 (1.41, 1.43)	1.68 (1.67, 1.69)	0.631 (0.611, 0.650)	<0.001
**Prevalence, treatment and control**							
Current smoking, %	5.61 (5.33, 5.88)	6.59 (6.43, 6.75)	8.63 (8.47, 8.79)	11.5 (11.3, 11.7)	18.2 (17.9, 18.4)	12.6 (12.2, 12.9)	<0.001
Hypertension, %	48.1 (47.4, 48.7)	52.4 (52.1, 52.7)	55.1 (54.8, 55.4)	56.8 (56.5, 57.1)	59.5 (59.2, 59.9)	11.5 (10.7, 12.2)	<0.001
Treated, %	33.6 (32.6, 34.6)	34.3 (33.8, 34.8)	36.5 (36.1, 36.9)	39.0 (38.6, 39.4)	43.8 (43.4, 44.2)	10.2 (9.13, 11.3)	<0.001
Controlled among treated, %	41.2 (39.3, 43.1)	38.9 (38.0, 39.8)	38.9 (38.2, 39.6)	39.3 (38.7, 39.9)	41.7 (41.1, 42.3)	0.470 (-1.54, 2.48)	<0.001
Obesity, %	16.3 (15.8, 16.8)	20.4 (20.1, 20.7)	23.2 (22.9, 23.4)	25.4 (25.1, 25.6)	30.4 (30.1, 30.7)	14.1 (13.6, 14.7)	<0.001
Dyslipidaemia, %	86.0 (85.6, 86.5)	86.8 (86.6, 87.1)	87.1 (86.9, 87.3)	87.6 (87.4, 87.8)	88.1 (87.8, 88.3)	2.04 (1.54, 2.54)	0.406
Treated, %	18.2 (17.5, 18.9)	17.1 (16.8, 17.5)	18.1 (17.9, 18.4)	19.8 (19.5, 20.0)	24.3 (24.0, 24.6)	6.09 (5.35, 6.83)	<0.001
Controlled among treated, %	36.2 (34.5, 37.9)	33.4 (32.6, 34.3)	33.2 (32.6, 33.9)	32.9 (32.4, 33.5)	32.5 (32.0, 33.1)	-3.69 (-5.47, -1.91)	0.109
Diabetes, %	1.50 (1.32, 1.67)	1.98 (1.87, 2.08)	2.29 (2.19, 2.38)	3.01 (2.91, 3.11)	4.30 (4.17, 4.43)	2.80 (2.58, 3.03)	<0.001
Treated, %	57.4 (53.2, 61.5)	60.1 (58.2, 62.0)	61.0 (59.5, 62.4)	62.9 (61.6, 64.1)	65.1 (64.1, 66.2)	7.77 (3.49, 12.0)	0.813

Controlled among treated, %	29.8 (25.1, 34.6)	28.3 (26.2, 30.4)	30.8 (29.2, 32.5)	29.3 (27.9, 30.7)	30.1 (28.9, 31.3)	0.278 (-4.61, 5.17)	0.276
CKD, %	0.92 (0.76, 1.07)	1.09 (1.01, 1.18)	1.23 (1.16, 1.30)	1.55 (1.48, 1.62)	2.07 (1.99, 2.15)	1.15 (0.973, 1.33)	0.013
**Number of risk factors[b]**							
Number of risk factors	1.41 (1.40, 1.43)	1.52 (1.51, 1.52)	1.60 (1.59, 1.60)	1.67 (1.66, 1.68)	1.81 (1.81, 1.82)	0.398 (0.384, 0.411)	<0.001
Number of risk factors, %							
0	14.1 (13.7, 14.5)	12.1 (11.9, 12.3)	11.0 (10.8, 11.2)	9.80 (9.61, 10.0)	8.15 (7.96, 8.34)	-5.98 (-6.44, -5.53)	<0.001
1	41.5 (40.9, 42.2)	38.8 (38.4, 39.1)	36.4 (36.1, 36.7)	34.0 (33.8, 34.3)	29.4 (29.2, 29.7)	-12.1 (-12.8, -11.4)	<0.001
2	32.2 (31.5, 32.8)	35.2 (34.9, 35.5)	36.7 (36.4, 37.0)	37.6 (37.3, 37.8)	37.3 (37.0, 37.6)	5.16 (4.46, 5.85)	<0.001
≥3	11.1 (10.6, 11.5)	13.6 (13.3, 13.8)	16.0 (15.8, 16.2)	18.4 (18.2, 18.6)	24.0 (23.7, 24.3)	13.0 (12.5, 13.5)	<0.001

Values are means for continuous variables adjusted for age, unless otherwise specified. Values between brackets indicate 95% confidence intervals, unless otherwise specified.

Household income groups: ≥£100,000; £52,000-<£100,000, £31,000-<£52,000; £18,000-<£31,000, and <£18,000 per year.

BMI = body mass index; CKD = chronic kidney disease; CRP = C-reactive protein; DBP = diastolic blood pressure; eGFR = estimated glomerular filtration rate; HbA1c = glycated haemoglobin; HDL-C = high-density lipoprotein cholesterol; LDL-C = low-density lipoprotein cholesterol; SBP = systolic blood pressure.

[a]*P* values for sex-specific linear trends across household income groups.

[b]Risk factors included were current smoking, hypertension, obesity, dyslipidaemia and diabetes.

Supplemental Table 6. Cardiometabolic risk factors, treatment, and control, by household income and sex.

Risk factors	≥£100,000	£52,000-<£100,000	£31,000-<£52,000	£18,000-<£31,000	<£18,000	Δ between lowest and highest income	P-Trend[a]	P-Het[b]
N								
Women:	10,699	41,525	56,609	58,697	55,244		-	-
Men:	12,176	44,512	53,886	49,230	41,638		-	-
**Risk factors**								
SBP, mmHg								
Women:	130.9 (130.5, 131.2)	133.3 (133.2, 133.5)	134.5 (134.4, 134.7)	135.7 (135.6, 135.9)	135.8 (135.7, 136.0)	4.95 (4.58, 5.33)	<0.001	<0.001
Men:	138.8 (138.5, 139.2)	140.3 (140.1, 140.4)	140.9 (140.8, 141.1)	140.6 (140.4, 140.8)	140.1 (139.9, 140.3)	1.24 (0.878, 1.61)	<0.001	
DBP, mmHg								
Women:	78.7 (78.5, 78.9)	80.1 (80.0, 80.2)	80.6 (80.6, 80.7)	80.9 (80.8, 81.0)	80.9 (80.8, 80.9)	2.16 (1.94, 2.37)	<0.001	<0.001
Men:	83.3 (83.1, 83.5)	84.1 (84.0, 84.2)	84.4 (84.3, 84.4)	84.0 (83.9, 84.1)	83.7 (83.6, 83.8)	0.389 (0.178, 0.600)	<0.001	
BMI, kg/m^2								
Women:	25.2 (25.1, 25.3)	26.2 (26.1, 26.2)	26.8 (26.8, 26.9)	27.3 (27.2, 27.3)	28.0 (28.0, 28.0)	2.82 (2.72, 2.92)	<0.001	<0.001
Men:	27.3 (27.2, 27.4)	27.6 (27.6, 27.6)	27.8 (27.7, 27.8)	27.9 (27.8, 27.9)	28.2 (28.2, 28.3)	0.900 (0.803, 0.996)	<0.001	
Waist circumference, cm								
Women:	80.5 (80.3, 80.7)	82.6 (82.4, 82.7)	84.1 (84.0, 84.2)	85.1 (85.0, 85.2)	86.9 (86.8, 87.0)	6.37 (6.13, 6.62)	<0.001	<0.001
Men:	95.5 (95.3, 95.7)	96.3 (96.1, 96.4)	96.6 (96.5, 96.7)	96.9 (96.8, 97.0)	98.1 (98.0, 98.2)	2.56 (2.32, 2.80)	<0.001	
Total cholesterol, mmol/L								
Women:	5.77 (5.75, 5.79)	5.81 (5.80, 5.83)	5.87 (5.86, 5.88)	5.92 (5.91, 5.93)	5.86 (5.85, 5.87)	0.086 (0.062, 0.110)	<0.001	<0.001
Men:	5.67 (5.65, 5.69)	5.65 (5.64, 5.66)	5.56 (5.55, 5.57)	5.41 (5.40, 5.42)	5.25 (5.24, 5.26)	-0.423 (-0.446, -0.400)	<0.001	
LDL-C, mmol/L								
Women:	3.48 (3.47, 3.50)	3.55 (3.54, 3.56)	3.62 (3.61, 3.62)	3.67 (3.66, 3.68)	3.64 (3.63, 3.65)	0.158 (0.140, 0.177)	<0.001	<0.001
Men:	3.61 (3.59, 3.62)	3.60 (3.60, 3.61)	3.54 (3.53, 3.55)	3.43 (3.42, 3.44)	3.31 (3.30, 3.31)	-0.303 (0.158, 0.140)	<0.001	
HDL-C, mmol/L								
Women:	1.72 (1.71, 1.73)	1.66 (1.65, 1.66)	1.61 (1.61, 1.62)	1.58 (1.58, 1.58)	1.52 (1.52, 1.53)	-0.198 (-0.206, -0.190)	<0.001	<0.001
Men:	1.34 (1.34, 1.35)	1.31 (1.31, 1.31)	1.29 (1.29, 1.29)	1.27 (1.26, 1.27)	1.23 (1.23, 1.24)	-0.109 (-0.117, -0.102)	<0.001	
Triglycerides, mmol/L								
Women:	1.13 (1.12, 1.14)	1.24 (1.23, 1.24)	1.32 (1.31, 1.32)	1.41 (1.41, 1.42)	1.50 (1.50, 1.51)	0.375 (0.362, 0.388)	<0.001	<0.001
Men:	1.62 (1.61, 1.64)	1.71 (1.71, 1.72)	1.72 (1.72, 1.73)	1.70 (1.69, 1.71)	1.73 (1.72, 1.74)	0.106 (0.089, 0.124)	<0.001	
HbA1c, mmol/mol								
Women:	34.4 (34.3, 34.5)	34.6 (34.6, 34.7)	35.1 (35.0, 35.1)	35.5 (35.4, 35.5)	36.1 (36.0, 36.1)	1.70 (1.59, 1.81)	<0.001	<0.001
Men:	34.9 (34.8, 35.0)	35.2 (35.1, 35.2)	35.5 (35.5, 35.6)	36.0 (35.9, 36.0)	36.8 (36.7, 36.9)	1.89 (1.78, 2.00)	<0.001	
Glucose, mmol/L								
Women:	4.94 (4.92, 4.96)	4.95 (4.94, 4.95)	4.96 (4.95, 4.97)	4.99 (4.98, 5.00)	5.03 (5.02, 5.04)	0.094 (0.074, 0.114)	<0.001	<0.001
Men:	5.01 (4.99, 5.03)	5.01 (5.00, 5.02)	5.03 (5.02, 5.04)	5.07 (5.06, 5.08)	5.15 (5.14, 5.16)	0.140 (0.121, 0.159)	<0.001	

eGFR, mL/min/1.73m^2								
Women:	94.5 (94.3, 94.7)	94.8 (94.7, 95.0)	94.9 (94.8, 95.0)	94.7 (94.6, 94.8)	94.5 (94.4, 94.6)	-0.039 (-0.292, 0.214)	<0.001	<0.001
Men:	94.5 (94.3, 94.7)	94.7 (94.6, 94.8)	95.2 (95.1, 95.3)	95.2 (95.1, 95.3)	95.2 (95.1, 95.3)	0.708 (0.462, 0.954)	<0.001	
CRP, mg/L								
Women:	1.01 (0.99, 1.03)	1.18 (1.17, 1.19)	1.34 (1.33, 1.35)	1.47 (1.46, 1.48)	1.72 (1.70, 1.74)	0.710 (0.684, 0.736)	<0.001	<0.001
Men:	1.10 (1.08, 1.12)	1.19 (1.18, 1.20)	1.26 (1.25, 1.27)	1.36 (1.34, 1.37)	1.63 (1.62, 1.65)	0.540 (0.513, 0.567)	<0.001	
**Prevalence, treatment and control**								
Current smoking, %								
Women:	4.09 (3.75, 4.43)	5.12 (4.92, 5.31)	7.13 (6.92, 7.33)	9.7 (9.4, 9.9)	15.4 (15.1, 15.8)	11.5 (11.0, 12.0)	<0.001	0.019
Men:	6.97 (6.55, 7.40)	8.0 (7.75, 8.2)	10.3 (10.0, 10.5)	13.9 (13.6, 14.2)	22.0 (21.6, 22.4)	14.8 (14.2, 15.4)	<0.001	
Hypertension, %								
Women:	38.2 (37.2, 39.1)	44.3 (43.8, 44.8)	47.7 (47.3, 48.1)	50.8 (50.4, 51.2)	53.9 (53.5, 54.3)	15.7 (14.6, 16.8)	<0.001	<0.001
Men:	56.0 (55.2, 56.9)	59.6 (59.1, 60.0)	62.8 (62.4, 63.2)	64.4 (64.0, 64.9)	67.6 (67.1, 68.0)	11.4 (10.4, 12.4)	0.024	
Treated, %								
Women:	32.1 (30.4, 33.8)	31.8 (31.0, 32.6)	35.1 (34.5, 35.8)	36.8 (36.3, 37.4)	41.1 (40.6, 41.7)	9.02 (7.20, 10.8)	<0.001	<0.001
Men:	34.3 (33.1, 35.6)	35.8 (35.1, 36.4)	37.5 (37.0, 38.1)	41.0 (40.5, 41.6)	46.8 (46.2, 47.4)	12.4 (11.0, 13.8)	<0.001	
Controlled among treated, %								
Women:	45.0 (41.6, 48.4)	42.1 (40.5, 43.7)	41.6 (40.5, 42.7)	40.3 (39.4, 41.2)	43.1 (42.2, 43.9)	-1.95 (-5.45, 1.56)	<0.001	0.011
Men:	39.5 (37.2, 41.8)	37.3 (36.2, 38.4)	37.1 (36.2, 38.0)	38.5 (37.7, 39.3)	40.3 (39.5, 41.1)	0.830 (-1.62, 3.28)	0.004	
Obesity, %								
Women:	11.9 (11.3, 12.6)	17.8 (17.4, 18.1)	21.8 (21.5, 22.2)	24.8 (24.4, 25.1)	30.4 (30.0, 30.8)	18.4 (17.7, 19.2)	<0.001	<0.001
Men:	20.0 (19.3, 20.7)	22.8 (22.4, 23.2)	24.6 (24.2, 24.9)	26.2 (25.8, 26.6)	30.6 (30.2, 31.1)	10.6 (9.78, 11.5)	<0.001	
Dyslipidaemia, %								
Women:	84.0 (83.3, 84.7)	85.8 (85.4, 86.1)	87.1 (86.8, 87.4)	88.9 (88.6, 89.2)	90.0 (89.7, 90.3)	6.14 (5.39, 6.88)	0.445	<0.001
Men:	87.9 (87.3, 88.4)	87.9 (87.6, 88.2)	87.0 (86.7, 87.3)	85.9 (85.6, 86.3)	85.4 (85.0, 85.8)	-2.41 (-3.09, -1.72)	0.793	
Treated, %								
Women:	9.6 (8.8, 10.4)	10.1 (9.6, 10.5)	11.9 (11.5, 12.2)	13.9 (13.6, 14.2)	18.0 (17.7, 18.3)	8.31 (7.43, 9.20)	<0.001	<0.001
Men:	24.0 (23.0, 24.9)	22.5 (22.0, 23.0)	24.0 (23.6, 24.4)	26.7 (26.2, 27.1)	33.1 (32.6, 33.6)	9.11 (8.03, 10.2)	<0.001	
Controlled among treated, %								
Women:	22.8 (20.0, 25.7)	20.7 (19.3, 22.0)	23.3 (22.3, 24.3)	23.7 (22.9, 24.6)	25.3 (24.6, 26.0)	2.45 (-0.445, 5.35)	0.109	<0.001
Men:	41.1 (39.1, 43.1)	39.1 (38.0, 40.1)	39.0 (38.1, 39.8)	39.7 (38.9, 40.5)	39.3 (38.5, 40.0)	-1.83 (-4.01, 0.352)	0.219	
Diabetes, %								
Women:	0.73 (0.55, 0.91)	1.17 (1.05, 1.29)	1.50 (1.39, 1.61)	2.03 (1.92, 2.15)	3.06 (2.91, 3.20)	2.30 (2.07, 2.53)	0.104	0.079
Men:	2.09 (1.81, 2.36)	2.64 (2.47, 2.80)	3.04 (2.89, 3.19)	4.16 (3.98, 4.34)	6.03 (5.80, 6.25)	3.99 (3.63, 4.36)	<0.001	
Treated, %								
Women:	47.1 (39.4, 54.7)	54.5 (51.0, 58.0)	56.7 (54.1, 59.2)	61.2 (59.1, 63.3)	61.7 (60.0, 63.4)	14.6 (6.76, 22.5)	0.294	0.046
Men:	61.4 (56.6, 66.3)	62.3 (60.0, 64.5)	63.1 (61.3, 64.9)	63.8 (62.3, 65.4)	67.6 (66.2, 68.9)	6.14 (1.11, 11.2)	0.215	

Controlled among treated, %								
Women:	29.4 (20.0, 38.9)	27.8 (23.8, 31.8)	29.5 (26.7, 32.4)	27.2 (25.0, 29.5)	28.4 (26.5, 30.2)	-1.08 (-10.7, 8.57)	0.694	0.872
Men:	29.9 (24.4, 35.3)	28.4 (25.9, 30.9)	31.4 (29.4, 33.5)	30.4 (28.7, 32.2)	31.3 (29.7, 32.8)	1.37 (-4.30, 7.04)	0.457	
CKD, %								
Women:	0.95 (0.71, 1.20)	1.09 (0.96, 1.22)	1.22 (1.12, 1.33)	1.56 (1.46, 1.65)	1.96 (1.86, 2.07)	0.970 (0.711, 1.23)	0.398	0.257
Men:	0.89 (0.69, 1.09)	1.10 (0.98, 1.21)	1.24 (1.14, 1.34)	1.55 (1.45, 1.65)	2.21 (2.08, 2.33)	1.37 (1.12, 1.62)	0.013	
**Number of risk factors[c]**								
Number of risk factors								
Women:	1.22 (1.21, 1.24)	1.37 (1.36, 1.38)	1.48 (1.47, 1.49)	1.58 (1.58, 1.59)	1.73 (1.73, 1.74)	0.509 (0.490, 0.528)	<0.001	<0.001
Men:	1.57 (1.56, 1.59)	1.65 (1.64, 1.66)	1.71 (1.71, 1.72)	1.78 (1.77, 1.78)	1.93 (1.92, 1.93)	0.352 (0.334, 0.371)	<0.001	
Number of risk factors, %								
0								
Women:	17.8 (17.2, 18.5)	14.9 (14.6, 15.2)	13.0 (12.7, 13.3)	11.1 (10.8, 11.3)	9.12 (8.86, 9.4)	-8.87 (-9.59, -8.16)	0.066	<0.001
Men:	10.78 (10.28, 11.29)	9.49 (9.23, 9.74)	8.78 (8.55, 9.02)	8.13 (7.88, 8.39)	6.74 (6.47, 7.00)	-3.97 (-4.53, -3.40)	0.012	
1								
Women:	46.1 (45.1, 47.0)	42.8 (42.3, 43.3)	39.9 (39.5, 40.3)	37.0 (36.6, 37.4)	32.2 (31.8, 32.6)	-13.9 (-14.9, -12.9)	<0.001	0.366
Men:	37.8 (36.9, 38.6)	35.2 (34.8, 35.7)	32.7 (32.4, 33.1)	30.4 (30.0, 30.8)	25.5 (25.1, 26.0)	-12.2 (-13.2, -11.3)	<0.001	
2								
Women:	26.8 (25.9, 27.6)	30.9 (30.4, 31.3)	34.0 (33.6, 34.4)	36.4 (36.0, 36.7)	36.7 (36.3, 37.1)	9.94 (8.98, 10.9)	<0.001	<0.001
Men:	36.6 (35.7, 37.4)	39.0 (38.5, 39.4)	39.4 (39.0, 39.8)	39.1 (38.7, 39.5)	38.3 (37.8, 38.7)	1.70 (0.716, 2.69)	<0.001	
≥3								
Women:	6.4 (6.0, 6.9)	10.0 (9.7, 10.3)	12.8 (12.6, 13.1)	15.7 (15.4, 16.0)	21.4 (21.0, 21.7)	14.8 (14.3, 15.4)	<0.001	<0.001
Men:	14.9 (14.2, 15.5)	16.6 (16.3, 17.0)	19.2 (18.9, 19.5)	21.7 (21.3, 22.1)	27.9 (27.4, 28.3)	13.1 (12.3, 13.8)	<0.001	

Values are means for continuous variables adjusted for age, unless otherwise specified. Values between brackets indicate 95% confidence intervals, unless otherwise specified.

Household income groups: ≥£100,000; £52,000-<£100,000, £31,000-<£52,000; £18,000-<£31,000, and <£18,000 per year.

BMI = body mass index; CKD = chronic kidney disease; CRP = C-reactive protein; DBP = diastolic blood pressure; eGFR = estimated glomerular filtration rate; HbA1c = glycated haemoglobin; HDL-C = high-density lipoprotein cholesterol; LDL-C = low-density lipoprotein cholesterol; SBP = systolic blood pressure.

[a]*P* values for sex-specific linear trends across household income groups.

[b]*P* values for sex differences in linear trends across household income groups.

[c]Risk factors included were current smoking, hypertension, obesity, dyslipidaemia and diabetes.

Supplemental Table 7. Cardiometabolic risk factors, treatment, and control, by educational attainment.

	College/University Degree	Education to ≥18 years	Education to ≥16 years	No qualifications	Δ between lowest and highest education	*P*-Trend[a]
N	160,706	162,222	83,093	85,000	-	-
**Risk factors**						
SBP, mmHg	136.2 (136.1, 136.3)	138.2 (138.1, 138.3)	138.2 (138.1, 138.4)	139.4 (139.3, 139.6)	3.23 (3.07, 3.39)	<0.001
DBP, mmHg	81.6 (81.5, 81.6)	82.6 (82.5, 82.6)	82.4 (82.3, 82.4)	82.5 (82.4, 82.6)	0.944 (0.852, 1.03)	<0.001
BMI, kg/m2	26.6 (26.6, 26.6)	27.7 (27.7, 27.7)	27.6 (27.6, 27.6)	28.4 (28.4, 28.4)	1.79 (1.75, 1.83)	<0.001
Waist circumference, cm	88.7 (88.6, 88.7)	91.0 (90.9, 91.0)	89.7 (89.6, 89.8)	92.5 (92.4, 92.6)	3.82 (3.70, 3.93)	<0.001
Total cholesterol, mmol/L	5.70 (5.70, 5.71)	5.70 (5.69, 5.71)	5.75 (5.74, 5.76)	5.60 (5.60, 5.61)	-0.101 (-0.111, -0.091)	<0.001
LDL-C, mmol/L	3.55 (3.55, 3.56)	3.57 (3.56, 3.57)	3.59 (3.59, 3.60)	3.51 (3.50, 3.52)	-0.046 (-0.054, -0.038)	<0.001
HDL-C, mmol/L	1.48 (1.48, 1.49)	1.44 (1.43, 1.44)	1.47 (1.46, 1.47)	1.39 (1.39, 1.39)	-0.094 (-0.098, -0.091)	<0.001
Triglycerides, mmol/L	1.44 (1.44, 1.45)	1.55 (1.54, 1.55)	1.53 (1.52, 1.53)	1.61 (1.60, 1.62)	0.168 (0.161, 0.175)	<0.001
HbA1c, mmol/mol	35.2 (35.2, 35.2)	35.7 (35.6, 35.7)	35.7 (35.7, 35.7)	36.4 (36.4, 36.4)	1.23 (1.18, 1.27)	<0.001
Glucose, mmol/L	5.00 (4.99, 5.00)	5.03 (5.02, 5.03)	5.04 (5.03, 5.04)	5.09 (5.08, 5.09)	0.091 (0.083, 0.099)	<0.001
eGFR, mL/min/1.73m^2	94.9 (94.9, 95.0)	94.4 (94.3, 94.5)	94.5 (94.4, 94.6)	94.3 (94.2, 94.4)	-0.619 (-0.724, -0.515)	<0.001
CRP, mg/L	1.16 (1.15, 1.17)	1.42 (1.42, 1.43)	1.49 (1.48, 1.50)	1.75 (1.74, 1.77)	0.597 (0.582, 0.611)	<0.001
**Prevalence, treatment and control**						
Current smoking, %	7.05 (6.92, 7.17)	10.45 (10.30, 10.59)	11.38 (11.17, 11.60)	17.8 (17.5, 18.1)	10.8 (10.5, 11.1)	<0.001
Hypertension, %	51.2 (50.9, 51.4)	57.5 (57.2, 57.7)	57.6 (57.3, 58.0)	63.5 (63.1, 63.8)	12.3 (11.9, 12.7)	<0.001
Treated, %	36.0 (35.6, 36.3)	39.4 (39.0, 39.7)	38.9 (38.5, 39.4)	43.4 (43.0, 43.8)	7.47 (6.92, 8.01)	<0.001
Controlled among treated, %	40.2 (39.5, 40.8)	39.5 (39.0, 40.0)	39.6 (38.9, 40.3)	40.0 (39.4, 40.6)	-0.196 (-1.06, 0.669)	0.227
Obesity, %	18.7 (18.5, 18.9)	26.2 (26.0, 26.4)	25.8 (25.5, 26.1)	32.2 (31.8, 32.5)	13.4 (13.0, 13.8)	<0.001
Dyslipidaemia, %	86.3 (86.1, 86.4)	88.0 (87.8, 88.2)	88.5 (88.3, 88.7)	88.9 (88.6, 89.1)	2.62 (2.30, 2.93)	<0.001
Treated, %	17.4 (17.2, 17.7)	20.6 (20.4, 20.8)	19.5 (19.2, 19.9)	25.4 (25.1, 25.7)	8.01 (7.62, 8.41)	<0.001
Controlled among treated, %	33.3 (32.7, 33.8)	33.7 (33.2, 34.2)	32.6 (31.9, 33.4)	32.4 (31.8, 32.9)	-0.927 (-1.71, -0.141)	0.100
Diabetes, %	2.33 (2.26, 2.41)	2.98 (2.90, 3.07)	2.90 (2.78, 3.02)	4.15 (4.02, 4.28)	1.82 (1.66, 1.97)	<0.001
Treated, %	62.3 (61.0, 63.5)	62.0 (61.0, 63.1)	62.9 (61.4, 64.4)	64.0 (62.8, 65.1)	1.70 (-0.001, 3.40)	0.493
Controlled among treated, %	28.9 (27.5, 30.2)	30.0 (28.8, 31.2)	29.8 (28.2, 31.5)	30.2 (28.9, 31.4)	1.29 (-0.587, 3.16)	0.679
CKD, %	1.300 (1.239, 1.36)	1.62 (1.55, 1.68)	1.65 (1.56, 1.75)	2.11 (2.02, 2.19)	0.807 (0.701, 0.913)	<0.001
**Number of risk factors**[b]						
Number of risk factors	1.49 (1.48, 1.49)	1.68 (1.68, 1.69)	1.70 (1.69, 1.70)	1.86 (1.85, 1.86)	0.368 (0.361, 0.376)	<0.001
Number of risk factors, %						
0	12.9 (12.7, 13.0)	9.6 (9.5, 9.7)	9.2 (9.0, 9.4)	6.97 (6.77, 7.2)	-5.91 (-6.16, -5.65)	<0.001
1	39.4 (39.2, 39.6)	33.9 (33.7, 34.1)	33.4 (33.1, 33.8)	27.9 (27.6, 28.2)	-11.5 (-11.9, -11.1)	<0.001
2	34.6 (34.4, 34.8)	37.7 (37.5, 37.9)	38.5 (38.2, 38.8)	38.2 (37.9, 38.6)	3.62 (3.22, 4.03)	<0.001
≥3	12.8 (12.7, 13.0)	18.9 (18.7, 19.1)	18.9 (18.7, 19.2)	25.2 (24.9, 25.5)	12.3 (12.0, 12.7)	<0.001

Values are means for continuous variables adjusted for age, unless otherwise specified. Values between brackets indicate 95% confidence intervals, unless otherwise specified.

Educational attainment groups: Degree (University/College degree); ≥18 years (A/AS levels, NQV/HND/HNC or equivalent, other professional qualifications); ≥16 years (O levels/GCSEs or equivalent, CSEs or equivalent); and none (no qualifications).
BMI = body mass index; CKD = chronic kidney disease; CRP = C-reactive protein; DBP = diastolic blood pressure; eGFR = estimated glomerular filtration rate; HbA1c = glycated haemoglobin; HDL-C = high-density lipoprotein cholesterol; LDL-C = low-density lipoprotein cholesterol; SBP = systolic blood pressure.
[a] *P* values for sex-specific linear trends across educational attainment groups.
[b] Risk factors included were current smoking, hypertension, obesity, dyslipidaemia and diabetes.

Supplemental Table 8. Cardiometabolic risk factors, treatment, and control, by educational attainment and sex.

	College/University Degree	Education to ≥18 years	Education to ≥16 years	No qualifications	Δ between lowest and highest education	P-Trend[a]	P-Het[b]
N							
Women:	84,289	84,817	52,387	45,831	-	-	-
Men:	76,417	77,405	30,706	39,169	-	-	-
**Risk factors**							
SBP, mmHg							
Women:	133.3 (133.1, 133.4)	135.4 (135.2, 135.5)	136.3 (136.1, 136.4)	138.1 (137.9, 138.2)	4.81 (4.60, 5.02)	<0.001	<0.001
Men:	139.4 (139.3, 139.5)	141.3 (141.2, 141.4)	141.6 (141.4, 141.8)	141.1 (141.0, 141.3)	1.74 (1.51, 1.96)	<0.001	
DBP, mmHg							
Women:	79.9 (79.8, 80.0)	80.8 (80.7, 80.9)	81.1 (81.0, 81.2)	81.3 (81.2, 81.4)	1.36 (1.24, 1.48)	<0.001	<0.001
Men:	83.4 (83.3, 83.5)	84.5 (84.4, 84.5)	84.5 (84.3, 84.6)	84.1 (83.9, 84.2)	0.659 (0.530, 0.788)	<0.001	
BMI, kg/m^2							
Women:	26.2 (26.1, 26.2)	27.3 (27.3, 27.3)	27.3 (27.2, 27.3)	28.2 (28.2, 28.3)	2.04 (1.99, 2.10)	<0.001	<0.001
Men:	27.1 (27.0, 27.1)	28.1 (28.1, 28.1)	28.2 (28.1, 28.2)	28.6 (28.6, 28.7)	1.54 (1.48, 1.60)	<0.001	
Waist circumference, cm							
Women:	82.8 (82.7, 82.9)	85.1 (85.0, 85.2)	85.0 (84.9, 85.1)	87.2 (87.1, 87.4)	4.46 (4.32, 4.59)	<0.001	<0.001
Men:	95.1 (95.0, 95.2)	97.4 (97.3, 97.5)	97.8 (97.7, 97.9)	98.9 (98.7, 99.0)	3.73 (3.58, 3.87)	<0.001	
Total cholesterol, mmol/L							
Women:	5.86 (5.85, 5.86)	5.87 (5.87, 5.88)	5.89 (5.88, 5.90)	5.89 (5.88, 5.90)	0.036 (0.023, 0.049)	<0.001	<0.001
Men:	5.54 (5.53, 5.55)	5.51 (5.51, 5.52)	5.51 (5.50, 5.53)	5.27 (5.26, 5.28)	-0.275 (-0.289, -0.260)	<0.001	
LDL-C, mmol/L							
Women:	3.59 (3.58, 3.59)	3.63 (3.62, 3.63)	3.65 (3.64, 3.65)	3.67 (3.66, 3.68)	0.083 (0.072, 0.093)	<0.001	<0.001
Men:	3.52 (3.51, 3.53)	3.50 (3.50, 3.51)	3.50 (3.49, 3.51)	3.32 (3.31, 3.33)	-0.198 (0.083, 0.072)	<0.001	
HDL-C, mmol/L							
Women:	1.65 (1.65, 1.65)	1.59 (1.59, 1.59)	1.58 (1.58, 1.59)	1.52 (1.51, 1.52)	-0.130 (-0.135, -0.126)	<0.001	<0.001
Men:	1.31 (1.31, 1.31)	1.27 (1.27, 1.28)	1.27 (1.27, 1.28)	1.24 (1.23, 1.24)	-0.071 (-0.076, -0.066)	<0.001	
Triglycerides, mmol/L							
Women:	1.27 (1.27, 1.28)	1.38 (1.37, 1.38)	1.40 (1.39, 1.40)	1.54 (1.53, 1.55)	0.267 (0.258, 0.276)	<0.001	<0.001
Men:	1.66 (1.65, 1.66)	1.75 (1.75, 1.76)	1.77 (1.76, 1.78)	1.71 (1.70, 1.72)	0.051 (0.040, 0.062)	<0.001	
HbA1c, mmol/mol							
Women:	35.0 (35.0, 35.1)	35.4 (35.4, 35.5)	35.4 (35.4, 35.5)	36.1 (36.1, 36.2)	1.08 (1.02, 1.15)	<0.001	<0.001
Men:	35.3 (35.3, 35.4)	35.9 (35.9, 35.9)	36.2 (36.1, 36.2)	36.7 (36.7, 36.8)	1.41 (1.34, 1.48)	<0.001	
Glucose, mmol/L							
Women:	4.97 (4.96, 4.97)	4.99 (4.98, 5.00)	5.00 (4.99, 5.01)	5.04 (5.03, 5.05)	0.074 (0.063, 0.085)	0.080	<0.001
Men:	5.02 (5.02, 5.03)	5.07 (5.06, 5.07)	5.09 (5.08, 5.10)	5.14 (5.13, 5.15)	0.114 (0.102, 0.126)	0.054	
eGFR, mL/min/1.73m^2							

Women:	94.8 (94.7, 94.9)	94.3 (94.2, 94.4)	94.4 (94.3, 94.5)	94.0 (93.9, 94.1)	-0.839 (-0.981, -0.697)	<0.001	<0.001
Men:	95.1 (95.0, 95.2)	94.5 (94.4, 94.6)	94.7 (94.6, 94.8)	94.7 (94.6, 94.8)	-0.354 (-0.504, -0.205)	<0.001	
CRP, mg/L							
Women:	1.17 (1.166, 1.18)	1.47 (1.46, 1.48)	1.51 (1.50, 1.53)	1.84 (1.82, 1.86)	0.664 (0.644, 0.685)	<0.001	<0.001
Men:	1.14 (1.13, 1.15)	1.37 (1.36, 1.38)	1.45 (1.43, 1.46)	1.66 (1.64, 1.68)	0.520 (0.500, 0.540)	<0.001	
**Prevalence, treatment and control**							
Current smoking, %							
Women:	5.84 (5.68, 5.99)	8.66 (8.47, 8.84)	9.52 (9.27, 9.77)	15.46 (15.09, 15.82)	9.67 (9.28, 10.1)	<0.001	<0.001
Men:	8.43 (8.23, 8.62)	12.45 (12.22, 12.68)	14.5 (14.1, 14.9)	20.6 (20.2, 21.0)	12.1 (11.6, 12.6)	<0.001	
Hypertension, %							
Women:	44.5 (44.2, 44.9)	50.5 (50.2, 50.9)	52.4 (51.9, 52.8)	58.0 (57.5, 58.5)	13.5 (12.9, 14.1)	<0.001	<0.001
Men:	58.4 (58.1, 58.8)	65.2 (64.8, 65.5)	66.5 (66.0, 67.1)	69.9 (69.4, 70.4)	11.4 (10.8, 12.0)	<0.001	
Treated, %							
Women:	33.7 (33.1, 34.2)	38.1 (37.6, 38.6)	37.0 (36.4, 37.6)	41.4 (40.8, 41.9)	7.75 (6.97, 8.52)	<0.001	<0.001
Men:	37.8 (37.3, 38.3)	40.4 (40.0, 40.9)	41.7 (41.0, 42.4)	45.5 (44.9, 46.1)	7.68 (6.93, 8.42)	0.198	
Controlled among treated, %							
Women:	41.9 (41.0, 42.9)	41.4 (40.6, 42.3)	40.9 (39.9, 41.9)	40.8 (39.9, 41.6)	-1.18 (-2.48, 0.114)	0.586	0.139
Men:	38.9 (38.1, 39.7)	38.0 (37.3, 38.7)	38.0 (36.8, 39.1)	39.2 (38.3, 40.0)	0.254 (-0.896, 1.40)	0.150	
Obesity, %							
Women:	18.3 (18.0, 18.5)	25.0 (24.7, 25.3)	24.4 (24.1, 24.8)	31.3 (30.8, 31.7)	13.0 (12.5, 13.5)	<0.001	<0.001
Men:	19.3 (19.0, 19.5)	27.6 (27.3, 27.9)	28.0 (27.5, 28.5)	33.2 (32.7, 33.7)	14.0 (13.4, 14.5)	<0.001	
Dyslipidaemia, %							
Women:	86.2 (86.0, 86.5)	88.3 (88.0, 88.5)	88.9 (88.6, 89.2)	91.7 (91.4, 92.0)	5.53 (5.13, 5.93)	<0.001	<0.001
Men:	86.3 (86.0, 86.5)	87.7 (87.5, 87.9)	87.9 (87.5, 88.3)	85.8 (85.4, 86.2)	-0.471 (-0.949, 0.007)	<0.001	
Treated, %							
Women:	11.25 (10.97, 11.5)	14.3 (14.06, 14.6)	14.4 (14.1, 14.8)	19.8 (19.4, 20.2)	8.55 (8.08, 9.02)	<0.001	<0.001
Men:	23.6 (23.3, 24.0)	27.2 (26.9, 27.6)	28.7 (28.1, 29.3)	32.4 (31.9, 32.9)	8.73 (8.12, 9.35)	<0.001	
Controlled among treated, %							
Women:	22.8 (22.0, 23.6)	24.8 (24.1, 25.6)	24.8 (23.9, 25.8)	25.1 (24.4, 25.9)	2.32 (1.24, 3.40)	0.034	<0.001
Men:	40.0 (39.3, 40.8)	39.6 (38.9, 40.3)	41.3 (40.2, 42.4)	38.8 (38.0, 39.6)	-1.22 (-2.30, -0.138)	0.002	
Diabetes, %							
Women:	1.599 (1.508, 1.691)	2.03 (1.93, 2.13)	2.11 (1.98, 2.24)	3.07 (2.91, 3.22)	1.46 (1.28, 1.63)	<0.001	0.087
Men:	3.09 (2.97, 3.22)	3.99 (3.84, 4.13)	4.27 (4.04, 4.51)	5.46 (5.25, 5.68)	2.39 (2.13, 2.65)	0.002	
Treated, %							
Women:	59.2 (57.1, 61.3)	58.7 (56.9, 60.4)	59.8 (57.5, 62.0)	61.4 (59.6, 63.2)	2.21 (-0.574, 5.00)	0.579	0.892
Men:	64.0 (62.4, 65.5)	63.9 (62.6, 65.2)	65.6 (63.6, 67.6)	65.8 (64.3, 67.2)	1.80 (-0.314, 3.91)	0.666	
Controlled among treated, %							
Women:	27.2 (24.9, 29.4)	29.5 (27.6, 31.5)	27.1 (24.7, 29.6)	28.7 (26.7, 30.7)	1.494 (-1.53, 4.51)	0.175	0.264
Men:	29.8 (28.1, 31.5)	30.2 (28.7, 31.7)	32.0 (29.7, 34.3)	31.1 (29.5, 32.8)	1.364 (-1.00, 3.72)	0.528	

CKD, %							
Women:	1.314 (1.226, 1.40)	1.60 (1.51, 1.69)	1.56 (1.45, 1.67)	2.12 (2.01, 2.24)	0.791 (0.648, 0.934)	<0.001	0.064
Men:	1.288 (1.202, 1.37)	1.63 (1.54, 1.72)	1.83 (1.67, 1.99)	2.09 (1.97, 2.21)	0.823 (0.668, 0.977)	0.140	
**Number of risk factors[c]**							
Number of risk factors							
Women:	1.39 (1.39, 1.40)	1.57 (1.57, 1.58)	1.60 (1.60, 1.61)	1.78 (1.77, 1.79)	0.390 (0.379, 0.400)	<0.001	<0.001
Men:	1.59 (1.59, 1.60)	1.80 (1.80, 1.81)	1.85 (1.84, 1.86)	1.95 (1.94, 1.96)	0.354 (0.343, 0.366)	<0.001	
Number of risk factors, %							
0							
Women:	14.8 (14.6, 15.1)	11.4 (11.2, 11.6)	10.6 (10.4, 10.9)	7.8 (7.5, 8.1)	-7.09 (-7.46, -6.72)	<0.001	<0.001
Men:	10.6 (10.4, 10.8)	7.59 (7.41, 7.77)	6.84 (6.57, 7.11)	6.03 (5.76, 6.30)	-4.56 (-4.90, -4.22)	<0.001	
1							
Women:	42.1 (41.8, 42.5)	37.3 (37.0, 37.6)	36.2 (35.8, 36.6)	30.6 (30.2, 31.0)	-11.6 (-12.1, -11.0)	<0.001	<0.001
Men:	36.4 (36.0, 36.7)	30.2 (29.9, 30.5)	28.7 (28.2, 29.2)	24.7 (24.3, 25.1)	-11.6 (-12.2, -11.1)	<0.001	
2							
Women:	31.7 (31.4, 32.0)	35.7 (35.3, 36.0)	37.1 (36.7, 37.5)	37.9 (37.5, 38.4)	6.25 (5.71, 6.79)	<0.001	<0.001
Men:	37.8 (37.4, 38.1)	39.9 (39.6, 40.2)	40.9 (40.3, 41.4)	38.7 (38.2, 39.2)	0.900 (0.311, 1.49)	<0.001	
≥3							
Women:	10.64 (10.43, 10.85)	15.7 (15.41, 15.9)	16.0 (15.7, 16.4)	22.4 (22.1, 22.8)	11.8 (11.3, 12.2)	<0.001	<0.001
Men:	15.2 (14.9, 15.4)	22.4 (22.1, 22.7)	23.9 (23.4, 24.4)	28.4 (28.0, 28.9)	13.3 (12.8, 13.8)	<0.001	

Values are means for continuous variables adjusted for age, unless otherwise specified. Values between brackets indicate 95% confidence intervals, unless otherwise specified.

Educational attainment groups: Degree (University/College degree); ≥18 years (A/AS levels, NQV/HND/HNC or equivalent, other professional qualifications); ≥16 years (O levels/GCSEs or equivalent, CSEs or equivalent); and none (no qualifications).

BMI = body mass index; CKD = chronic kidney disease; CRP = C-reactive protein; DBP = diastolic blood pressure; eGFR = estimated glomerular filtration rate; HbA1c = glycated haemoglobin; HDL-C = high-density lipoprotein cholesterol; LDL-C = low-density lipoprotein cholesterol; SBP = systolic blood pressure.

[a]*P* values for sex-specific linear trends across educational attainment groups.

[b]*P* values for sex differences in linear trends across educational attainment groups.

[c]Risk factors included were current smoking, hypertension, obesity, dyslipidaemia and diabetes.

Supplemental Table 9. Cardiometabolic risk factors, treatment, and control, by Index of Multiple Deprivation fifths.

	1 (Least deprived)	2	3	4	5 (Most deprived)	Δ between most and least deprived	*P*-Trend[a]
N	97,837	97,651	97,674	97,669	97,315	-	-
**Risk factors**							
SBP, mmHg	137.4 (137.2, 137.5)	137.6 (137.5, 137.7)	137.9 (137.7, 138.0)	137.9 (137.8, 138.0)	138.3 (138.2, 138.4)	0.921 (0.756, 1.086)	0.114
DBP, mmHg	81.9 (81.9, 82.0)	82.1 (82.0, 82.1)	82.2 (82.2, 82.3)	82.3 (82.2, 82.3)	82.5 (82.4, 82.6)	0.571 (0.476, 0.666)	0.097
BMI, kg/m2	26.7 (26.7, 26.7)	27.0 (27.0, 27.0)	27.3 (27.3, 27.3)	27.7 (27.7, 27.7)	28.5 (28.5, 28.5)	1.82 (1.77, 1.86)	<0.001
Waist circumference, cm	88.6 (88.5, 88.6)	89.2 (89.1, 89.3)	89.9 (89.8, 89.9)	90.9 (90.9, 91.0)	93.0 (93.0, 93.1)	4.48 (4.36, 4.60)	<0.001
Total cholesterol, mmol/L	5.77 (5.76, 5.78)	5.74 (5.73, 5.75)	5.72 (5.71, 5.72)	5.67 (5.66, 5.68)	5.57 (5.57, 5.58)	-0.196 (-0.206, -0.185)	<0.001
LDL-C, mmol/L	3.60 (3.60, 3.61)	3.58 (3.58, 3.59)	3.57 (3.56, 3.58)	3.54 (3.54, 3.55)	3.49 (3.48, 3.49)	-0.117 (-0.125, -0.109)	<0.001
HDL-C, mmol/L	1.49 (1.48, 1.49)	1.47 (1.47, 1.48)	1.46 (1.46, 1.46)	1.43 (1.43, 1.44)	1.39 (1.39, 1.39)	-0.098 (-0.101, -0.094)	<0.001
Triglycerides, mmol/L	1.47 (1.46, 1.47)	1.49 (1.48, 1.49)	1.51 (1.51, 1.52)	1.54 (1.54, 1.55)	1.59 (1.59, 1.60)	0.129 (0.122, 0.136)	<0.001
HbA1c, mmol/mol	35.1 (35.1, 35.1)	35.3 (35.2, 35.3)	35.5 (35.4, 35.5)	35.8 (35.8, 35.9)	36.6 (36.6, 36.7)	1.54 (1.49, 1.59)	<0.001
Glucose, mmol/L	4.97 (4.96, 4.98)	5.00 (5.00, 5.01)	5.02 (5.01, 5.02)	5.05 (5.05, 5.06)	5.11 (5.10, 5.12)	0.139 (0.131, 0.148)	<0.001
eGFR, mL/min/1.73m^2	94.7 (94.6, 94.8)	94.6 (94.5, 94.7)	94.5 (94.4, 94.6)	94.5 (94.5, 94.6)	94.4 (94.3, 94.5)	-0.312 (-0.421, -0.203)	0.311
CRP, mg/L	1.22 (1.21, 1.23)	1.28 (1.27, 1.29)	1.36 (1.35, 1.37)	1.46 (1.45, 1.47)	1.71 (1.70, 1.72)	0.495 (0.480, 0.509)	<0.001
**Prevalence, treatment and control**							
Current smoking, %	6.30 (6.15, 6.46)	7.51 (7.34, 7.68)	8.99 (8.81, 9.17)	11.6 (11.4, 11.8)	18.1 (17.9, 18.4)	11.8 (11.5, 12.1)	<0.001
Hypertension, %	53.1 (52.8, 53.4)	54.5 (54.2, 54.8)	55.8 (55.5, 56.1)	57.6 (57.3, 57.9)	61.4 (61.1, 61.7)	8.24 (7.81, 8.68)	<0.001
Treated, %	35.0 (34.6, 35.4)	36.7 (36.3, 37.1)	37.9 (37.5, 38.3)	41.1 (40.7, 41.5)	45.7 (45.3, 46.2)	10.8 (10.2, 11.4)	<0.001
Controlled among treated, %	38.6 (37.8, 39.3)	39.1 (38.4, 39.8)	39.2 (38.5, 39.9)	39.3 (38.7, 40.0)	41.9 (41.2, 42.5)	3.32 (2.35, 4.29)	<0.001
Obesity, %	19.1 (18.9, 19.4)	21.4 (21.2, 21.7)	23.6 (23.3, 23.9)	26.8 (26.5, 27.1)	33.2 (32.9, 33.5)	14.1 (13.7, 14.5)	<0.001
Dyslipidaemia, %	87.1 (86.8, 87.3)	87.4 (87.2, 87.6)	87.7 (87.5, 87.9)	87.9 (87.7, 88.1)	88.1 (87.9, 88.3)	1.02 (0.709, 1.33)	0.886
Treated, %	16.4 (16.2, 16.7)	18.1 (17.8, 18.4)	19.4 (19.1, 19.7)	22.2 (21.9, 22.5)	27.6 (27.2, 27.9)	11.1 (10.7, 11.6)	<0.001
Controlled among treated, %	28.9 (28.3, 29.5)	33.3 (32.7, 34.0)	33.4 (32.8, 34.1)	33.9 (33.3, 34.5)	34.9 (34.3, 35.5)	5.93 (5.06, 6.80)	<0.001
Diabetes, %	2.02 (1.93, 2.11)	2.32 (2.22, 2.41)	2.64 (2.53, 2.74)	3.48 (3.36, 3.60)	4.98 (4.83, 5.12)	2.96 (2.79, 3.13)	<0.001
Treated, %	59.8 (58.1, 61.4)	59.9 (58.4, 61.5)	62.7 (61.3, 64.1)	63.5 (62.2, 64.7)	65.2 (64.1, 66.3)	5.43 (3.46, 7.39)	0.557
Controlled among treated, %	28.3 (26.5, 30.0)	29.1 (27.4, 30.8)	30.9 (29.3, 32.5)	29.1 (27.7, 30.5)	30.3 (29.1, 31.5)	2.04 (-0.103, 4.19)	0.207
CKD, %	1.31 (1.24, 1.38)	1.40 (1.33, 1.48)	1.58 (1.50, 1.66)	1.83 (1.74, 1.92)	2.26 (2.16, 2.36)	0.946 (0.823, 1.07)	0.009
**Number of risk factors[b]**							
Number of risk factors	1.49 (1.49, 1.50)	1.56 (1.56, 1.57)	1.61 (1.61, 1.62)	1.69 (1.69, 1.70)	1.87 (1.86, 1.87)	0.373 (0.365, 0.381)	<0.001
Number of risk factors, %							
0	12.8 (12.6, 13.0)	11.8 (11.6, 12.0)	10.9 (10.7, 11.1)	10.1 (9.9, 10.2)	7.93 (7.76, 8.09)	-4.85 (-5.11, -4.58)	<0.001
1	39.2 (38.9, 39.5)	37.0 (36.7, 37.3)	35.5 (35.2, 35.8)	33.2 (32.9, 33.4)	28.2 (28.0, 28.5)	-11.0 (-11.4, -10.6)	<0.001
2	35.5 (35.2, 35.8)	36.4 (36.1, 36.7)	36.9 (36.6, 37.2)	36.9 (36.6, 37.2)	38.1 (37.8, 38.4)	2.52 (2.10, 2.95)	0.003

≥3	12.7 (12.5, 12.9)	14.9 (14.7, 15.2)	16.7 (16.5, 16.9)	19.9 (19.7, 20.2)	26.1 (25.8, 26.4)	13.4 (13.1, 13.8)	<0.001

Values are means for continuous variables adjusted for age, unless otherwise specified. Values between brackets indicate 95% confidence intervals, unless otherwise specified.

Index of Multiple Deprivation fifths: ranging from 1 (least deprived) to 5 (most deprived).

BMI = body mass index; CKD = chronic kidney disease; CRP = C-reactive protein; DBP = diastolic blood pressure; eGFR = estimated glomerular filtration rate; HbA1c = glycated haemoglobin; HDL-C = high-density lipoprotein cholesterol; LDL-C = low-density lipoprotein cholesterol; SBP = systolic blood pressure.

[a] *P* values for sex-specific linear trends across Index of Multiple Deprivation fifths.

[b] Risk factors included were current smoking, hypertension, obesity, dyslipidaemia and diabetes.

Supplemental Table 10. Cardiometabolic risk factors, treatment, and control, by Index of Multiple Deprivation fifths and sex.

Risk factors	1 (Least deprived)	2	3	4	5 (Most deprived)	Δ between most and least deprived	P-Trend[a]	P-Het[b]
N								
Women:	53,791	53,727	53,741	53,470	51,127	-	-	-
Men:	44,046	43,924	43,933	44,199	46,188	-	-	-
**Risk factors**								
SBP, mmHg								
Women:	135.0 (134.9, 135.2)	135.3 (135.1, 135.5)	135.4 (135.3, 135.6)	135.5 (135.3, 135.6)	135.7 (135.5, 135.8)	0.644 (0.422, 0.867)	0.561	0.298
Men:	140.2 (140.0, 140.4)	140.5 (140.3, 140.6)	140.8 (140.6, 141.0)	140.8 (140.7, 141.0)	141.1 (140.9, 141.3)	0.931 (0.691, 1.17)	0.053	
DBP, mmHg								
Women:	80.4 (80.3, 80.5)	80.5 (80.4, 80.6)	80.6 (80.6, 80.7)	80.8 (80.7, 80.9)	81.0 (80.9, 81.1)	0.601 (0.474, 0.729)	0.218	<0.001
Men:	83.8 (83.7, 83.9)	84.0 (83.9, 84.1)	84.1 (84.0, 84.2)	84.0 (83.9, 84.1)	84.1 (84.0, 84.2)	0.344 (0.206, 0.481)	0.003	
BMI, kg/m^2								
Women:	26.2 (26.2, 26.2)	26.5 (26.5, 26.6)	26.9 (26.9, 27.0)	27.5 (27.4, 27.5)	28.5 (28.5, 28.6)	2.33 (2.27, 2.39)	<0.001	<0.001
Men:	27.3 (27.2, 27.3)	27.6 (27.6, 27.6)	27.8 (27.8, 27.8)	28.0 (28.0, 28.1)	28.5 (28.4, 28.5)	1.18 (1.12, 1.25)	<0.001	
Waist circumference, cm								
Women:	82.7 (82.6, 82.8)	83.4 (83.3, 83.5)	84.2 (84.1, 84.3)	85.7 (85.6, 85.8)	88.1 (88.0, 88.2)	5.41 (5.27, 5.55)	<0.001	<0.001
Men:	95.7 (95.6, 95.9)	96.3 (96.2, 96.4)	96.8 (96.7, 96.9)	97.3 (97.2, 97.4)	98.5 (98.4, 98.6)	2.72 (2.56, 2.87)	<0.001	
Total cholesterol, mmol/L								
Women:	5.95 (5.94, 5.96)	5.92 (5.91, 5.93)	5.90 (5.89, 5.91)	5.85 (5.84, 5.86)	5.75 (5.74, 5.76)	-0.199 (-0.213, -0.185)	<0.001	0.001
Men:	5.55 (5.54, 5.56)	5.51 (5.50, 5.52)	5.50 (5.49, 5.51)	5.45 (5.44, 5.47)	5.38 (5.37, 5.39)	-0.169 (-0.185, -0.154)	<0.001	
LDL-C, mmol/L								
Women:	3.66 (3.65, 3.67)	3.65 (3.64, 3.66)	3.64 (3.63, 3.65)	3.61 (3.61, 3.62)	3.56 (3.55, 3.57)	-0.099 (-0.110, -0.088)	<0.001	0.004
Men:	3.53 (3.52, 3.54)	3.50 (3.49, 3.51)	3.49 (3.48, 3.50)	3.46 (3.45, 3.47)	3.40 (3.39, 3.41)	-0.129 (-0.099, -0.110)	<0.001	
HDL-C, mmol/L								
Women:	1.64 (1.64, 1.65)	1.62 (1.62, 1.63)	1.60 (1.60, 1.61)	1.57 (1.57, 1.57)	1.52 (1.52, 1.52)	-0.123 (-0.128, -0.118)	<0.001	<0.001
Men:	1.30 (1.30, 1.30)	1.29 (1.29, 1.30)	1.28 (1.28, 1.29)	1.27 (1.27, 1.27)	1.25 (1.25, 1.25)	-0.051 (-0.056, -0.047)	<0.001	
Triglycerides, mmol/L								
Women:	1.32 (1.31, 1.32)	1.34 (1.34, 1.35)	1.37 (1.36, 1.37)	1.40 (1.39, 1.41)	1.46 (1.45, 1.46)	0.141 (0.133, 0.150)	<0.001	<0.001
Men:	1.67 (1.66, 1.68)	1.68 (1.68, 1.69)	1.71 (1.70, 1.72)	1.74 (1.73, 1.74)	1.76 (1.75, 1.77)	0.088 (0.076, 0.100)	0.500	
HbA1c, mmol/mol								
Women:	35.0 (35.0, 35.1)	35.1 (35.1, 35.2)	35.3 (35.3, 35.3)	35.6 (35.5, 35.6)	36.3 (36.2, 36.3)	1.27 (1.20, 1.33)	<0.001	<0.001
Men:	35.2 (35.2, 35.3)	35.4 (35.4, 35.5)	35.7 (35.6, 35.7)	36.1 (36.1, 36.2)	37.1 (37.0, 37.1)	1.84 (1.76, 1.91)	<0.001	
Glucose, mmol/L								
Women:	4.95 (4.94, 4.96)	4.98 (4.98, 4.99)	4.98 (4.98, 4.99)	5.01 (5.00, 5.02)	5.05 (5.04, 5.06)	0.098 (0.087, 0.110)	<0.001	<0.001
Men:	4.99 (4.98, 5.00)	5.03 (5.02, 5.04)	5.05 (5.04, 5.06)	5.10 (5.09, 5.11)	5.17 (5.16, 5.18)	0.182 (0.169, 0.194)	<0.001	
eGFR, mL/min/1.73m^2								

Women:	94.8 (94.7, 94.9)	94.6 (94.5, 94.7)	94.4 (94.3, 94.5)	94.4 (94.3, 94.5)	93.9 (93.8, 94.0)	-0.872 (-1.02, -0.723)	0.003	<0.001
Men:	94.6 (94.5, 94.8)	94.6 (94.5, 94.7)	94.7 (94.6, 94.8)	94.7 (94.6, 94.9)	95.0 (94.8, 95.1)	0.308 (0.149, 0.468)	0.152	
CRP, mg/L								
Women:	1.25 (1.24, 1.26)	1.31 (1.29, 1.32)	1.40 (1.39, 1.41)	1.52 (1.50, 1.53)	1.80 (1.78, 1.81)	0.549 (0.529, 0.570)	<0.001	<0.001
Men:	1.19 (1.17, 1.20)	1.25 (1.23, 1.26)	1.30 (1.29, 1.32)	1.39 (1.38, 1.41)	1.62 (1.61, 1.64)	0.442 (0.422, 0.463)	<0.001	
**Prevalence, treatment and control**								
Current smoking, %								
Women:	5.09 (4.90, 5.27)	6.32 (6.11, 6.53)	7.47 (7.24, 7.69)	10.0 (9.7, 10.2)	15.7 (15.4, 16.0)	10.6 (10.3, 11.0)	<0.001	0.002
Men:	7.81 (7.56, 8.06)	9.0 (8.72, 9.3)	10.9 (10.6, 11.2)	13.6 (13.3, 13.9)	20.8 (20.4, 21.2)	12.9 (12.5, 13.4)	<0.001	
Hypertension, %								
Women:	47.3 (46.9, 47.7)	48.4 (48.0, 48.9)	49.8 (49.4, 50.2)	51.5 (51.1, 52.0)	55.3 (54.8, 55.7)	8.00 (7.39, 8.60)	<0.001	0.525
Men:	60.3 (59.8, 60.7)	62.0 (61.6, 62.5)	63.3 (62.8, 63.7)	64.9 (64.4, 65.3)	68.1 (67.7, 68.5)	7.75 (7.13, 8.37)	<0.001	
Treated, %								
Women:	32.7 (32.1, 33.3)	34.4 (33.8, 35.0)	35.9 (35.3, 36.5)	39.7 (39.1, 40.3)	45.2 (44.6, 45.8)	12.6 (11.7, 13.4)	<0.001	<0.001
Men:	37.1 (36.5, 37.7)	38.8 (38.2, 39.4)	39.8 (39.3, 40.4)	42.4 (41.8, 43.0)	46.2 (45.7, 46.8)	9.07 (8.25, 9.90)	<0.001	
Controlled among treated, %								
Women:	39.4 (38.3, 40.5)	40.3 (39.2, 41.3)	40.5 (39.4, 41.5)	41.0 (40.1, 42.0)	43.9 (42.9, 44.8)	4.42 (2.99, 5.86)	0.054	0.245
Men:	37.8 (36.8, 38.8)	38.0 (37.1, 39.0)	38.1 (37.2, 39.1)	37.8 (36.9, 38.7)	40.2 (39.3, 41.0)	2.34 (1.03, 3.66)	0.013	
Obesity, %								
Women:	17.7 (17.4, 18.0)	19.9 (19.6, 20.3)	22.5 (22.1, 22.8)	26.3 (25.9, 26.6)	34.0 (33.6, 34.4)	16.3 (15.7, 16.8)	<0.001	<0.001
Men:	20.9 (20.5, 21.3)	23.2 (22.8, 23.6)	25.0 (24.6, 25.4)	27.5 (27.1, 27.9)	32.4 (31.9, 32.8)	11.5 (10.9, 12.1)	<0.001	
Dyslipidaemia, %								
Women:	87.5 (87.2, 87.8)	88.1 (87.8, 88.4)	88.3 (88.0, 88.6)	88.4 (88.1, 88.7)	88.7 (88.4, 89.0)	1.24 (0.824, 1.66)	0.665	0.380
Men:	86.5 (86.2, 86.9)	86.6 (86.3, 86.9)	86.9 (86.6, 87.2)	87.3 (87.0, 87.6)	87.4 (87.1, 87.7)	0.841 (0.382, 1.30)	0.540	
Treated, %								
Women:	11.0 (10.6, 11.3)	12.4 (12.0, 12.7)	13.8 (13.4, 14.1)	16.7 (16.3, 17.0)	21.4 (21.0, 21.9)	10.5 (9.95, 11.0)	<0.001	<0.001
Men:	23.0 (22.5, 23.4)	25.0 (24.6, 25.5)	26.2 (25.7, 26.6)	28.9 (28.4, 29.4)	34.3 (33.8, 34.8)	11.3 (10.6, 12.0)	<0.001	
Controlled among treated, %								
Women:	19.0 (18.2, 19.8)	24.0 (23.1, 24.9)	24.5 (23.6, 25.4)	25.8 (25.0, 26.7)	28.0 (27.1, 28.8)	8.93 (7.74, 10.1)	<0.001	<0.001
Men:	36.7 (35.8, 37.6)	40.3 (39.4, 41.2)	40.4 (39.5, 41.3)	40.4 (39.6, 41.3)	40.3 (39.4, 41.1)	3.54 (2.32, 4.76)	<0.001	
Diabetes, %								
Women:	1.36 (1.26, 1.46)	1.60 (1.49, 1.71)	1.91 (1.79, 2.03)	2.43 (2.30, 2.57)	3.75 (3.58, 3.93)	2.37 (2.16, 2.57)	<0.001	0.004
Men:	2.78 (2.63, 2.94)	3.16 (3.00, 3.33)	3.50 (3.33, 3.68)	4.71 (4.50, 4.91)	6.29 (6.06, 6.52)	3.55 (3.26, 3.83)	<0.001	
Treated, %								
Women:	55.6 (52.9, 58.3)	56.5 (54.0, 59.0)	59.9 (57.6, 62.2)	59.9 (57.8, 61.9)	63.1 (61.4, 64.9)	7.51 (4.30, 10.7)	0.554	0.541
Men:	62.3 (60.3, 64.4)	62.1 (60.2, 64.0)	64.6 (62.8, 66.4)	65.7 (64.1, 67.3)	66.5 (65.2, 67.9)	4.18 (1.71, 6.66)	0.660	
Controlled among treated, %								
Women:	25.9 (23.1, 28.8)	25.1 (22.4, 27.8)	29.4 (26.9, 31.9)	28.7 (26.5, 31.0)	29.2 (27.3, 31.1)	3.23 (-0.224, 6.68)	0.310	0.138
Men:	29.6 (27.3, 31.8)	31.3 (29.1, 33.5)	31.8 (29.8, 33.9)	29.2 (27.5, 31.0)	31.0 (29.5, 32.6)	1.43 (-1.30, 4.16)	0.163	

CKD, %								
Women:	1.35 (1.25, 1.45)	1.37 (1.27, 1.47)	1.59 (1.48, 1.70)	1.78 (1.66, 1.89)	2.27 (2.13, 2.41)	0.896 (0.728, 1.06)	0.007	0.399
Men:	1.27 (1.16, 1.37)	1.45 (1.34, 1.56)	1.56 (1.45, 1.68)	1.89 (1.76, 2.02)	2.24 (2.10, 2.39)	1.01 (0.824, 1.19)	0.383	
**Number of risk factors**[c]								
Number of risk factors								
Women:	1.40 (1.40, 1.41)	1.47 (1.46, 1.48)	1.52 (1.51, 1.53)	1.60 (1.59, 1.61)	1.78 (1.77, 1.79)	0.375 (0.364, 0.386)	<0.001	0.014
Men:	1.60 (1.59, 1.61)	1.67 (1.66, 1.68)	1.73 (1.72, 1.74)	1.80 (1.80, 1.81)	1.96 (1.95, 1.97)	0.359 (0.347, 0.371)	<0.001	
Number of risk factors, %								
0								
Women:	14.5 (14.3, 14.8)	13.4 (13.1, 13.7)	12.4 (12.1, 12.7)	11.6 (11.3, 11.9)	9.31 (9.07, 9.6)	-5.28 (-5.66, -4.90)	<0.001	0.398
Men:	10.53 (10.24, 10.82)	9.71 (9.44, 9.99)	9.08 (8.81, 9.34)	8.15 (7.90, 8.40)	6.38 (6.17, 6.59)	-4.11 (-4.46, -3.75)	<0.001	
1								
Women:	41.9 (41.5, 42.3)	40.1 (39.7, 40.5)	38.6 (38.2, 39.0)	35.8 (35.4, 36.2)	30.4 (30.0, 30.8)	-11.5 (-12.1, -10.9)	<0.001	<0.001
Men:	35.9 (35.5, 36.4)	33.1 (32.7, 33.6)	31.6 (31.2, 32.0)	29.9 (29.5, 30.3)	25.9 (25.5, 26.3)	-10.0 (-10.6, -9.43)	<0.001	
2								
Women:	33.4 (33.0, 33.8)	34.1 (33.7, 34.5)	35.1 (34.7, 35.5)	35.4 (35.0, 35.8)	37.0 (36.6, 37.4)	3.61 (3.04, 4.18)	0.018	<0.001
Men:	38.1 (37.7, 38.6)	39.3 (38.8, 39.7)	39.2 (38.7, 39.6)	38.7 (38.2, 39.1)	39.2 (38.8, 39.6)	1.07 (0.434, 1.70)	0.002	
≥3								
Women:	10.4 (10.1, 10.6)	12.6 (12.3, 12.9)	14.0 (13.7, 14.3)	17.2 (16.9, 17.5)	23.5 (23.1, 23.9)	13.1 (12.6, 13.5)	<0.001	<0.001
Men:	15.4 (15.1, 15.8)	17.8 (17.4, 18.1)	20.0 (19.7, 20.4)	23.2 (22.8, 23.6)	28.9 (28.5, 29.4)	13.6 (13.0, 14.1)	<0.001	

Values are means for continuous variables adjusted for age, unless otherwise specified. Values between brackets indicate 95% confidence intervals, unless otherwise specified.

Index of Multiple Deprivation fifths: ranging from 1 (least deprived) to 5 (most deprived).

BMI = body mass index; CKD = chronic kidney disease; CRP = C-reactive protein; DBP = diastolic blood pressure; eGFR = estimated glomerular filtration rate; HbA1c = glycated haemoglobin; HDL-C = high-density lipoprotein cholesterol; LDL-C = low-density lipoprotein cholesterol; SBP = systolic blood pressure.

[a]*P* values for sex-specific linear trends across Index of Multiple Deprivation fifths.

[b]*P* values for sex differences in linear trends across Index of Multiple Deprivation fifths.

[c]Risk factors included were current smoking, hypertension, obesity, dyslipidaemia and diabetes.

Supplemental Table 11. Cardiometabolic risk factors, treatment, and control, weighted by National Townsend Deprivation Score fifths.

Risk factors	Townsend-weighted
SBP, mmHg	137.7 (137.6, 137.8)
DBP, mmHg	82.2 (82.1, 82.3)
BMI, kg/m^2	27.6 (27.6, 27.6)
Waist circumference, cm	90.7 (90.6, 90.8)
Total cholesterol, mmol/L	5.67 (5.66, 5.68)
LDL-C, mmol/L	3.54 (3.54, 3.55)
HDL-C, mmol/L	1.44 (1.44, 1.44)
Triglycerides, mmol/L	1.53 (1.52, 1.53)
HbA1c, mmol/mol	35.8 (35.8, 35.9)
Glucose, mmol/L	5.04 (5.04, 5.05)
eGFR, mL/min/1.73m^2	94.6 (94.5, 94.7)
CRP, mg/L	1.44 (1.43, 1.45)
**Prevalence, treatment and control**	
Current smoking, %	11.1 (10.9, 11.3)
Hypertension, %	57.0 (56.6, 57.3)
Treated, %	40.4 (40.0, 40.8)
Controlled among treated, %	40.1 (39.4, 40.9)
Obesity, %	25.8 (25.6, 26.1)
Dyslipidaemia, %	87.7 (87.4, 87.9)
Treated, %	21.6 (21.3, 21.9)
Controlled among treated, %	33.1 (32.4, 33.7)
Diabetes mellitus, %	3.26 (3.14, 3.38)
Treated, %	63.0 (61.7, 64.4)
Controlled among treated, %	29.7 (28.2, 31.2)
CKD, %	1.75 (1.66, 1.84)
**Number of risk factors[a]**	
Number of risk factors	1.68 (1.67, 1.68)
Number of risk factors, %	
0	10.2 (10.0, 10.4)
1	33.5 (33.2, 33.8)
2	36.8 (36.5, 37.1)
≥3	19.0 (18.7, 19.2)

Values are means for continuous variables adjusted for age, unless otherwise specified. Values between brackets indicate 95% confidence intervals, unless otherwise specified.
Educational attainment groups: University/College degree; (A/AS levels. NQV/HND/HNC or equivalent, other professional qualifications); (O levels/GCSEs or equivalent); and no qualifications.
BMI = body mass index; CKD = chronic kidney disease; CRP = C-reactive protein; DBP = diastolic blood pressure; eGFR = estimated glomerular filtration rate; HbA1c = glycated haemoglobin; HDL-C = high-density lipoprotein cholesterol; LDL-C = low-density lipoprotein cholesterol; SBP = systolic blood pressure.
[a]Risk factors included were current smoking, hypertension, obesity, dyslipidaemia and diabetes.

Supplemental Table 12. Cardiometabolic risk factors, treatment, and control, by household income, with further adjustment for number of people living in household.

	≥£100,000	£52,000-<£100,000	£31,000-<£52,000	£18,000-<£31,000	<£18,000	Δ between lowest and highest income	*P*-Trend[a]
N	22,875	86,037	110,495	107,927	96,882		-
**Risk factors**							
SBP, mmHg	135.2 (134.9, 135.4)	137.0 (136.9, 137.1)	137.7 (137.6, 137.8)	137.9 (137.8, 138.0)	137.6 (137.5, 137.7)	2.44 (2.17, 2.71)	<0.001
DBP, mmHg	81.2 (81.1, 81.4)	82.3 (82.2, 82.3)	82.5 (82.4, 82.5)	82.3 (82.2, 82.4)	82.0 (81.9, 82.1)	0.752 (0.596, 0.909)	<0.001
BMI, kg/m2	26.3 (26.3, 26.4)	26.9 (26.9, 26.9)	27.3 (27.3, 27.3)	27.5 (27.5, 27.6)	28.1 (28.1, 28.1)	1.76 (1.69, 1.83)	<0.001
Waist circumference, cm	88.6 (88.4, 88.7)	89.7 (89.6, 89.8)	90.2 (90.2, 90.3)	90.4 (90.3, 90.5)	91.6 (91.5, 91.7)	3.05 (2.85, 3.25)	<0.001
Total cholesterol, mmol/L	5.73 (5.71, 5.74)	5.74 (5.73, 5.74)	5.72 (5.71, 5.73)	5.69 (5.68, 5.69)	5.59 (5.58, 5.59)	-0.142 (-0.159, -0.124)	<0.001
LDL-C, mmol/L	3.56 (3.54, 3.57)	3.58 (3.58, 3.59)	3.58 (3.57, 3.58)	3.56 (3.55, 3.56)	3.49 (3.49, 3.50)	-0.063 (-0.076, -0.050)	<0.001
HDL-C, mmol/L	1.52 (1.52, 1.53)	1.48 (1.47, 1.48)	1.45 (1.45, 1.46)	1.44 (1.43, 1.44)	1.40 (1.39, 1.40)	-0.125 (-0.131, -0.119)	<0.001
Triglycerides, mmol/L	1.37 (1.36, 1.38)	1.47 (1.46, 1.47)	1.51 (1.50, 1.51)	1.54 (1.53, 1.54)	1.59 (1.59, 1.60)	0.220 (0.209, 0.231)	<0.001
HbA1c, mmol/mol	34.6 (34.6, 34.7)	34.9 (34.9, 35.0)	35.3 (35.3, 35.3)	35.7 (35.7, 35.7)	36.4 (36.4, 36.4)	1.78 (1.70, 1.85)	<0.001
Glucose, mmol/L	4.98 (4.97, 4.99)	4.98 (4.98, 4.99)	5.00 (4.99, 5.00)	5.03 (5.02, 5.03)	5.08 (5.07, 5.09)	0.102 (0.088, 0.116)	<0.001
eGFR, mL/min/1.73m^2	94.6 (94.4, 94.7)	94.8 (94.7, 94.9)	95.0 (95.0, 95.1)	94.9 (94.9, 95.0)	94.7 (94.6, 94.8)	0.146 (-0.034, 0.327)	<0.001
CRP, mg/L	1.05 (1.04, 1.07)	1.18 (1.18, 1.19)	1.30 (1.29, 1.31)	1.42 (1.41, 1.43)	1.68 (1.67, 1.70)	0.631 (0.611, 0.650)	<0.001
**Prevalence, treatment and control**							
Current smoking, %	5.99 (5.70, 6.29)	6.87 (6.71, 7.04)	8.70 (8.54, 8.86)	11.3 (11.1, 11.5)	17.4 (17.1, 17.6)	11.4 (11.0, 11.8)	<0.001
Hypertension, %	48.0 (47.4, 48.7)	52.4 (52.1, 52.7)	55.1 (54.8, 55.4)	56.8 (56.5, 57.1)	59.5 (59.2, 59.9)	11.5 (10.7, 12.2)	<0.001
Treated, %	33.5 (32.5, 34.5)	34.2 (33.7, 34.7)	36.5 (36.1, 36.9)	39.0 (38.6, 39.4)	43.9 (43.5, 44.3)	10.4 (9.30, 11.5)	<0.001
Controlled among treated, %	41.4 (39.4, 43.3)	39.0 (38.1, 40.0)	39.0 (38.3, 39.7)	39.3 (38.7, 39.9)	41.6 (41.0, 42.2)	0.206 (-1.82, 2.23)	<0.001
Obesity, %	16.3 (15.8, 16.8)	20.4 (20.1, 20.7)	23.2 (22.9, 23.4)	25.4 (25.1, 25.6)	30.4 (30.1, 30.7)	14.1 (13.5, 14.7)	<0.001
Dyslipidaemia, %	86.1 (85.7, 86.6)	86.9 (86.7, 87.1)	87.1 (86.9, 87.3)	87.5 (87.3, 87.8)	88.0 (87.7, 88.2)	1.85 (1.34, 2.37)	0.406
Treated, %	17.9 (17.2, 18.6)	16.9 (16.6, 17.3)	18.1 (17.8, 18.4)	19.8 (19.5, 20.1)	24.5 (24.2, 24.8)	6.54 (5.79, 7.29)	<0.001
Controlled among treated, %	36.0 (34.4, 37.7)	33.3 (32.5, 34.2)	33.2 (32.5, 33.9)	32.9 (32.4, 33.5)	32.6 (32.1, 33.2)	-3.43 (-5.22, -1.64)	0.109
Diabetes, %	1.46 (1.29, 1.63)	1.95 (1.85, 2.05)	2.28 (2.18, 2.37)	3.02 (2.92, 3.12)	4.36 (4.23, 4.49)	2.90 (2.68, 3.12)	<0.001
Treated, %	57.9 (53.8, 62.0)	60.4 (58.5, 62.3)	61.1 (59.6, 62.6)	62.9 (61.6, 64.1)	64.9 (63.8, 66.0)	7.00 (2.71, 11.3)	0.813
Controlled among treated, %	29.9 (25.2, 34.7)	28.4 (26.2, 30.5)	30.9 (29.2, 32.6)	29.3 (27.9, 30.7)	30.1 (28.9, 31.2)	0.104 (-4.83, 5.04)	0.276
CKD, %	0.90 (0.75, 1.06)	1.08 (1.00, 1.17)	1.23 (1.16, 1.30)	1.56 (1.48, 1.63)	2.09 (2.00, 2.17)	1.18 (1.00, 1.36)	0.013
Number of risk factors[b]							
Number of risk factors	1.42 (1.41, 1.43)	1.52 (1.52, 1.53)	1.60 (1.59, 1.60)	1.67 (1.66, 1.67)	1.81 (1.80, 1.81)	0.387 (0.373, 0.401)	<0.001
Number of risk factors, %							
0	14.0 (13.6, 14.4)	12.1 (11.9, 12.3)	11.0 (10.8, 11.2)	9.84 (9.65, 10.0)	8.21 (8.02, 8.41)	-5.78 (-6.24, -5.32)	<0.001
1	41.5 (40.8, 42.1)	38.7 (38.4, 39.1)	36.4 (36.1, 36.7)	34.1 (33.8, 34.3)	29.5 (29.2, 29.8)	-12.0 (-12.7, -11.26)	<0.001

2	32.2 (31.6, 32.8)	35.2 (34.9, 35.6)	36.7 (36.4, 37.0)	37.5 (37.3, 37.8)	37.3 (37.0, 37.6)	5.08 (4.37, 5.78)	<0.001
≥3	11.2 (10.8, 11.6)	13.7 (13.4, 13.9)	16.0 (15.8, 16.3)	18.3 (18.1, 18.6)	23.8 (23.6, 24.1)	12.6 (12.1, 13.2)	<0.001

Values are means for continuous variables adjusted for age, unless otherwise specified. Values between brackets indicate 95% confidence intervals, unless otherwise specified. Models further adjusted for number living in participant household.

Household income groups: ≥£100,000; £52,000-<£100,000, £31,000-<£52,000; £18,000-<£31,000, and <£18,000 per year.

BMI = body mass index; CKD = chronic kidney disease; CRP = C-reactive protein; DBP = diastolic blood pressure; eGFR = estimated glomerular filtration rate; HbA1c = glycated haemoglobin; HDL-C = high-density lipoprotein cholesterol; LDL-C = low-density lipoprotein cholesterol; SBP = systolic blood pressure.

[a]*P* values for sex-specific linear trends across household income groups.

[b]Risk factors included were current smoking, hypertension, obesity, dyslipidaemia and diabetes.

Supplemental Table 13. Cardiometabolic risk factors, treatment, and control, by household income and sex, with further adjustment for number of people living in household.

Risk factors	≥£100,000	£52,000-<£100,000	£31,000-<£52,000	£18,000-<£31,000	<£18,000	Δ between lowest and highest income	P-Trend[a]	P-Het[b]
N								
Women:	10,699	41,525	56,609	58,697	55,244	-	-	-
Men:	12,176	44,512	53,886	49,230	41,638	-	-	-
**Risk factors**								
SBP, mmHg								
Women:	130.9 (130.5, 131.2)	133.3 (133.2, 133.5)	134.5 (134.4, 134.7)	135.7 (135.6, 135.9)	135.8 (135.7, 136.0)	4.95 (4.57, 5.32)	<0.001	<0.001
Men:	138.8 (138.5, 139.2)	140.3 (140.1, 140.4)	140.9 (140.8, 141.1)	140.6 (140.4, 140.8)	140.1 (139.9, 140.2)	1.24 (0.871, 1.61)	<0.001	
DBP, mmHg								
Women:	78.8 (78.6, 79.0)	80.1 (80.0, 80.2)	80.6 (80.6, 80.7)	80.9 (80.8, 81.0)	80.8 (80.7, 80.9)	2.03 (1.81, 2.25)	<0.001	<0.001
Men:	83.4 (83.2, 83.5)	84.2 (84.1, 84.3)	84.4 (84.3, 84.5)	84.0 (83.9, 84.1)	83.6 (83.5, 83.7)	0.281 (0.068, 0.493)	<0.001	
BMI, kg/m^2								
Women:	25.2 (25.1, 25.3)	26.2 (26.1, 26.2)	26.8 (26.8, 26.9)	27.3 (27.2, 27.3)	28.0 (28.0, 28.1)	2.83 (2.73, 2.93)	<0.001	<0.001
Men:	27.3 (27.2, 27.4)	27.6 (27.6, 27.6)	27.8 (27.7, 27.8)	27.9 (27.8, 27.9)	28.2 (28.2, 28.3)	0.910 (0.813, 1.01)	<0.001	
Waist circumference, cm								
Women:	80.4 (80.2, 80.7)	82.5 (82.4, 82.7)	84.1 (84.0, 84.2)	85.1 (85.0, 85.2)	86.9 (86.8, 87.0)	6.46 (6.21, 6.71)	<0.001	<0.001
Men:	95.5 (95.3, 95.7)	96.2 (96.1, 96.3)	96.6 (96.5, 96.7)	96.9 (96.8, 97.0)	98.1 (98.0, 98.2)	2.64 (2.40, 2.88)	<0.001	
Total cholesterol, mmol/L								
Women:	5.78 (5.76, 5.81)	5.82 (5.81, 5.83)	5.87 (5.86, 5.88)	5.92 (5.91, 5.93)	5.85 (5.84, 5.86)	0.062 (0.038, 0.086)	<0.001	<0.001
Men:	5.68 (5.66, 5.70)	5.66 (5.65, 5.67)	5.56 (5.56, 5.57)	5.41 (5.40, 5.42)	5.24 (5.23, 5.25)	-0.443 (-0.467, -0.420)	<0.001	
LDL-C, mmol/L								
Women:	3.49 (3.47, 3.51)	3.56 (3.55, 3.56)	3.62 (3.61, 3.62)	3.67 (3.66, 3.68)	3.64 (3.63, 3.64)	0.148 (0.129, 0.166)	<0.001	<0.001
Men:	3.61 (3.60, 3.63)	3.61 (3.60, 3.62)	3.54 (3.53, 3.55)	3.43 (3.42, 3.44)	3.30 (3.29, 3.31)	-0.312 (0.148, 0.129)	<0.001	
HDL-C, mmol/L								
Women:	1.73 (1.72, 1.74)	1.66 (1.66, 1.67)	1.62 (1.61, 1.62)	1.58 (1.58, 1.58)	1.52 (1.51, 1.52)	-0.210 (-0.218, -0.202)	<0.001	<0.001
Men:	1.35 (1.34, 1.36)	1.31 (1.31, 1.32)	1.29 (1.29, 1.29)	1.27 (1.26, 1.27)	1.23 (1.23, 1.23)	-0.120 (-0.127, -0.112)	<0.001	
Triglycerides, mmol/L								
Women:	1.13 (1.12, 1.14)	1.24 (1.23, 1.24)	1.32 (1.31, 1.32)	1.41 (1.41, 1.42)	1.50 (1.50, 1.51)	0.376 (0.363, 0.389)	<0.001	<0.001
Men:	1.62 (1.61, 1.64)	1.71 (1.71, 1.72)	1.72 (1.72, 1.73)	1.70 (1.69, 1.71)	1.73 (1.72, 1.74)	0.107 (0.089, 0.124)	<0.001	
HbA1c, mmol/mol								
Women:	34.3 (34.2, 34.4)	34.6 (34.6, 34.7)	35.1 (35.0, 35.1)	35.5 (35.4, 35.5)	36.1 (36.0, 36.1)	1.76 (1.65, 1.87)	<0.001	<0.001
Men:	34.9 (34.8, 35.0)	35.2 (35.1, 35.2)	35.5 (35.5, 35.6)	36.0 (35.9, 36.0)	36.8 (36.8, 36.9)	1.94 (1.83, 2.05)	<0.001	
Glucose, mmol/L								
Women:	4.94 (4.92, 4.96)	4.95 (4.94, 4.96)	4.96 (4.95, 4.97)	4.99 (4.98, 5.00)	5.03 (5.02, 5.04)	0.090 (0.071, 0.110)	<0.001	<0.001
Men:	5.01 (4.99, 5.03)	5.01 (5.01, 5.02)	5.03 (5.02, 5.04)	5.07 (5.06, 5.08)	5.15 (5.14, 5.16)	0.137 (0.118, 0.156)	<0.001	
eGFR, mL/min/1.73m^2								

Women:	94.6 (94.4, 94.8)	94.9 (94.8, 95.0)	94.9 (94.8, 95.0)	94.7 (94.6, 94.8)	94.4 (94.3, 94.5)	-0.186 (-0.441, 0.070)	<0.001	<0.001
Men:	94.6 (94.4, 94.8)	94.8 (94.6, 94.9)	95.2 (95.1, 95.3)	95.2 (95.1, 95.3)	95.2 (95.0, 95.3)	0.583 (0.335, 0.830)	<0.001	
CRP, mg/L								
Women:	1.01 (0.99, 1.03)	1.18 (1.17, 1.19)	1.34 (1.33, 1.35)	1.47 (1.46, 1.48)	1.72 (1.71, 1.74)	0.712 (0.686, 0.738)	<0.001	<0.001
Men:	1.10 (1.08, 1.12)	1.19 (1.18, 1.20)	1.26 (1.25, 1.27)	1.36 (1.34, 1.37)	1.63 (1.62, 1.65)	0.542 (0.514, 0.569)	<0.001	
**Prevalence, treatment and control**								
Current smoking, %								
Women:	4.40 (4.04, 4.77)	5.34 (5.14, 5.55)	7.15 (6.95, 7.36)	9.4 (9.2, 9.7)	14.7 (14.3, 15.0)	10.4 (9.95, 10.9)	<0.001	0.043
Men:	7.46 (7.00, 7.91)	8.4 (8.11, 8.6)	10.4 (10.1, 10.7)	13.7 (13.4, 14.0)	21.1 (20.7, 21.6)	13.4 (12.8, 14.0)	<0.001	
Hypertension, %								
Women:	38.3 (37.3, 39.3)	44.4 (43.9, 44.8)	47.7 (47.3, 48.1)	50.7 (50.3, 51.1)	53.8 (53.3, 54.2)	15.45 (14.38, 16.51)	<0.001	<0.001
Men:	56.2 (55.3, 57.0)	59.7 (59.2, 60.1)	62.8 (62.4, 63.2)	64.4 (64.0, 64.8)	67.5 (67.0, 68.0)	11.21 (10.22, 12.20)	0.025	
Treated, %								
Women:	32.1 (30.3, 33.8)	31.8 (31.0, 32.6)	35.1 (34.5, 35.7)	36.8 (36.3, 37.4)	41.2 (40.6, 41.7)	9.12 (7.30, 10.9)	<0.001	<0.001
Men:	34.3 (33.0, 35.5)	35.7 (35.1, 36.4)	37.5 (37.0, 38.1)	41.0 (40.5, 41.6)	46.8 (46.2, 47.4)	12.5 (11.1, 13.9)	<0.001	
Controlled among treated, %								
Women:	45.1 (41.7, 48.5)	42.2 (40.6, 43.8)	41.7 (40.5, 42.8)	40.2 (39.3, 41.2)	43.0 (42.1, 43.8)	-2.16 (-5.68, 1.35)	<0.001	0.011
Men:	39.6 (37.3, 41.9)	37.4 (36.3, 38.5)	37.2 (36.3, 38.1)	38.5 (37.7, 39.4)	40.2 (39.4, 41.1)	0.639 (-1.82, 3.10)	0.004	
Obesity, %								
Women:	12.0 (11.4, 12.6)	17.8 (17.4, 18.1)	21.8 (21.5, 22.2)	24.8 (24.4, 25.1)	30.3 (29.9, 30.7)	18.4 (17.6, 19.1)	<0.001	<0.001
Men:	20.0 (19.3, 20.7)	22.8 (22.5, 23.2)	24.6 (24.2, 24.9)	26.2 (25.8, 26.5)	30.6 (30.1, 31.1)	10.6 (9.71, 11.4)	<0.001	
Dyslipidaemia, %								
Women:	84.1 (83.4, 84.8)	85.8 (85.5, 86.1)	87.1 (86.8, 87.4)	88.9 (88.6, 89.2)	90.0 (89.7, 90.3)	5.96 (5.20, 6.71)	0.452	<0.001
Men:	87.9 (87.4, 88.5)	87.9 (87.6, 88.2)	87.1 (86.8, 87.3)	85.9 (85.6, 86.2)	85.3 (84.9, 85.7)	-2.56 (-3.24, -1.87)	0.803	
Treated, %								
Women:	9.6 (8.7, 10.4)	10.0 (9.6, 10.4)	11.9 (11.5, 12.2)	13.9 (13.6, 14.2)	18.1 (17.7, 18.4)	8.40 (7.51, 9.29)	<0.001	<0.001
Men:	23.9 (22.9, 24.9)	22.4 (21.9, 22.9)	24.0 (23.5, 24.4)	26.7 (26.2, 27.1)	33.1 (32.6, 33.6)	9.25 (8.15, 10.3)	<0.001	
Controlled among treated, %								
Women:	22.9 (20.0, 25.7)	20.7 (19.4, 22.0)	23.3 (22.3, 24.3)	23.7 (22.9, 24.5)	25.3 (24.5, 26.0)	2.38 (-0.530, 5.29)	0.111	<0.001
Men:	41.1 (39.1, 43.2)	39.1 (38.1, 40.2)	39.0 (38.1, 39.9)	39.7 (38.9, 40.5)	39.2 (38.5, 40.0)	-1.92 (-4.11, 0.277)	0.218	
Diabetes, %								
Women:	0.72 (0.54, 0.90)	1.16 (1.05, 1.28)	1.50 (1.39, 1.61)	2.04 (1.92, 2.16)	3.09 (2.94, 3.24)	2.34 (2.11, 2.57)	0.103	0.071
Men:	2.06 (1.78, 2.33)	2.61 (2.45, 2.77)	3.03 (2.88, 3.18)	4.16 (3.99, 4.34)	6.07 (5.83, 6.30)	4.07 (3.71, 4.44)	<0.001	
Treated, %								
Women:	47.9 (40.2, 55.6)	54.9 (51.4, 58.4)	56.8 (54.3, 59.4)	61.2 (59.1, 63.3)	61.4 (59.7, 63.1)	13.5 (5.61, 21.4)	0.285	0.051
Men:	61.9 (57.1, 66.7)	62.6 (60.4, 64.9)	63.2 (61.5, 65.0)	63.8 (62.3, 65.4)	67.4 (66.0, 68.7)	5.49 (0.468, 10.5)	0.217	
Controlled among treated, %								
Women:	29.6 (20.1, 39.1)	27.9 (23.9, 31.9)	29.6 (26.7, 32.5)	27.2 (24.9, 29.5)	28.3 (26.4, 30.1)	-1.32 (-11.0, 8.38)	0.662	0.869
Men:	30.0 (24.6, 35.5)	28.5 (26.0, 31.0)	31.5 (29.5, 33.5)	30.5 (28.7, 32.2)	31.2 (29.7, 32.7)	1.17 (-4.54, 6.88)	0.466	

CKD, %								
Women:	0.94 (0.70, 1.18)	1.08 (0.95, 1.21)	1.22 (1.11, 1.32)	1.56 (1.46, 1.66)	1.98 (1.87, 2.09)	1.00 (0.743, 1.26)	0.401	0.277
Men:	0.88 (0.68, 1.08)	1.09 (0.97, 1.20)	1.24 (1.14, 1.33)	1.55 (1.45, 1.65)	2.22 (2.09, 2.34)	1.40 (1.15, 1.65)	0.013	
**Number of risk factors[c]**								
Number of risk factors								
Women:	1.23 (1.22, 1.25)	1.37 (1.37, 1.38)	1.48 (1.47, 1.49)	1.58 (1.57, 1.59)	1.73 (1.72, 1.73)	0.494 (0.474, 0.513)	<0.001	<0.001
Men:	1.58 (1.57, 1.60)	1.65 (1.65, 1.66)	1.72 (1.71, 1.72)	1.77 (1.77, 1.78)	1.92 (1.91, 1.93)	0.339 (0.321, 0.358)	<0.001	
Number of risk factors, %								
0								
Women:	17.6 (17.0, 18.3)	14.8 (14.5, 15.1)	13.0 (12.7, 13.3)	11.1 (10.9, 11.4)	9.22 (8.95, 9.5)	-8.56 (-9.28, -7.84)	0.061	<0.001
Men:	10.66 (10.16, 11.16)	9.41 (9.16, 9.66)	8.77 (8.54, 9.00)	8.16 (7.90, 8.42)	6.80 (6.53, 7.07)	-3.79 (-4.35, -3.23)	0.012	
1								
Women:	45.9 (45.0, 46.9)	42.7 (42.3, 43.2)	39.9 (39.5, 40.3)	37.0 (36.6, 37.4)	32.3 (31.9, 32.7)	-13.6 (-14.7, -12.6)	<0.001	0.381
Men:	37.6 (36.8, 38.5)	35.1 (34.7, 35.6)	32.7 (32.3, 33.1)	30.4 (30.0, 30.8)	25.6 (25.2, 26.0)	-12.0 (-13.0, -11.1)	<0.001	
2								
Women:	26.8 (26.0, 27.7)	30.9 (30.5, 31.4)	34.0 (33.6, 34.4)	36.3 (35.9, 36.7)	36.7 (36.3, 37.1)	9.79 (8.82, 10.8)	<0.001	<0.001
Men:	36.7 (35.8, 37.5)	39.0 (38.6, 39.5)	39.4 (39.0, 39.8)	39.1 (38.6, 39.5)	38.2 (37.8, 38.7)	1.56 (0.564, 2.55)	<0.001	
≥3								
Women:	6.6 (6.1, 7.1)	10.1 (9.8, 10.4)	12.9 (12.6, 13.1)	15.6 (15.3, 15.9)	21.1 (20.8, 21.4)	14.5 (13.9, 15.1)	<0.001	<0.001
Men:	15.1 (14.5, 15.8)	16.8 (16.5, 17.2)	19.3 (18.9, 19.6)	21.6 (21.3, 22.0)	27.6 (27.2, 28.1)	12.5 (11.8, 13.3)	<0.001	

Values are means for continuous variables adjusted for age, unless otherwise specified. Values between brackets indicate 95% confidence intervals, unless otherwise specified. Models further adjusted for number living in participant household.

Household income groups: ≥£100,000; £52,000-<£100,000, £31,000-<£52,000; £18,000-<£31,000, and <£18,000 per year.

BMI = body mass index; CKD = chronic kidney disease; CRP = C-reactive protein; DBP = diastolic blood pressure; eGFR = estimated glomerular filtration rate; HbA1c = glycated haemoglobin; HDL-C = high-density lipoprotein cholesterol; LDL-C = low-density lipoprotein cholesterol; SBP = systolic blood pressure.

[a]*P* values for sex-specific linear trends across household income groups.

[b]*P* values for sex differences in linear trends across Townsend score fifths income groups.

[c]Risk factors included were current smoking, hypertension, obesity, dyslipidaemia and diabetes.

Supplemental Table 14. Cardiometabolic risk factors, treatment, and control, by Townsend Deprivation Score fifths, after excluding participants taking lipid-lowering medication.

	1 (Least deprived)	2	3	4	5 (Most deprived)	Δ between most and least deprived	*P*-Trend[a]
N	155,530	84,083	61,395	54,248	54,984	-	-
**Risk factors**							
SBP, mmHg	137.0 (137.0, 137.1)	137.1 (136.9, 137.2)	136.7 (136.6, 136.8)	136.6 (136.5, 136.8)	136.6 (136.4, 136.8)	-0.451 (-0.632, -0.270)	0.056
DBP, mmHg	82.2 (82.1, 82.2)	82.2 (82.2, 82.3)	82.2 (82.1, 82.3)	82.2 (82.1, 82.3)	82.4 (82.3, 82.5)	0.216 (0.112, 0.321)	0.094
BMI, kg/m2	26.7 (26.7, 26.7)	26.9 (26.9, 27.0)	27.0 (27.0, 27.1)	27.3 (27.3, 27.4)	27.7 (27.7, 27.8)	1.02 (0.979, 1.07)	<0.001
Waist circumference, cm	87.9 (87.8, 88.0)	88.4 (88.3, 88.5)	88.8 (88.7, 88.9)	89.6 (89.5, 89.7)	90.8 (90.7, 90.9)	2.88 (2.75, 3.01)	<0.001
Total cholesterol, mmol/L	5.93 (5.92, 5.93)	5.92 (5.91, 5.92)	5.90 (5.89, 5.91)	5.88 (5.88, 5.89)	5.84 (5.83, 5.85)	-0.087 (-0.097, -0.076)	<0.001
LDL-C, mmol/L	3.73 (3.73, 3.73)	3.73 (3.72, 3.73)	3.71 (3.71, 3.72)	3.71 (3.70, 3.71)	3.69 (3.68, 3.69)	-0.042 (-0.050, -0.034)	0.077
HDL-C, mmol/L	1.49 (1.49, 1.50)	1.49 (1.48, 1.49)	1.48 (1.48, 1.49)	1.47 (1.46, 1.47)	1.44 (1.43, 1.44)	-0.056 (-0.060, -0.052)	<0.001
Triglycerides, mmol/L	1.46 (1.46, 1.47)	1.47 (1.47, 1.48)	1.48 (1.48, 1.49)	1.50 (1.49, 1.51)	1.52 (1.51, 1.52)	0.055 (0.047, 0.063)	0.320
HbA1c, mmol/mol	34.5 (34.5, 34.6)	34.7 (34.6, 34.7)	34.8 (34.8, 34.9)	35.0 (35.0, 35.1)	35.4 (35.4, 35.5)	0.899 (0.855, 0.943)	<0.001
Glucose, mmol/L	4.92 (4.92, 4.92)	4.94 (4.93, 4.94)	4.95 (4.94, 4.96)	4.96 (4.95, 4.97)	4.98 (4.97, 4.98)	0.055 (0.048, 0.063)	0.761
eGFR, mL/min/1.73m^2	95.5 (95.4, 95.5)	95.6 (95.5, 95.6)	95.8 (95.7, 95.9)	95.9 (95.9, 96.0)	96.0 (95.9, 96.1)	0.511 (0.397, 0.625)	0.112
CRP, mg/L	1.26 (1.26, 1.27)	1.32 (1.31, 1.33)	1.36 (1.35, 1.37)	1.46 (1.44, 1.47)	1.63 (1.61, 1.64)	0.369 (0.352, 0.385)	<0.001
**Prevalence, treatment and control**							
Current smoking, %	6.64 (6.52, 6.77)	8.49 (8.30, 8.68)	10.43 (10.19, 10.67)	13.8 (13.5, 14.0)	20.2 (19.8, 20.5)	13.5 (13.2, 13.9)	<0.001
Hypertension, %	49.4 (49.1, 49.6)	50.2 (49.9, 50.6)	49.9 (49.5, 50.3)	50.6 (50.1, 51.0)	52.2 (51.8, 52.6)	2.84 (2.35, 3.34)	<0.001
Treated, %	23.9 (23.6, 24.2)	25.1 (24.7, 25.5)	25.7 (25.1, 26.2)	26.8 (26.2, 27.3)	29.1 (28.5, 29.7)	5.17 (4.52, 5.83)	<0.001
Controlled among treated, %	8.4 (8.2, 8.7)	8.8 (8.5, 9.1)	9.4 (9.1, 9.7)	10.0 (9.6, 10.4)	11.1 (10.7, 11.5)	2.31 (0.952, 3.66)	<0.001
Obesity, %	18.9 (18.7, 19.1)	20.8 (20.5, 21.1)	22.0 (21.7, 22.4)	24.5 (24.1, 24.9)	27.9 (27.5, 28.3)	9.00 (8.58, 9.43)	<0.001
Dyslipidaemia, %	85.3 (85.1, 85.5)	85.5 (85.2, 85.7)	85.4 (85.1, 85.7)	84.9 (84.6, 85.2)	84.5 (84.2, 84.8)	93.7 (91.0, 96.5)	0.034
Treated, %	-	-	-	-	-	-	-
Controlled among treated, %	-	-	-	-	-	-	-
Diabetes, %	0.47 (0.43, 0.50)	0.50 (0.46, 0.55)	0.67 (0.61, 0.74)	0.87 (0.79, 0.96)	1.15 (1.06, 1.25)	0.685 (0.583, 0.787)	0.051
Treated, %	34.8 (32.8, 36.9)	34.0 (31.4, 36.5)	34.8 (31.9, 37.6)	40.2 (37.4, 43.0)	37.4 (35.0, 39.8)	2.58 (-0.598, 5.75)	<0.001
Controlled among treated, %	12.5 (11.1, 14.0)	12.6 (10.9, 14.4)	12.8 (10.8, 14.8)	13.9 (11.9, 15.9)	13.6 (11.9, 15.3)	0.782 (-3.720, 5.28)	<0.001
CKD, %	0.94 (0.89, 0.99)	0.98 (0.91, 1.05)	0.99 (0.91, 1.07)	1.14 (1.04, 1.23)	1.31 (1.21, 1.42)	0.373 (0.257, 0.490)	0.120
**Number of risk factors[b]**							
Number of risk factors	1.44 (1.44, 1.45)	1.49 (1.48, 1.49)	1.52 (1.51, 1.52)	1.58 (1.57, 1.58)	1.67 (1.67, 1.68)	0.232 (0.224, 0.241)	<0.001
Number of risk factors, %							
0	13.6 (13.4, 13.8)	12.9 (12.6, 13.1)	12.5 (12.3, 12.8)	11.7 (11.5, 12.0)	10.3 (10.1, 10.6)	-3.28 (-3.58, -2.98)	<0.001
1	40.5 (40.3, 40.8)	39.2 (38.8, 39.5)	38.3 (37.9, 38.7)	36.5 (36.1, 36.9)	33.4 (33.1, 33.8)	-7.09 (-7.55, -6.62)	<0.001
2	34.7 (34.5, 34.9)	35.1 (34.8, 35.5)	35.2 (34.8, 35.6)	35.7 (35.3, 36.1)	37.2 (36.8, 37.6)	2.48 (2.01, 2.95)	<0.001
≥3	11.3 (11.1, 11.4)	12.9 (12.6, 13.1)	13.9 (13.7, 14.2)	16.1 (15.8, 16.4)	19.3 (19.0, 19.7)	8.08 (7.71, 8.44)	<0.001

Values are means for continuous variables adjusted for age, unless otherwise specified. Values between brackets indicate 95% confidence intervals, unless otherwise specified. Models further adjusted for number living in participant household.

Townsend score fifths: 1 (Townsend score <-2.938, least deprived); 2 (≥-2.938-<-1.531); 3 (≥-1.531-<0.170); 4 (≥0.170-<2.448); and 5 (≥2.448, most deprived).

BMI = body mass index; CKD = chronic kidney disease; CRP = C-reactive protein; DBP = diastolic blood pressure; eGFR = estimated glomerular filtration rate; HbA1c = glycated haemoglobin; HDL-C = high-density lipoprotein cholesterol; LDL-C = low-density lipoprotein cholesterol; SBP = systolic blood pressure.

[a] *P* values for sex-specific linear trends across Townsend score fifths.

[b] Risk factors included were current smoking, hypertension, obesity, dyslipidaemia and diabetes.

Supplemental Table 15. Cardiometabolic risk factors, treatment, and control, by Townsend Deprivation Score fifths and sex, after excluding participants taking lipid-lowering medication.

Risk factors	1 (Least deprived)	2	3	4	5 (Most deprived)	Δ between most and least deprived	P-Trend[a]	P-Het[b]
N								
Women:	89,752	49,068	35,914	31,471	30,332	-	-	-
Men:	65,778	35,015	25,481	22,777	24,452	-	-	-
**Risk factors**								
SBP, mmHg								
Women:	134.6 (134.5, 134.7)	134.6 (134.4, 134.7)	133.9 (133.7, 134.1)	134.0 (133.8, 134.2)	133.8 (133.6, 134.0)	-0.850 (-1.09, -0.613)	<0.001	<0.001
Men:	140.3 (140.2, 140.5)	140.5 (140.4, 140.7)	140.6 (140.4, 140.8)	140.3 (140.0, 140.5)	140.1 (139.9, 140.4)	-0.211 (-0.478, 0.057)	<0.001	
DBP, mmHg								
Women:	80.5 (80.5, 80.6)	80.6 (80.5, 80.7)	80.6 (80.5, 80.7)	80.6 (80.5, 80.7)	80.9 (80.7, 81.0)	0.324 (0.187, 0.460)	<0.001	<0.001
Men:	84.4 (84.3, 84.5)	84.5 (84.4, 84.6)	84.5 (84.3, 84.6)	84.4 (84.2, 84.5)	84.3 (84.2, 84.4)	-0.092 (-0.247, 0.062)	<0.001	
BMI, kg/m^2								
Women:	26.3 (26.2, 26.3)	26.6 (26.5, 26.6)	26.8 (26.7, 26.8)	27.2 (27.1, 27.2)	27.8 (27.8, 27.9)	1.53 (1.47, 1.59)	<0.001	<0.001
Men:	27.2 (27.2, 27.3)	27.4 (27.4, 27.5)	27.4 (27.4, 27.5)	27.5 (27.5, 27.6)	27.6 (27.5, 27.7)	0.353 (0.285, 0.422)	<0.001	
Waist circumference, cm								
Women:	82.5 (82.4, 82.6)	83.2 (83.1, 83.3)	83.9 (83.7, 84.0)	85.0 (84.9, 85.1)	86.5 (86.4, 86.6)	4.00 (3.85, 4.15)	<0.001	<0.001
Men:	95.2 (95.2, 95.3)	95.7 (95.5, 95.8)	95.8 (95.7, 96.0)	96.1 (95.9, 96.2)	96.2 (96.0, 96.3)	0.913 (0.743, 1.08)	<0.001	
Total cholesterol, mmol/L								
Women:	6.03 (6.02, 6.04)	6.02 (6.01, 6.03)	5.99 (5.98, 6.00)	5.97 (5.96, 5.98)	5.92 (5.91, 5.94)	-0.106 (-0.120, -0.092)	<0.001	<0.001
Men:	5.79 (5.78, 5.79)	5.77 (5.76, 5.78)	5.78 (5.77, 5.79)	5.77 (5.75, 5.78)	5.73 (5.72, 5.75)	-0.052 (-0.068, -0.037)	<0.001	
LDL-C, mmol/L								
Women:	3.74 (3.73, 3.74)	3.74 (3.73, 3.75)	3.72 (3.71, 3.72)	3.71 (3.70, 3.72)	3.70 (3.69, 3.71)	-0.040 (-0.050, -0.029)	<0.001	0.168
Men:	3.72 (3.71, 3.73)	3.71 (3.70, 3.72)	3.71 (3.70, 3.72)	3.70 (3.69, 3.71)	3.68 (3.67, 3.69)	-0.045 (-0.040, -0.050)	<0.001	
HDL-C, mmol/L								
Women:	1.63 (1.63, 1.64)	1.62 (1.62, 1.62)	1.61 (1.61, 1.61)	1.59 (1.59, 1.59)	1.56 (1.55, 1.56)	-0.078 (-0.083, -0.073)	<0.001	<0.001
Men:	1.31 (1.30, 1.31)	1.30 (1.30, 1.31)	1.31 (1.30, 1.31)	1.30 (1.30, 1.31)	1.29 (1.29, 1.30)	-0.016 (-0.021, -0.010)	<0.001	
Triglycerides, mmol/L								
Women:	1.32 (1.31, 1.32)	1.33 (1.33, 1.34)	1.34 (1.33, 1.35)	1.36 (1.35, 1.37)	1.38 (1.37, 1.39)	0.061 (0.052, 0.070)	<0.001	<0.001
Men:	1.68 (1.68, 1.69)	1.70 (1.69, 1.71)	1.71 (1.70, 1.72)	1.71 (1.70, 1.72)	1.71 (1.70, 1.72)	0.027 (0.014, 0.040)	<0.001	
HbA1c, mmol/mol								
Women:	34.6 (34.6, 34.6)	34.7 (34.6, 34.7)	34.8 (34.7, 34.8)	35.0 (34.9, 35.0)	35.3 (35.3, 35.4)	0.728 (0.669, 0.787)	<0.001	<0.001
Men:	34.5 (34.5, 34.5)	34.7 (34.6, 34.7)	34.9 (34.8, 34.9)	35.1 (35.1, 35.2)	35.6 (35.6, 35.7)	1.12 (1.05, 1.18)	<0.001	
Glucose, mmol/L								
Women:	4.92 (4.91, 4.92)	4.93 (4.92, 4.94)	4.94 (4.93, 4.95)	4.94 (4.93, 4.95)	4.95 (4.94, 4.96)	0.032 (0.022, 0.042)	<0.001	<0.001

Men:	4.93 (4.92, 4.93)	4.94 (4.93, 4.95)	4.97 (4.96, 4.98)	4.99 (4.98, 5.00)	5.01 (5.00, 5.02)	0.084 (0.073, 0.095)	<0.001	
eGFR, mL/min/1.73m^2								
Women:	95.4 (95.4, 95.5)	95.4 (95.3, 95.5)	95.6 (95.4, 95.7)	95.5 (95.4, 95.7)	95.4 (95.3, 95.5)	-0.012 (-0.164, 0.140)	<0.001	<0.001
Men:	95.5 (95.4, 95.6)	95.8 (95.6, 95.9)	96.1 (96.0, 96.3)	96.5 (96.4, 96.7)	96.7 (96.5, 96.8)	1.16 (0.988, 1.33)	<0.001	
CRP, mg/L								
Women:	1.29 (1.28, 1.30)	1.34 (1.33, 1.35)	1.38 (1.37, 1.40)	1.50 (1.48, 1.51)	1.67 (1.65, 1.69)	0.384 (0.361, 0.407)	<0.001	0.115
Men:	1.23 (1.22, 1.24)	1.29 (1.28, 1.31)	1.33 (1.31, 1.34)	1.40 (1.38, 1.42)	1.58 (1.56, 1.60)	0.353 (0.329, 0.377)	<0.001	
**Prevalence, treatment and control**								
Current smoking, %								
Women:	5.49 (5.34, 5.64)	7.20 (6.97, 7.43)	9.00 (8.70, 9.29)	11.8 (11.4, 12.2)	17.2 (16.8, 17.6)	11.7 (11.2, 12.1)	<0.001	0.248
Men:	8.21 (8.00, 8.43)	10.3 (9.97, 10.6)	12.4 (12.0, 12.8)	16.4 (16.0, 16.9)	23.9 (23.4, 24.4)	15.7 (15.2, 16.3)	<0.001	
Hypertension, %								
Women:	44.5 (44.2, 44.8)	45.2 (44.8, 45.7)	44.8 (44.2, 45.3)	45.7 (45.2, 46.3)	47.0 (46.4, 47.6)	2.53 (1.88, 3.19)	0.004	0.512
Men:	56.0 (55.6, 56.4)	57.3 (56.7, 57.8)	57.0 (56.4, 57.6)	57.3 (56.6, 57.9)	58.7 (58.1, 59.3)	2.69 (1.96, 3.42)	0.023	
Treated, %								
Women:	25.5 (25.1, 26.0)	27.2 (26.6, 27.8)	27.8 (27.0, 28.5)	29.2 (28.4, 30.0)	32.4 (31.6, 33.2)	6.93 (5.99, 7.88)	<0.001	0.104
Men:	22.1 (21.7, 22.6)	22.8 (22.2, 23.4)	23.2 (22.5, 24.0)	23.9 (23.2, 24.7)	25.6 (24.8, 26.4)	3.45 (2.56, 4.34)	<0.001	
Controlled among treated, %								
Women:	9.9 (9.6, 10.2)	10.5 (10.1, 10.9)	11.2 (10.7, 11.7)	12.1 (11.5, 12.7)	13.4 (12.8, 14.0)	2.31 (0.492, 4.14)	<0.001	0.011
Men:	6.9 (6.6, 7.2)	7.0 (6.6, 7.3)	7.4 (6.9, 7.8)	7.6 (7.1, 8.1)	8.6 (8.1, 9.1)	2.23 (0.229, 4.22)	<0.001	
Obesity, %								
Women:	18.1 (17.8, 18.3)	19.9 (19.5, 20.2)	21.7 (21.3, 22.1)	25.1 (24.6, 25.5)	29.6 (29.1, 30.1)	11.5 (11.0, 12.1)	<0.001	<0.001
Men:	20.0 (19.7, 20.3)	22.1 (21.6, 22.5)	22.5 (22.0, 23.0)	23.7 (23.1, 24.2)	25.8 (25.2, 26.3)	5.73 (5.10, 6.36)	0.005	
Dyslipidaemia, %								
Women:	86.5 (86.3, 86.8)	86.7 (86.4, 87.0)	86.4 (86.0, 86.8)	86.0 (85.6, 86.4)	85.7 (85.3, 86.1)	93.3 (89.6, 97.2)	0.532	0.724
Men:	83.7 (83.4, 84.0)	83.8 (83.4, 84.2)	84.0 (83.5, 84.4)	83.4 (82.9, 83.9)	83.0 (82.6, 83.5)	95.1 (91.1, 99.2)	0.130	
Treated, %								
Women:	-	-	-	-	-	-	-	-
Men:	-	-	-	-	-	-	-	
Controlled among treated, %-								
Women:	-	-	-	-	-	-	-	-
Men:	-	-	-	-	-	-	-	
Diabetes, %								
Women:	0.31 (0.28, 0.35)	0.42 (0.36, 0.48)	0.53 (0.45, 0.61)	0.70 (0.60, 0.80)	0.90 (0.78, 1.01)	0.585 (0.465, 0.704)	0.985	0.020
Men:	0.68 (0.61, 0.74)	0.62 (0.54, 0.71)	0.87 (0.75, 0.99)	1.12 (0.97, 1.26)	1.48 (1.31, 1.64)	0.797 (0.623, 0.971)	0.003	
Treated, %								
Women:	30.6 (27.7, 33.6)	31.8 (28.2, 35.4)	32.5 (28.5, 36.6)	40.5 (36.3, 44.6)	35.5 (32.0, 39.1)	4.90 (0.284, 9.51)	<0.001	0.186
Men:	38.3 (35.5, 41.2)	36.0 (32.4, 39.6)	36.7 (32.8, 40.7)	39.9 (36.0, 43.7)	38.8 (35.6, 42.1)	0.481 (-3.84, 4.81)	<0.001	
Controlled among treated, %								

Women:	11.0 (9.0, 13.0)	11.3 (8.9, 13.8)	11.3 (8.6, 14.0)	14.2 (11.3, 17.2)	11.9 (9.5, 14.3)	-1.15 (-7.95, 5.65)	<0.001	0.572
Men:	13.9 (11.9, 15.9)	13.9 (11.3, 16.5)	14.2 (11.3, 17.0)	13.6 (10.9, 16.2)	15.0 (12.6, 17.4)	2.24 (-3.73, 8.21)	<0.001	
CKD, %								
Women:	1.04 (0.97, 1.10)	1.08 (0.98, 1.17)	1.04 (0.93, 1.15)	1.24 (1.11, 1.37)	1.41 (1.26, 1.55)	0.372 (0.211, 0.533)	0.112	0.740
Men:	0.81 (0.74, 0.88)	0.85 (0.75, 0.95)	0.93 (0.80, 1.05)	1.00 (0.86, 1.13)	1.19 (1.04, 1.35)	0.381 (0.214, 0.547)	0.694	
**Number of risk factors[c]**								
Number of risk factors								
Women:	1.38 (1.37, 1.39)	1.42 (1.41, 1.43)	1.45 (1.44, 1.46)	1.52 (1.51, 1.53)	1.62 (1.61, 1.63)	0.236 (0.224, 0.248)	<0.001	0.010
Men:	1.53 (1.52, 1.54)	1.58 (1.57, 1.59)	1.61 (1.60, 1.62)	1.65 (1.64, 1.67)	1.75 (1.74, 1.76)	0.221 (0.208, 0.234)	<0.001	
Number of risk factors, %								
0								
Women:	15.1 (14.8, 15.3)	14.3 (14.0, 14.6)	13.9 (13.6, 14.3)	12.9 (12.6, 13.3)	11.77 (11.43, 12.1)	-3.26 (-3.68, -2.85)	0.025	0.309
Men:	11.68 (11.43, 11.92)	10.87 (10.55, 11.20)	10.62 (10.25, 10.99)	10.11 (9.74, 10.49)	8.57 (8.24, 8.90)	-3.13 (-3.55, -2.71)	0.001	
1								
Women:	42.5 (42.2, 42.8)	41.2 (40.8, 41.6)	40.4 (39.9, 40.9)	38.0 (37.5, 38.6)	34.6 (34.1, 35.1)	-7.91 (-8.53, -7.28)	<0.001	<0.001
Men:	37.9 (37.5, 38.2)	36.3 (35.8, 36.8)	35.3 (34.8, 35.9)	34.4 (33.8, 35.0)	32.0 (31.4, 32.6)	-5.86 (-6.55, -5.17)	0.070	
2								
Women:	32.9 (32.6, 33.2)	33.4 (33.0, 33.9)	33.2 (32.8, 33.7)	34.4 (33.9, 34.9)	35.9 (35.3, 36.4)	3.02 (2.40, 3.64)	<0.001	<0.001
Men:	37.2 (36.8, 37.6)	37.5 (37.0, 38.0)	38.0 (37.4, 38.6)	37.5 (36.8, 38.1)	38.8 (38.2, 39.4)	1.61 (0.898, 2.32)	0.147	
≥3								
Women:	9.8 (9.6, 10.0)	11.2 (10.9, 11.4)	12.4 (12.1, 12.8)	14.6 (14.3, 15.0)	17.9 (17.4, 18.3)	8.05 (7.57, 8.53)	<0.001	<0.001
Men:	13.2 (13.0, 13.5)	15.2 (14.9, 15.6)	16.1 (15.6, 16.5)	18.2 (17.7, 18.7)	21.2 (20.7, 21.7)	7.96 (7.39, 8.54)	0.001	

Values are means for continuous variables adjusted for age, unless otherwise specified. Values between brackets indicate 95% confidence intervals, unless otherwise specified.

Townsend score fifths: 1 (Townsend score <-2.938, least deprived); 2 (≥-2.938-<-1.531); 3 (≥-1.531-<0.170); 4 (≥0.170-<2.448); and 5 (≥2.448, most deprived).

BMI = body mass index; CKD = chronic kidney disease; CRP = C-reactive protein; DBP = diastolic blood pressure; eGFR = estimated glomerular filtration rate; HbA1c = glycated haemoglobin; HDL-C = high-density lipoprotein cholesterol; LDL-C = low-density lipoprotein cholesterol; SBP = systolic blood pressure.

[a]*P* values for sex-specific linear trends across Townsend score fifths.

[b]*P* values for sex differences in linear trends across Townsend score fifths.

[c]Risk factors included were current smoking, hypertension, obesity, dyslipidaemia and diabetes.

REFERENCES

1 Inker LA, Eneanya ND, Coresh J, et al. New Creatinine- and Cystatin C-Based Equations to Estimate GFR without Race. N Engl J Med 2021;385:1737-49.

2 National Institute for Health and Care Excellence. Hypertension in adults: diagnosis and management [online]. 2019. https://www.nice.org.uk/guidance/ng136 (accessed 3 March 2025).

3 National Institute for Health and Care Excellence. Cardiovascular disease: risk assessment and reduction, including lipid modification [online]. 2023. https://www.nice.org.uk/guidance/ng238 (accessed 3 March 2025).

4 National Institute for Health and Care Excellence. Type 2 diabetes in adults: management [online]. 2015. https://www.nice.org.uk/guidance/ng28 (accessed 3 March 2025).
